# Synthesis of Elaborate Benzofuran-2-Carboxamide Derivatives through a Combination of 8-Aminoquinoline Directed C–H Arylation and Transamidation Chemistry

**DOI:** 10.3390/molecules25020361

**Published:** 2020-01-15

**Authors:** Michael Oschmann, Linus Johansson Holm, Monireh Pourghasemi-Lati, Oscar Verho

**Affiliations:** Arrhenius Laboratory, Department of Organic Chemistry, Stockholm University, SE-106 91 Stockholm, Sweden; michael.oschmann@su.se (M.O.); linus.johansson@su.se (L.J.H.); monireh.lati@su.se (M.P.-L.)

**Keywords:** palladium, C–H functionalization, 8-aminoquinoline, benzofuran, transamidation

## Abstract

Herein, we present a short and highly modular synthetic route that involves 8-aminoquinoline directed C–H arylation and transamidation chemistry, and which enables access to a wide range of elaborate benzofuran-2-carboxamides. For the directed C–H arylation reactions, Pd catalysis was used to install a wide range of aryl and heteroaryl substituents at the C3 position of the benzofuran scaffold in high efficiency. Directing group cleavage and further diversification of the C3-arylated benzofuran products were then achieved in a single synthetic operation through the utilization of a one-pot, two-step transamidation procedure, which proceeded via the intermediate *N*-acyl-Boc-carbamates. Given the high efficiency and modularity of this synthetic strategy, it constitutes a very attractive method for generating structurally diverse collections of benzofuran derivatives for small molecule screening campaigns.

## 1. Introduction

**Figure 1:**
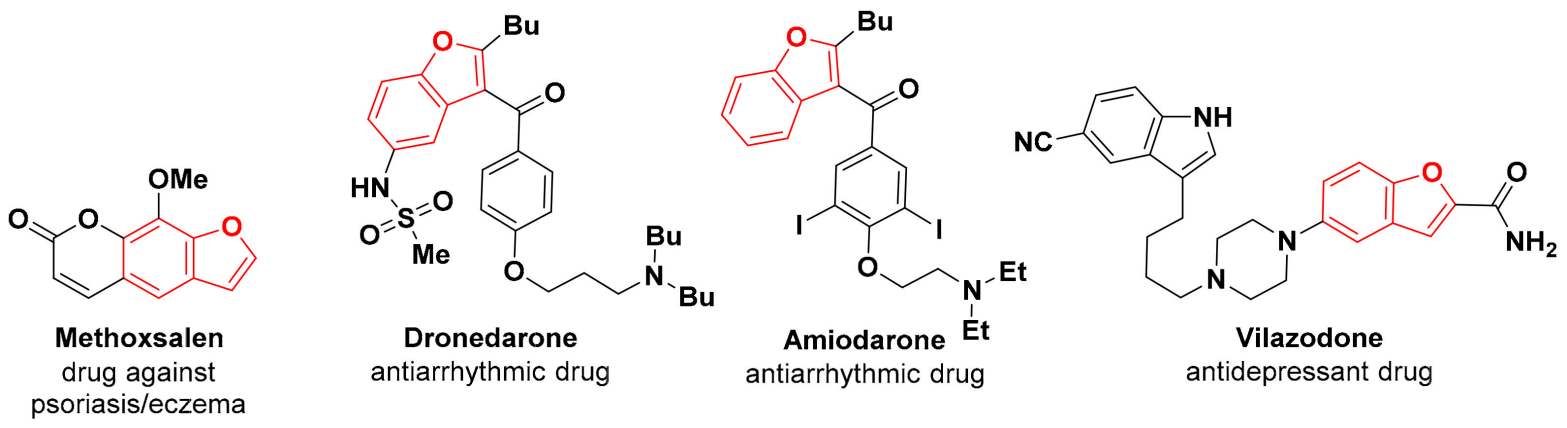
Examples of drugs containing the benzofuran scaffold.

The benzofuran core is present in many biologically active natural products, which has made it a popular scaffold to explore when designing drugs [1,2,3,4]. Today, a large number of benzofuran-based drugs are available on the market, including examples such as Methoxsalen that is used against psoriasis and eczema, the antiarrhythmic medications Amiodarone and Dronedarone, as well as the antidepressant Vilazodone (Figure 1) [1,2,3,4]. Furthermore, other benzofuran derivatives have been shown to display a wide range of antimicrobial and anticancer properties [1,2,3,4,5,6,7]. Given the vast number of applications of benzofuran-based drugs in today’s medicine, there exists considerable interest for novel synthetic methodologies that can grant expedient access to new kinds of benzofuran derivatives.

**Scheme 1:**
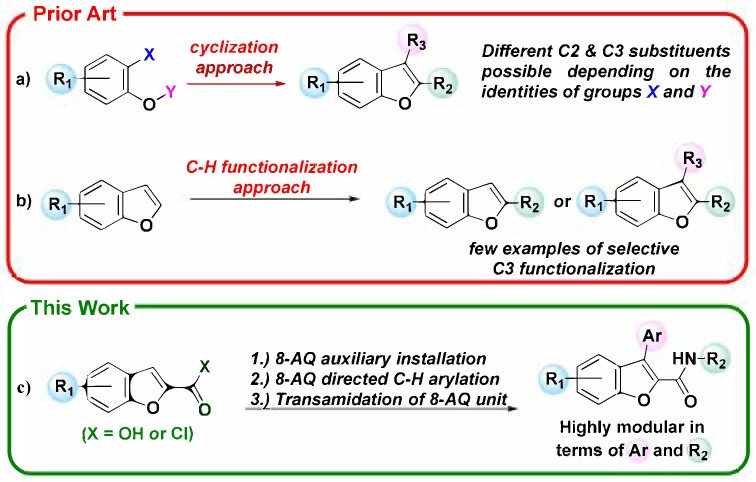
Different synthetic strategies to access highly functionalized benzofurans. (**a**,**b**) General representations of prior cyclization- and C–H functionalization-based approaches. (**c**) The herein described approach towards C3-substituted benzofuran-2-carboxamide derivatives enabled by a combination of 8-AQ directed C–H arylation and transamidation chemistry.

The benzofuran scaffold is a heterocyclic motif that consists of a fused benzene and furan ring, and many different synthetic strategies have been used in the past to introduce substituents on these two parts (Scheme 1a,b) [8,9,10]. Substituents at the benzene moiety often originate from the synthetic precursor used to construct the benzofuran scaffold. Synthetic methods for assembling the benzofuran scaffold have typically involved acid-catalyzed cyclizations [11,12,13,14,15,16,17], carbonylative cyclizations via Sonogashira reactions [18,19,20,21,22,23,24], Heck-type cyclizations [25,26,27,28,29], photocyclizations [30,31,32,33,34], radical cyclizations [35,36,37,38,39], and other types of transition-metal catalyzed transformations [40,41,42,43,44,45]. When it comes to the functionalization of the furan part, there exists a considerable number of methods for directly functionalizing either the C2 or C3 position [46,47,48,49,50,51,52,53,54,55,56]. Of these two positions, C2 is generally easier to functionalize as it is significantly more reactive than C3 [57,58].

In our group, we identified an interesting opportunity to access a diverse set of benzofuran-2-carboxamide derivatives housing different substituents in the C3 position, by combining 8-aminoquinoline (8-AQ) directed C–H functionalization chemistry with a two-step transamidation protocol for 8-AQ amides that had been previously developed within our lab (Scheme 1c) [59]. Since 8-AQ directed C–H functionalization chemistry [60,61,62] allows for rapid assembly of molecular complexity, it has found extensive use within the fields of drug discovery [63,64,65,66,67,68,69,70] and natural product synthesis [71,72,73,74,75,76,77]. Inspired by this prior art, we sought to leverage this powerful methodology when designing a modular and robust synthetic route towards our target benzofuran-2-carboxamide derivatives.

In our envisioned synthetic approach, it was planned to start from the simple and commercially available building block, benzofuran-2-carboxylic acid, into which the 8-AQ auxiliary would be installed. The 8-AQ amide **1a** was anticipated to be a suitable substrate for Pd-catalyzed C–H functionalization chemistry, which can, for example, be used to install different aryl and heteroaryl substituents into the adjacent C3 position. Then, by taking advantage of our previously developed transamidation procedure designed specifically for 8-AQ amides [59], it would be possible to replace the 8-AQ auxiliary with different amine groups in a straightforward fashion, enabling access to a diversity of amide derivatives. Here, the transamidation is achieved over two steps, where the 8-AQ amide is first treated with di-*tert*-butyl dicarbonate (Boc_2_O) and 4-dimethylaminopyridine (DMAP) to form the corresponding *N*-acyl-Boc-carbamate intermediate, which is then reacted with an amine to produce the desired product amide. An interesting feature of this latter aminolysis step is that it proceeds efficiently at mild temperatures without the need of any catalyst or additive. This transamidation approach, proceeding via the intermediate *N*-acyl-Boc-carbamates, has also been used lately by other groups to transamidate other types of amide derivatives [75,78,79,80].

## 2. Results

To install the 8-AQ auxiliary into benzofuran-2-carboxylic acid, a coupling procedure involving HATU and *N*,*N*-diisopropylethylamine in CH_2_Cl_2_ was used, which furnished the desired 8-AQ amide substrate **1a** in 73% yield after 5 h (for further details, see Supporting Materials, SM) [81]. With 8-AQ amide **1a** in hand, we next turned our attention to the C–H arylation reaction, and here our initial efforts were focused on identifying reaction conditions under which this transformation proceeded efficiently. In this optimization study, the effects of several reaction parameters on the C–H arylation of **1a** with 4-iodoanisole were investigated (Table 1). The reaction conditions for the first trial experiment were selected based on a C–H functionalization protocol for furans that had previously been reported by Padmavathi et al. [82]. When performing the C–H arylation of **1a** with 3 equiv. 4-iodoanisole, using Pd(OAc)_2_ (5 mol%) and AgOAc (1.5 equiv.) in toluene (0.5 M) at 110 °C for 7 h, a promising first yield of 46% of product **2a** was obtained (Entry 1). Expectedly, extending the reaction time to 16 h resulted in a higher yield of product **2a** (65% yield, Entry 2). However, it was noted that an increase of the reaction temperature to 120 °C led to a reduced yield of product **2a** over 7 h (30%, Entry 3). The choice of additive, on the other hand, was found to have a more pronounced positive impact on our model reaction. For example, when 0.2 equiv. of pivalic acid (PivOH) was used as an additive, the yield of product **2a** was improved to 61% after 7 h (Entry 4). Unfortunately, the use of increased amounts of PivOH (0.5 equiv. and 1.0 equiv.) did not offer any further improvements in terms of the yield of product **2a** (Entries 5 and 6). The addition of NaOAc was also found to be beneficial for the C–H arylation reaction (Entries 8–10), and here 1 equiv. appeared to be the optimal amount, which furnished product **2a** in a high yield of 78% after 7 h. Unfortunately, the reaction with both 0.25 equiv. PivOH and 1.0 equiv. NaOAc was found to perform worse than the one with only 1.0 equiv. of NaOAc as the additive (56% vs. 78%, Entries 11 and 9).

No further improvements of the model C–H arylation reaction were observed upon variation of the Pd source (see SM, Table S1). Furthermore, Ag_2_CO_3_ was found to be an inferior silver source compared to AgOAc, as it only allowed for product **2a** to be obtained in 49% yield after 7 h (see SM, Table S1).

However, we were delighted to see that this model reaction could be further improved through change of the solvent (Entries 12–16). The green solvents *tert*-amyl alcohol (*t*-amyl OH), methyl-THF (MeTHF), and cyclopentyl methyl ether (CPME) all proved to be better options than toluene (entries 12–14 vs. Entry 9). Of these solvents, on the one hand, CPME allowed for the highest yield of product **2a** after 7 h (93%, Entry 14). On the other hand, lower yields of product **2a** were observed when 1,2-dichloroethane (DCE) and acetonitrile (MeCN) were used as solvents (Entries 15 and 16). 

We also tested if it was possible to use a smaller excess of 4-iodoanisole (2 equiv.) and compensate this with either a longer reaction time or higher reaction concentration (Entries 17 and 18). Unfortunately, these alterations of the reaction conditions led to markedly lower yields of **2a** (80% and 73% yield, respectively, after 15 h). 

**Scheme 2:**
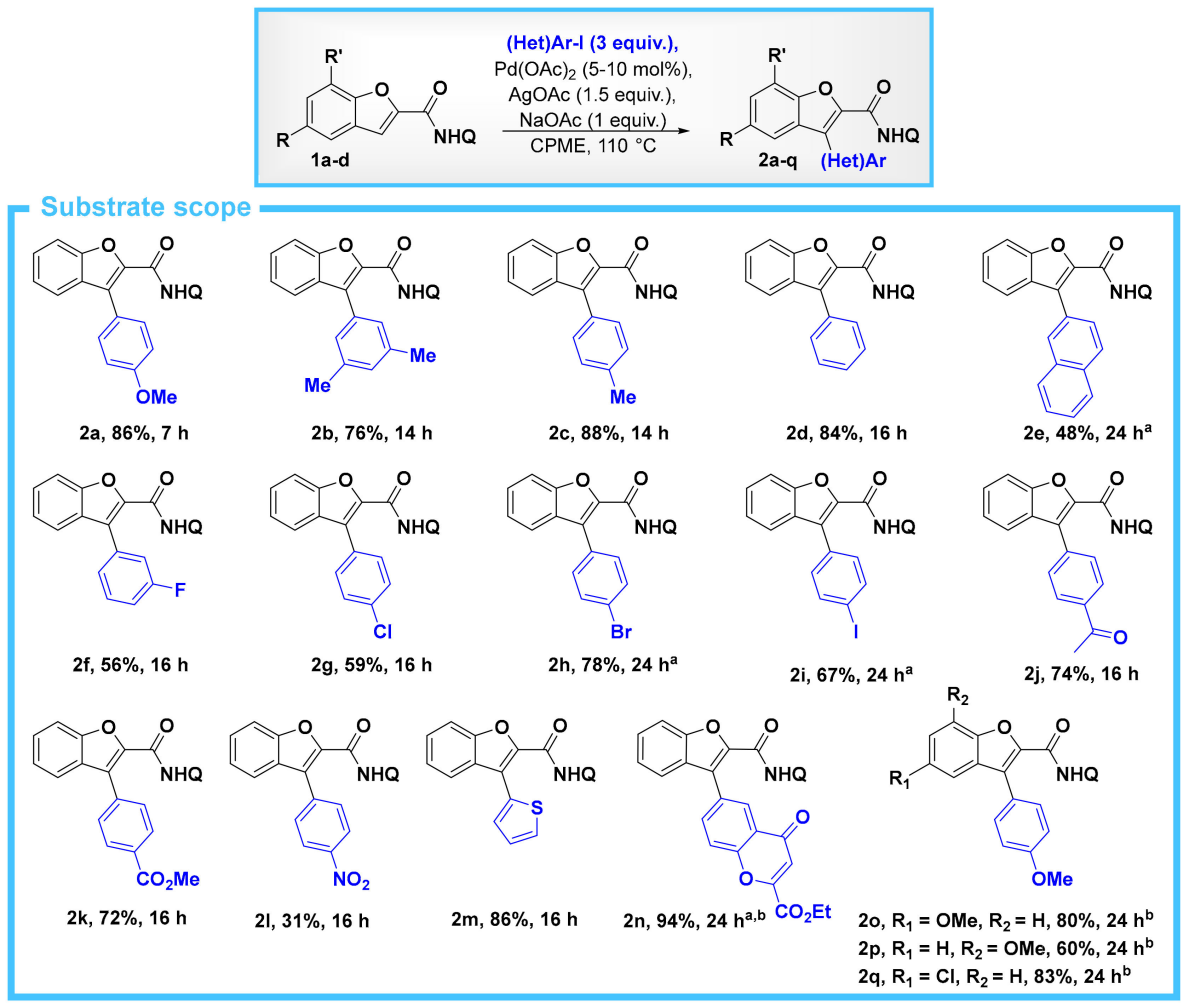
Scope of the C–H arylation protocol in terms of different (hetero)aryl iodides and benzofuran substrates. Reagents and conditions: Benzofuran substrate (0.15 mmol), (hetero)aryl iodide (3.0 equiv.), Pd(OAc)_2_ (5 mol%), AgOAc (1.5 equiv.), and NaOAc (1 equiv.) were all dissolved in CPME (0.5 M) and heated at 110 °C under inert atmosphere for the times given above. All reported yields refer to isolated yield (see SM for further details). ^[a]^ Soxhlet extraction with CH_2_Cl_2_ was used in the purification procedure. ^[b]^ 10 mol% Pd(OAc)_2_ was used.

On the basis of the results from our optimization study, it was decided to begin the substrate scope evaluation using the following conditions: aryl iodide (3 equiv.), Pd(OAc)_2_ (5 mol%), AgOAc (1.5 equiv.), and NaOAc (1 equiv.) in CPME (0.5 M) at 110 °C. As a part of this substrate scope study, both different aryl iodides and different benzofuran substrates were tested (Scheme 2). Here, some of the formed C–H arylation products were found to display limited solubility in a number of commonly available organic solvents (**2e**, **2h**, **2i**, and **2n**), which prevented purification by column chromatography [83]. For these C–H arylation products, an alternate purification procedure was developed in which the crude reaction mixtures were first loaded onto a silica pad and where the remaining starting material and excess aryl iodide were then eluted with an ethyl acetate wash. To recover the products from the silica pads, they were transferred to a Soxhlet apparatus and extracted overnight with CH_2_Cl_2_. With this alternative purification protocol, we could also obtain these more problematic C–H arylation products in high yields and excellent purity.

In terms of aryl iodides, the C–H arylation protocol was found to benefit from those carrying electron-donating substituents. This trend was best exemplified by the reaction to form product **2a**, which could be isolated in 86% yield after 7 h. Good results were also obtained when using 5-iodo-*meta*-xylene and 4-iodotoluene, and from these reactions products **2b** and **2c** could be isolated in 76% and 88% yield, respectively, after 14 h. A slightly longer reaction time (16 h) was required for the arylation with iodobenzene, but also here a high yield of the C–H product was obtained (**2d**, 84%). However, in the cases of the reactions with 2-iodonaphthalene and aryl iodides carrying additional halogen substituents, lower yields of the desired C–H arylation products **2e**–**i** were typically observed (48% to 78% yield after 16 to 24 h). On the other hand, the reactions involving aryl iodides with a keto or ester group proceeded well, and here products **2j** and **2k** were acquired in 74% and 72% yield, respectively, after 16 h. Unfortunately, the reaction using 1-iodo-4-nitrobenzene as the arylating agent gave a complicated mixture of products from which only limited amounts of the desired product **2l** could be isolated (31% yield). To our delight, it also proved possible to install different heteroaromatic moieties using our catalytic protocol, as demonstrated by the reactions to form the thiophene- and chromone-based products **2m** and **2n** in 86% and 94% yield, respectively. However, for the latter reaction it proved necessary to use 10 mol% Pd(OAc)_2_ and a reaction time of 24 h in order to ensure a high yield of product **2n**.

Higher catalyst loadings and longer reactions times were also required when carrying out the C-H arylation on different substituted benzofuran substrates. However, by using these more forcing conditions we were able to show that this catalytic protocol can also be used to prepare the 5-OMe-, 7-OMe-, and 5-Cl-substituted products **2o**–**q** in respectable yields (80%, 60%, and 83%, respectively).

**Scheme 3:**
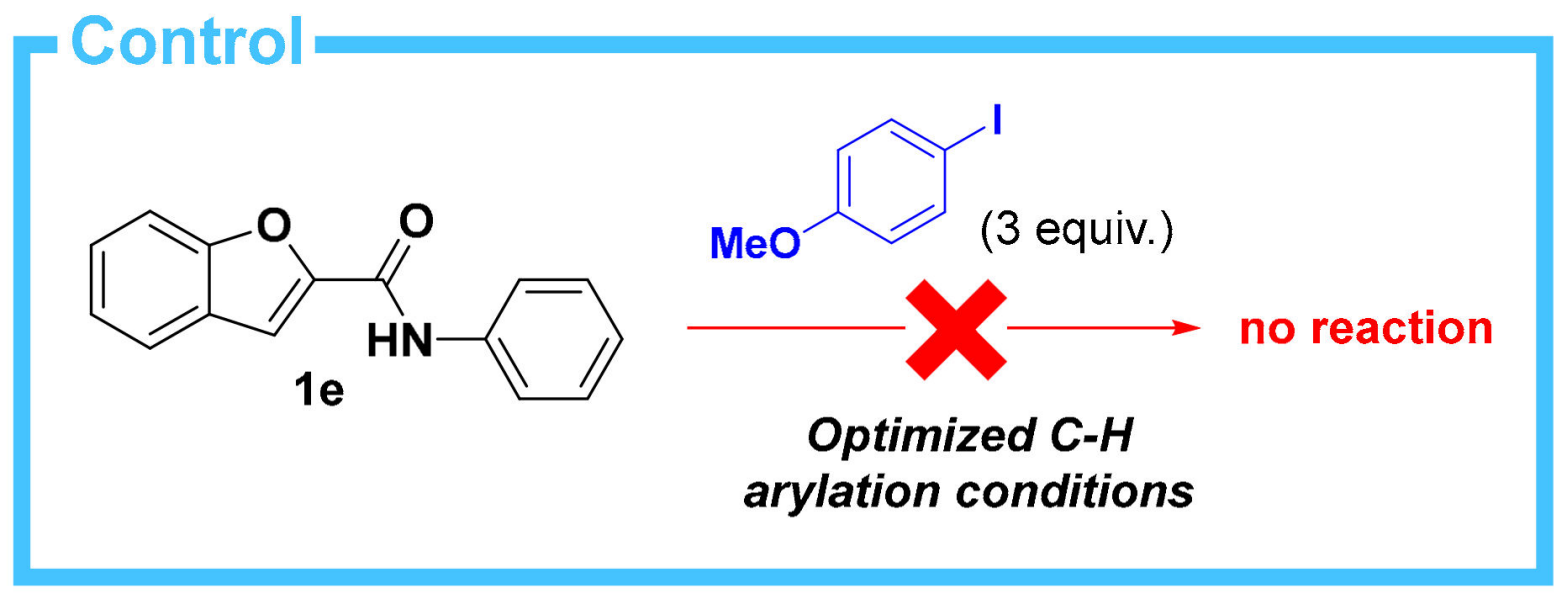
Control experiment with substrate lacking the 8-AQ directing group.

Furthermore, to demonstrate that the 8-AQ auxiliary is needed for mediating the observed C–H arylation reactions, a control experiment with the truncated benzofuran amide derivative **1e** was performed (Scheme 3). Here, we could find that no C–H arylation took place in the absence of a directing quinoline moiety, which confirms that the 8-AQ auxiliary is indeed needed for enabling these reactions. 

**Scheme 4:**
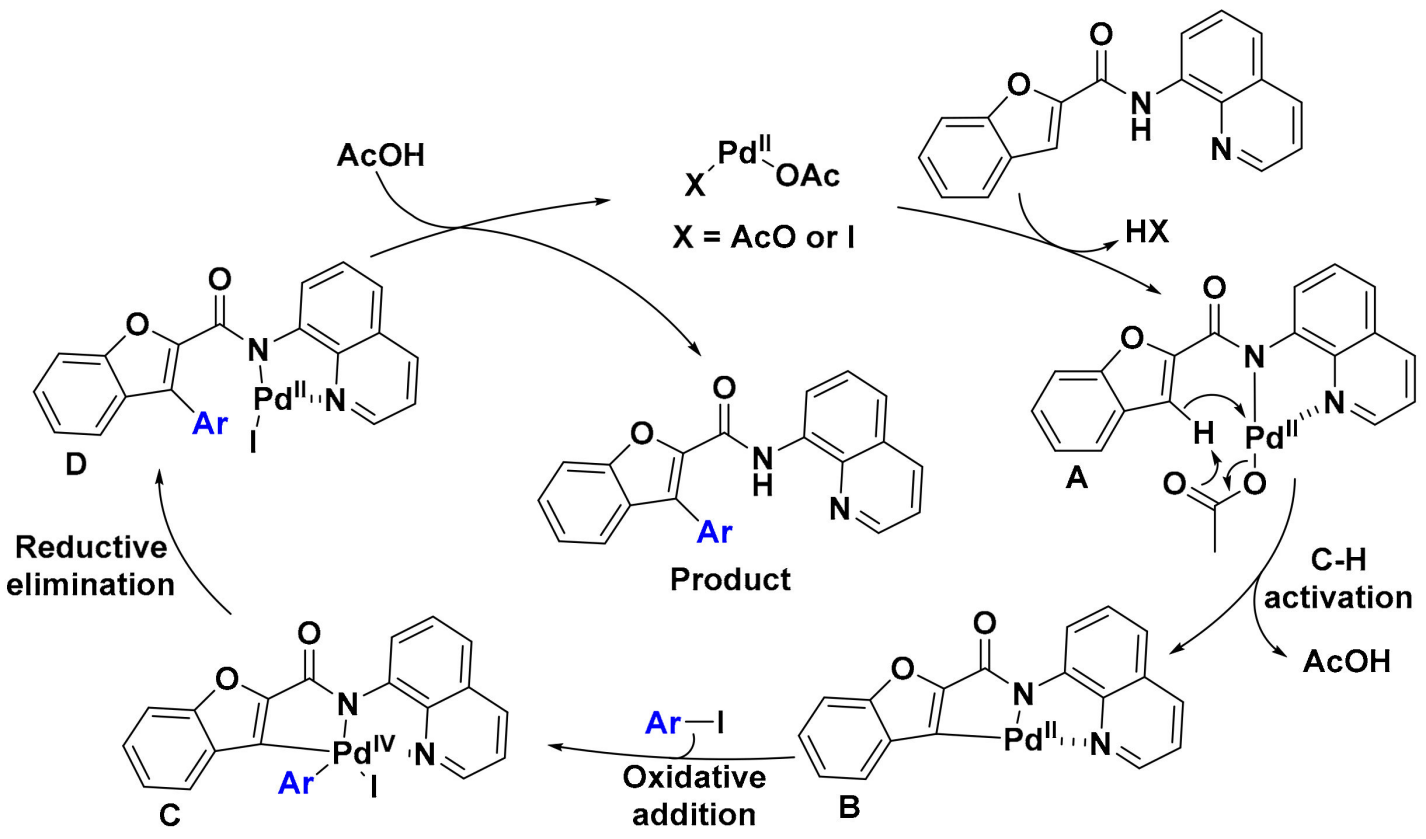
Proposed mechanism for the Pd-catalyzed, 8-AQ-assisted C3 arylation of the benzofurans.

Regarding the mechanism of the C–H arylation of these benzofuran derivatives, it is proposed to proceed via a Pd(II)/Pd(IV) cycle similar to those of other previously reported 8-AQ-directed C–H functionalization reactions (Scheme 4) [60,61,62,84]. The catalytic cycle begins with coordination of Pd(OAc)_2_ to the substrate, which leads to the formation of intermediate **A** and the liberation of HX (where X = OAc or I). In the next step, C–H activation occurs concomitantly with the release of AcOH to give the palladacycle **B**. This is followed by oxidative addition of the aryl iodide to **B**, which furnishes the Pd(IV)-intermediate **C** that, then, undergoes reductive elimination to form the arylation product-bound Pd(II)-complex **D**. Finally, the catalytic cycle is closed by a protodemetalation process triggered by AcOH, which leads to the release of the C–H arylation product and the regeneration of an active Pd catalyst. 

However, it should be pointed out that this catalytic cycle does not describe the exact role of AgOAc. As can be seen from our own control experiments summarized in Table S2, AgOAc is an essential co-additive for our C–H arylation reaction, which worked really poorly in the absence of AgOAc (only 5% yield of **2a** was obtained in this case). Moreover, these control experiments showed that 1.5 equiv. AgOAc was indeed necessary for achieving an efficient C–H arylation, as reduced amounts of AgOAc resulted in markedly lower yields of **2a**. Previous studies have suggested that Ag additives can play many important roles in C–H functionalization reactions, for example, by serving as bases or halide scavengers [85,86], or as terminal oxidants [87]. More recently, it has also been proposed that the Ag might, in fact, have much deeper roles in the mechanisms of different C–H functionalization reactions, by for example enabling the formation of different bimetallic Ag-Pd species that constitutes key intermediates in their respective catalytic cycles [88,89,90].

Having surveyed the scope of the C–H arylation reaction, we next turned our attention to the removal of the 8-AQ auxiliary, using our previously developed two-step transamidation procedure [59]. By applying this transamidation procedure to products such as those in Scheme 2, it is possible to access a wide range of structurally complex, C3-substituted benzofuran-2-carboxamides, in only three synthetic operations from a simple precursor such as benzofuran-2-carboxylic acid (i.e., 1. 8-AQ installation, 2. C–H arylation, and 3. transamidation). In our previous study, most of the transamidations were carried out as two separated reaction steps, where the intermediate *N*-acyl-Boc-carbamates were isolated in between by column chromatography. However, in that study we could show for one example that our transamidation protocol had the potential to be carried out as a two-step one-pot sequence, in which the *N*-acyl-Boc-carbamate did not need to be isolated.

**Scheme 5:**
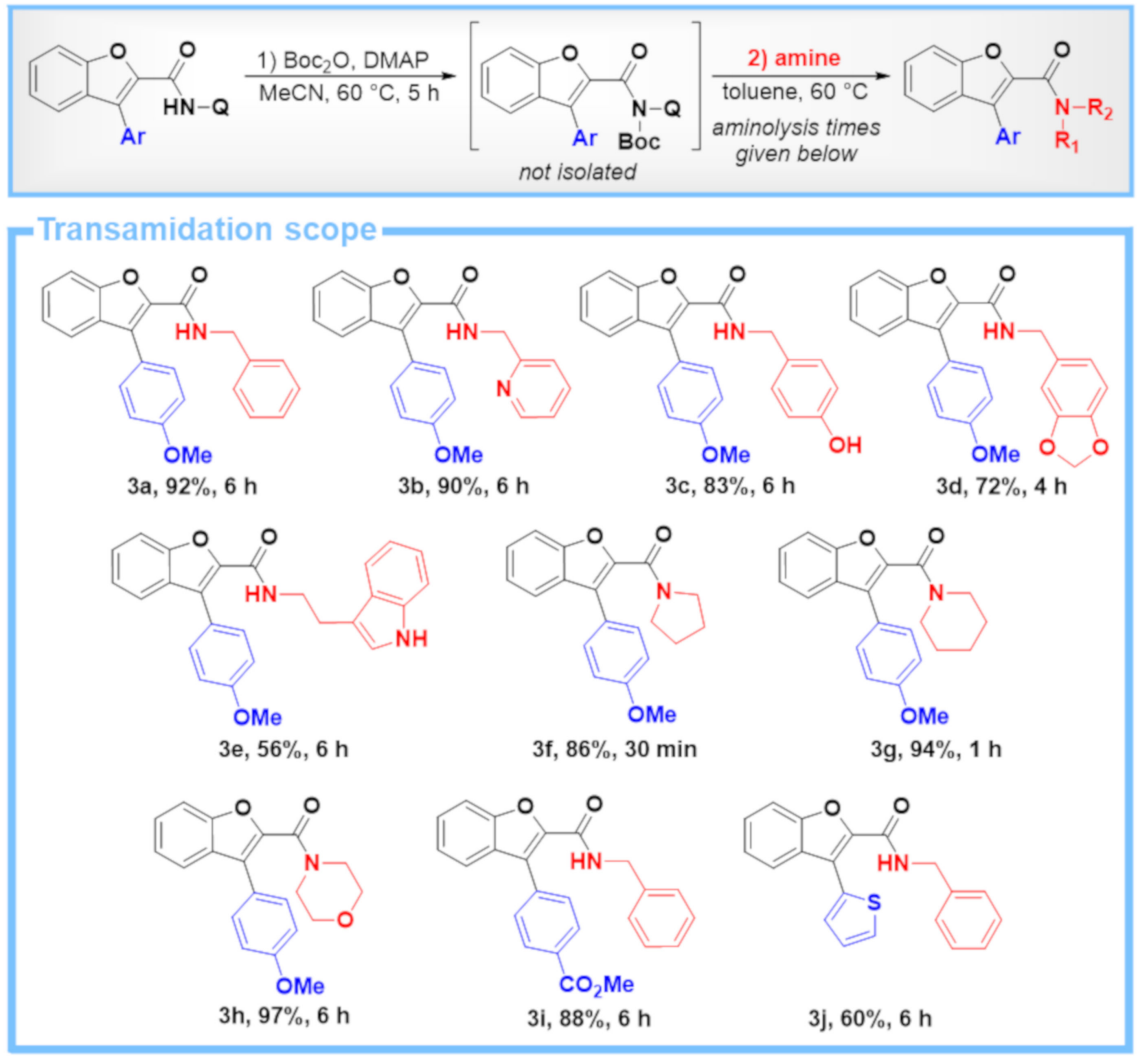
Scope of the two-step, one-pot transamidation procedure in terms of different amines and benzofuran substrates. Step 1: Boc_2_O (5 equiv.), DMAP (15 mol%), MeCN, 60 °C, 5 h. Step 2: Amine (1.5 equiv.), toluene, 60 °C, for the times given above. Yields refer to the isolated yield after both steps.

In this study, we were of course most interested in using such a simpler one-pot approach for the transamidation of our benzofuran products. To our delight, this desired one-pot strategy proved to be highly efficient for a number of transamidation reactions involving different benzofuran products and amine nucleophiles (Scheme 5). In these one-pot transamidation reactions, the benzofuran products were first dissolved in MeCN and subjected to Boc_2_O/DMAP at 60 °C for 5 h, after which the reaction mixtures were concentrated in vacuo. The crude reaction mixtures were then re-dissolved in toluene and the amine was added, after which they were allowed to stir at 60 °C for the times needed for the aminolysis reactions to be completed. Once finished, the reactions were all concentrated in vacuo and purified by column chromatography.

As can be seen from our survey of different amine nucleophiles in the transamidation of C–H arylation product **2a** (Ar = 4-MeO-Ph), this one-pot protocol works very well for a wide range primary and secondary amines. For example, when the transamidation was carried out with benzyl amine and an aminolysis time of 6 h, the product **3a** was obtained in an excellent yield of 92%. Very good results were also obtained with 2-picolylamine and 4-hydroxybenzylamine under the same conditions, and from these reactions products **3b** and **3c** could be isolated in 90% and 83% yield, respectively. The transamidation also worked fairly well with piperonylamine, and here product **3d** was afforded in 72% yield after 4 h of aminolysis. On the other hand, the transamidation with tryptamine was found to proceed less efficiently, but still product **3e** could be isolated in a synthetically useful yield of 56%.

Interestingly, the transamidations were found to be highly efficient when cyclic amines were used as the nucleophiles. For example, when using pyrrolidine, product **3f** could be isolated in a high yield of 86% after only 30 min of aminolysis. Excellent results were also obtained with the reactions involving piperidine and morpholine, which furnished the products **3g** and **3h** in 94% and 97% yield, respectively. Here, the aminolysis with piperidine was done within 1 h, while morpholine called for a longer aminolysis time of 6 h to reach completion.

In addition to surveying different amine nucleophiles, we also explored the transamidation of other C–H arylation products using benzyl amine as the nucleophile. One interesting feature of this transamidation procedure that was demonstrated in our earlier study, is the possibility of transamidating the 8-AQ amide moiety while leaving other electrophilic functionalities such as ester groups untouched [59]. Such electrophilic groups are usually not compatible with other 8-AQ removal conditions that involve hydrolysis under strongly acidic or basic conditions. In this study, the possibility to efficiently transamidate such sensitive C–H arylation products was again demonstrated with the reaction that gave benzofuran-2-carboxamide **3i** bearing a methyl ester group in a high yield of 88%. Moreover, the thiophene-containing product **2m** was also found to be compatible with the transamidation conditions, and from this reaction we were able to isolate benzofuran-2-carboxamide **3j** in 60% yield.

**Scheme 6:**
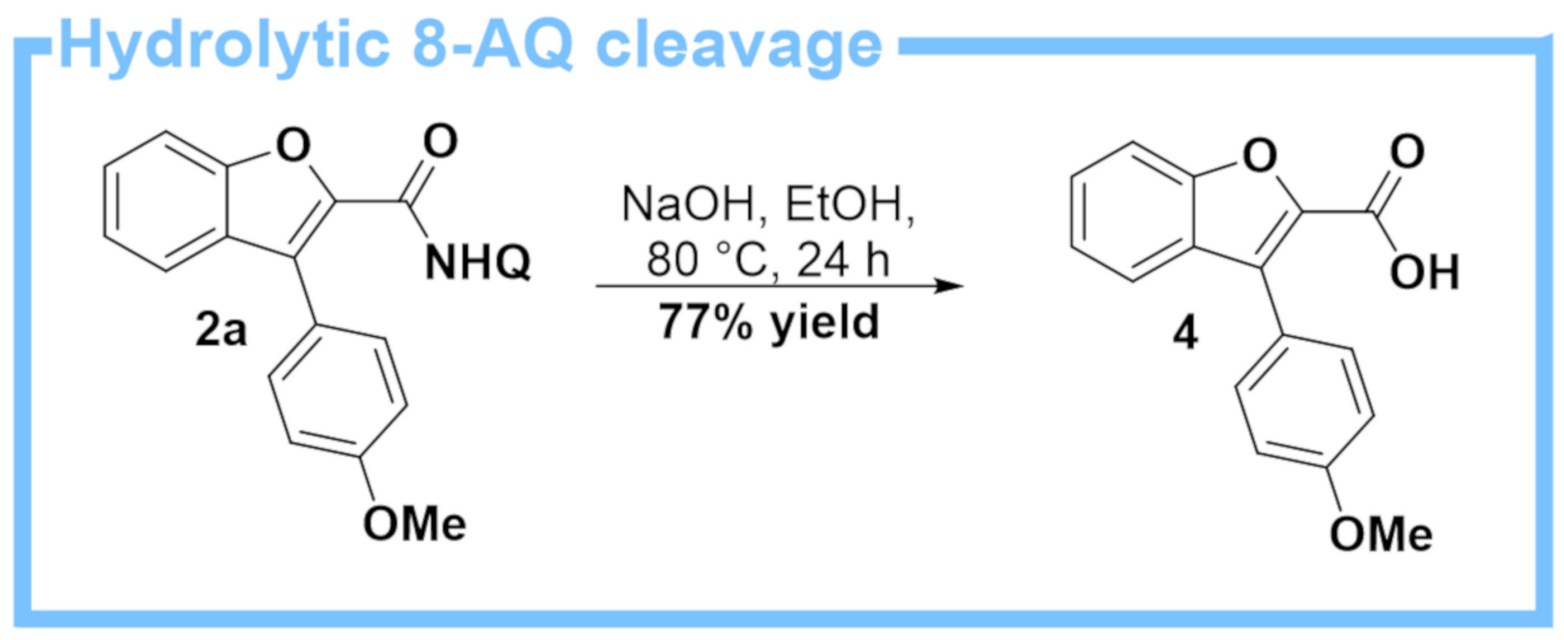
Synthesis of carboxylic acid **4** by hydrolytic removal of the 8-AQ auxiliary from **2a** with NaOH/EtOH.

If the corresponding carboxylic acids are desired instead, it is of course also possible to remove the 8-AQ auxiliary from the C–H arylation products using the conventional hydrolytic approach. As depicted in Scheme 6 with compound **2a** as the model substrate, hydrolysis to the carboxylic acid **4** can be achieved in 77% yield using NaOH in EtOH (Scheme 6). 

## 3. Discussion

In summary, we have outlined a modular synthetic strategy that can be used to prepare a diverse set of C3-substituted, benzofuran-2-carboxamide derivatives, from a simple benzofuran precursor in only three synthetic steps. This short synthetic route was enabled by the strategic combination of 8-AQ directed C–H arylation and transamidation chemistry. To our delight, the developed C–H arylation protocol was found to work well for a wide range of aryl iodides and benzofuran substrates, which allowed for the synthesis of a diverse set of C3-arylated benzofuran products. For the transamidation of the obtained C–H arylated benzofuran products, this could be achieved by a highly efficient two-step, one-pot protocol that enabled access to a number of C3-substituted, benzofuran-2-carboxamide derivatives in good to excellent yields. We expect that our developed synthetic route should be of great use when creating small molecule screening libraries based on densely functionalized benzofuran derivatives. 

## 4. Materials and Methods 

### 4.1. General Procedure for the C–H Arylation of Substrates **1a–e**

A reaction vial was charged with *N*-(quinolin-8-yl)benzofuran-2-carboxamides **1a–e** (1.0 equiv), aryl iodide (3.0 equiv), Pd(OAc)_2_ (5 to 10 mol%), NaOAc (1.0 equiv) and AgOAc (1.5 equiv) the solids were then suspended in CPME (0.5 M). The reactions were allowed to stir at 110 °C for the times given in Scheme 2 under inert atmosphere. Once complete, the crude reaction mixture was diluted with a small amount of EtOAc and filtered through a plug of silica. Two different purification methods were used depending on the solubility of the C–H arylation products. Those products that displayed good solubility and passed through the silica pad were purified by column chromatography. In those cases where the products were retained on the silica pad, the silica was collected and subjected to a Soxhlet extraction with DCM.

### 4.2. General Procedure C: Two-Step One-Pot Transamidation of C–H Arylation Products with Different Amines

#### 4.2.1. Boc Activation

To a solution of the C–H arylation product **2a**, **2k**, or **2m** (1.0 equiv) in MeCN (0.1 M) were added (Boc)_2_O (2.0 equiv) and DMAP (0.1 equiv). The reaction was stirred at 60 °C for 5 h, after which it was concentrated in vacuo. The crude product was used without further purification.

#### 4.2.2. Aminolysis

To the crude reaction mixture from step 1, toluene (0.5 M) and the amine (1.5 equiv) were added. The aminolysis reactions were carried out at 60 °C for 0.5 to 6 h. Once completed, the reaction mixture was concentrated under reduced pressure and purified by column chromatography to give products **3a–j**.

## Figures and Tables

**Table 1:**
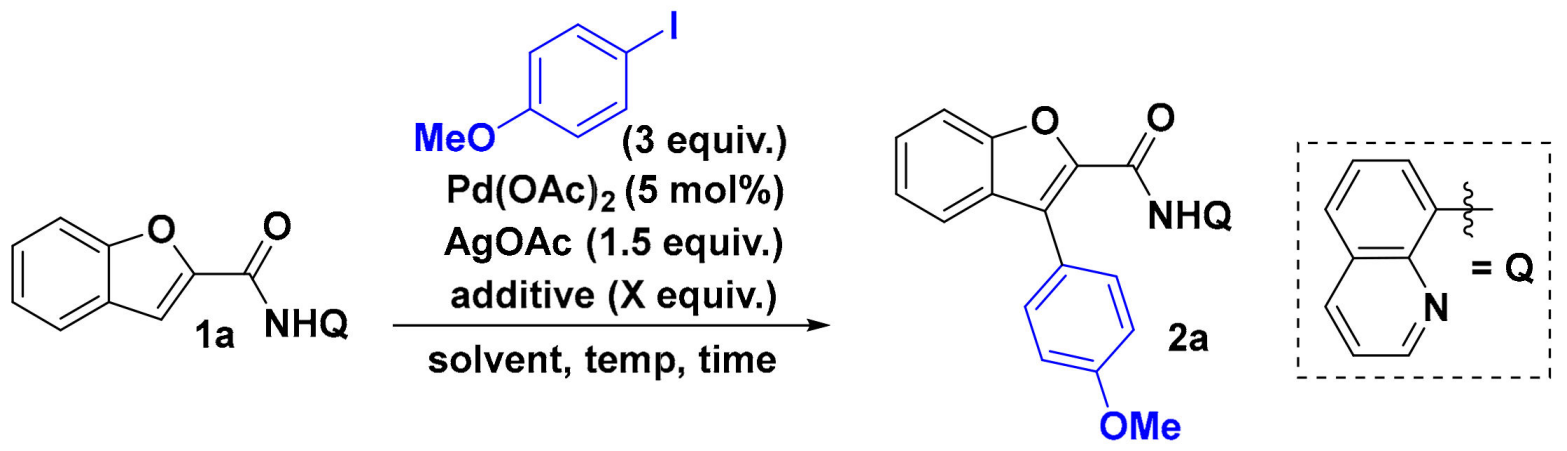
Optimization of the Pd-catalyzed C–H arylation of substrate **1a** with 4-iodoanisole ^[a]^.

Entry	Additive (equiv.)	Solvent	Temp (°C)	Time (h)	Yield (%)
1	none	toluene	110	7	46
2	none	toluene	110	16	65
3	none	toluene	120	7	30
4	(BnO)_2_PO_2_H (0.2)	toluene	110	7	28
5	PivOH (0.2)	toluene	110	7	61
6	PivOH (0.5)	toluene	110	7	48
7	PivOH (1.0)	toluene	110	7	17
8	NaOAc (0.5)	toluene	110	7	62
9	NaOAc (1.0)	toluene	110	7	78
10	NaOAc (2.0)	toluene	110	7	70
11	PivOH+NaOAc (0.2 + 1.0)	toluene	110	7	56
12	NaOAc (1.0)	*t*-amyl OH	110	7	91
13	NaOAc (1.0)	MeTHF	110	7	81
14	NaOAc (1.0)	CPME	110	7	93
15	NaOAc (1.0)	DCE	110	7	69
16	NaOAc (1.0)	MeCN	110	7	18
17 ^[b]^	NaOAc (1.0)	CPME	110	15	80
18 ^[b,c]^	NaOAc (1.0)	CPME	110	15	73

^[a]^ Reagents and conditions: Substrate **1a** (0.1 mmol, Q = 8-quinolinyl), 4-iodoanisole (3 equiv.), Pd(OAc)_2_ (5 mol%), AgOAc (1.5 equiv.), and the additive(s) were dissolved in solvent (0.5 M) and heated at the given temperature under inert atmosphere. Yields were determined by ^1^H-NMR using 1,3,5-trimethoxybenzene as a standard. ^[b]^ 2 equiv. of 4-iodoanisole were used instead. ^[c]^ Concentration = 1 M.

## Supplementary Materials

The following are available online. Table S1: Screening of Pd catalysts and Ag salts., Table S2: Screening of AgOAc loading.
