# Supplementary material for: Synthesis of Elaborate Benzofuran-2-Carboxamide Derivatives through a Combination of 8-Aminoquinoline Directed C–H Arylation and Transamidation Chemistry
Source: Molecules. 2020 Jan 15;25(2):361. doi: 10.3390/molecules25020361 (PMC7024369; doi:10.3390/molecules25020361)

Supporting Information for

Synthesis of elaborate benzofuran-2-carboxamide derivatives through a combination of 8-aminoquinoline directed C–H arylations and transamidations

Michael Oschmann,[†] Linus Johansson Holm,[†] Monireh Pourghasemi-Lati, Oscar Verho*

Department of Organic Chemistry, Arrhenius Laboratory, Stockholm University, SE-106 91 Stockholm, Sweden.

[†]These authors contributed equally to this work.

*To whom correspondence should be addressed. E-mail: oscar.verho@su.se

General experimental information **2**

General procedure A: Synthesis of the 8-AQ amide substrates 1a-e **2**

Synthesis of substrate **1a** from the acid chloride 2

Synthesis of substrates **1a-e** from the carboxylic acids 2

General procedure B: C-H arylation of substrates 1a-e **2**

General procedure C: Two-step-one pot transamidation of C-H arylation products with different amines **3**

Step 1: Boc activation 3

Step 2: Aminolysis 3

Characterization data and other experimental procedures **4**

Basic hydrolysis of amide (**2a**) to carboxylic acid (**4**) 22

NMR Spectra **23**

General experimental information

Unless otherwise noted, all reagents and reactants were used as received from commercial suppliers. All solvents were obtained from commercial suppliers and used without further drying or purification. All reactions were monitored by thin-layer chromatography (TLC) using E. Merck silica gel 60 F254 plates (TLC analysis), which were visualized by UV light (254 nm). Flash chromatography was performed using 15-45 µm silica gel cartridges (60 Å mesh) on a Teledyne Isco Combiflash Rf. SiliaSep SiO_2 cartridges used for these purifications were obtained from SiliCycle. ^{1}H and ^{13}C NMR spectra were recorded on Bruker Avance-II instruments at 400 MHz (H) and at 100 MHz (C), respectively. Chemical shifts (δ) are reported in ppm, using the residual solvent peak in $CDCl_3$ (δ(H)= 7.26 and δ(C)= 77.2 ppm) as internal standard, and coupling constants (*J*) are given in Hz. Data for ^{1}H-NMR are reported as follows: chemical shift, multiplicity (s = singlet, d = doublet, t = triplet, m = multiplet), coupling constants, and integration.

General procedure A: Synthesis of the 8-AQ amide substrates 1a-e

*Synthesis of substrate **1a** from the acid chloride*

To a solution of benzofuran-2-carbonyl chloride (1.0 equiv) in DCM (0.1 M), was added 8-aminoquinoline (8-AQ, 1.1 equiv) and NEt_3 (2 equiv). The resulting reaction mixture was allowed to stir at rt for 5 h, after which it was diluted with DCM and washed with H_2O (3x). The organic layer was dried over $MgSO_4$, filtered and concentrated under reduced pressure. Purification by column chromatography (0-2% MeOH in DCM) afforded the desired product.

*Synthesis of substrates **1a-e** from the carboxylic acids*

To a solution of the benzofuran-2-carboxylic acid substrate (1.0 equiv) in DCM (0.1 M), was added 8-AQ (1.5 equiv), HATU (1.7 equiv.) and DIPEA (1.9 equiv). The resulting reaction mixture was stirred at 0 °C for 20 min and then at rt for 24 h. Then, the reaction mixture was diluted with DCM and washed with H_2O (3x). The organic layer was dried over $MgSO_4$, filtered and concentrated under reduced pressure. Pure products were obtained following column chromatography (0-2% MeOH in DCM)

General procedure B: C-H arylation of substrates 1a-e

A reaction vial was charged with *N*-(quinolin-8-yl)benzofuran-2-carboxamides **1a-e** (1.0 equiv), aryl iodide (3.0 equiv), $Pd(OAc)_2$ (5-10 mol%), NaOAc (1.0 equiv) and AgOAc (1.5 equiv) then suspended in CPME (0.5 M), the atmosphere in the vial was flushed with argon. The reactions were allowed to stir at 110 °C for the times given in Scheme 2 under inert atmosphere. Once complete, the crude reaction mixture was diluted with a small amount of EtOAc and filtered through a plug of silica. Two different purification methods were used depending on the solubility of the C-H arylation products. Those products that displayed good solubility and passed through the silica pad were purified by column chromatography. In those cases where the products were retained on the silica pad, the silica was collected and subjected to a Soxhlet extraction with DCM.

General procedure C: Two-step-one pot transamidation of C-H arylation products with different amines

Step 1: Boc activation

To a solution of the C-H arylation product **2a, 2k** or **2m** (1.0 equiv) in MeCN (0.1 M) were added $(Boc)_2O$ (2.0 equiv) and DMAP (0.1 equiv). The reaction were stirred at 60 °C for 5 h, after which it was concentrated *in vacuo*. The crude product was used without further purification.

Step 2: Aminolysis

To the crude reaction mixture from step 1, toluene (0.5 M) and the amine (1.5 equiv) were added. The aminolysis reactions were carried out at 60 °C for 0.5–6 h. Once completed, the reaction mixture were concentrated under reduced pressure and purified by column chromatography to give products **3a–j**.

Supporting data

Table S 1. Screening of Pd catalysts and Ag salts.

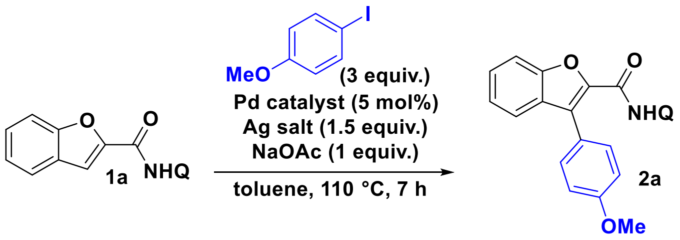

Entry	Pd cat.	Ag salt	Yield (%)
1	$Pd(OAc)_2$	AgOAc	78
2	$PdCl_2$	AgOAc	50
3	$Pd(TFA)_2$	AgOAc	66
4	$Pd(dba)_2$	AgOAc	61
5	-	AgOAc	<5
6	$Pd(OAc)_2$	Ag_2CO_3	49

Yields determined by ^{1}H-NMR against 1,3,5-trimethoxybenzene as internal standard.

Table S 2. Screening of AgOAc loading.

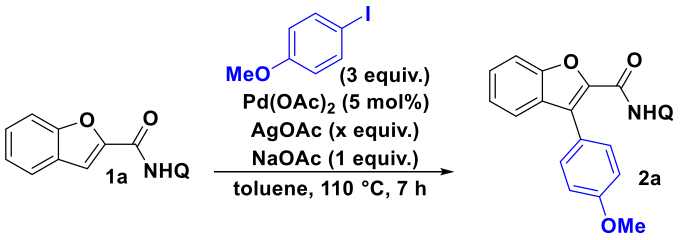

Entry	Pd cat.	Ag salt	Yield (%)
1	$Pd(OAc)_2$	-	5
2	$Pd(OAc)_2$	AgOAc (0.3 equiv.)	30
3	$Pd(OAc)_2$	AgOAc (1.0 equiv.)	55
4	$Pd(OAc)_2$	AgOAc (1.5 equiv.)	78

Yields determined by ^{1}H-NMR against 1,3,5-trimethoxybenzene as internal standard.

Characterization data and other experimental procedures

N-(quinolin-8-yl)benzofuran-2-carboxamide (**1a**)

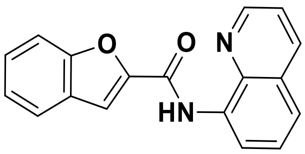

This compound was synthesized according to both General procedure A. Purified by column chromatography (0-2% MeOH in DCM).

From benzofuran-2-carboxylic acid: Scale: 1.5 mmol. Isolated yield 73% (0.31 g)
From benzofuran-2-carbonyl chloride: Scale: 1.8 mmol. Isolated yield: 97% (0.52 g).
^{1}H NMR (400 MHz, $CDCl_3$) δ: 11.01 (s, 1H), 8.93 (dt, *J* = 6.5, 1.8 Hz, 2H), 8.18 (dt, *J* = 8.3, 1.4 Hz, 1H), 7.70 (tdd, *J* = 9.3, 2.0, 0.9 Hz, 2H), 7.66 – 7.63 (m, 1H), 7.62 – 7.53 (m, 2H), 7.53 – 7.43 (m, 2H), 7.36 – 7.30 (m, 1H).

^{13}C NMR (101 MHz, $CDCl_3$) δ: 157.0, 155.2, 149.3, 148.7, 138.8, 136.5, 134.2, 128.1, 127.9, 127.5, 127.2, 123.9, 122.8, 122.3, 121.9, 117.1, 112.3, 111.3.

HRMS-ESI (*m/z*): $[M + Na]^+$ calcd for $C_{18}H_{12}N_2O_2$ 311.0791; found, 311.0779.

7-Methoxy-*N*-(quinolin-8-yl)benzofuran-2-carboxamide (**1b**)

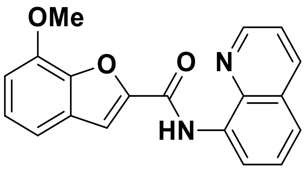

This compound was synthesized according to General procedure A. Scale: 0.8 mmol. Isolated yield: 94% (233 mg). Purified by column chromatography (0-2% MeOH in DCM).

^{1}**H NMR** (400 MHz, $CDCl_3$) δ: 11.03 (s, 1H), 8.96 – 8.87 (m, 2H), 8.16 (dd, *J* = 8.3, 1.7 Hz, 1H), 7.63 (s, 1H), 7.61 – 7.52 (m, 2H), 7.46 (dd, *J* = 8.3, 4.2 Hz, 1H), 7.29 (dd, *J* = 7.9, 1.2 Hz, 1H), 7.22 (t, *J* = 7.8 Hz, 1H), 6.94 (dd, *J* = 7.8, 1.1 Hz, 1H), 4.11 (s, 3H).

^{13}**C NMR** (101 MHz, $CDCl_3$) δ: 156.9, 149.3, 148.7, 145.9, 144.7, 138.8, 136.3, 134.1, 129.5, 128.1, 127.4, 124.6, 122.3, 121.8, 117.1, 114.8, 111.6, 109.2, 56.4.

HRMS-ESI (*m/z*): $[M + Na]^+$ calcd for $C_{19}H_{14}N_2O_3$ 341.0897; found, 341.0893.

5-Methoxy-*N*-(quinolin-8-yl)benzofuran-2-carboxamide (**1c**)

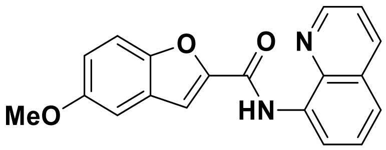

This compound was synthesized according to General procedure A. Scale: 0.7 mmol. Isolated yield: 94% (210 mg). Purified by column chromatography (0-2% MeOH in DCM).

^{1}H NMR (400 MHz, $CDCl_3$) δ: 10.96 (s, 1H), 8.98 – 8.87 (m, 2H), 8.17 (dd, *J* = 8.3, 1.7 Hz, 1H), 7.63 – 7.51 (m, 4H), 7.48 (dd, *J* = 8.2, 4.2 Hz, 1H), 7.15 – 7.02 (m, 2H), 3.86 (s, 3H).

^{13}C NMR (101 MHz, $CDCl_3$) δ: 157.0, 156.6, 150.2, 149.9, 148.6, 138.8, 136.4, 134.2, 128.4, 128.1, 127.5, 122.2, 121.9, 117.0, 116.9, 112.9, 111.4, 104.0, 56.0.

HRMS-ESI (*m/z*): $[M + Na]^+$ calcd for $C_{19}H_{14}N_2O_3$ 341.0897; found, 341.0894.

5-Chloro-*N*-(quinolin-8-yl)benzofuran-2-carboxamide (**1d**)

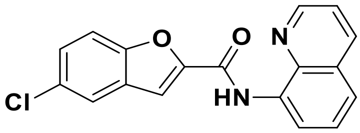

This compound was synthesized according to General procedure A. Scale: 0.8 mmol. Isolated yield: 99% (251 mg) Purified by column chromatography (0-2% MeOH in DCM).

^{1}H NMR (400 MHz, $CDCl_3$) δ: 11.02 (s, 1H), 8.95 (dd, *J* = 4.2, 1.7 Hz, 1H), 8.92 (dd, *J* = 6.0, 2.9 Hz, 1H), 8.22 (dd, *J* = 8.3, 1.7 Hz, 1H), 7.71 (dd, *J* = 2.2, 0.5 Hz, 1H), 7.66 – 7.58 (m, 4H), 7.53 (dd, *J* = 8.3, 4.2 Hz, 1H), 7.44 (dd, *J* = 8.8, 2.2 Hz, 1H).

^{13}C NMR (101 MHz, $CDCl_3$) δ: 156.6, 153.5, 150.6, 148.8, 138.9, 136.6, 134.0, 129.6, 129.2, 128.2, 127.6, 127.5, 122.6, 122.3, 122.0, 117.3, 113.5, 110.7.

HRMS-ESI (*m/z*): $[M + Na]^+$ calcd for $C_{18}H_{11}ClN_2O_2$ 345.0401; found, 345.0386.

N-Phenylbenzofuran-2-carboxamide (**1e**)

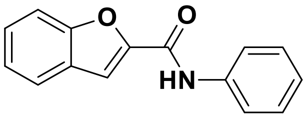

Following General procedure A starting from benzofuran-2-carboxylic acid and using aniline instead of 8-AQ. Scale: 0.1 mmol. Isolated yield: 77% (36.7 mg, 0.14 mmol). Purified by acid/base extraction.

^{1}H NMR (400 MHz, $CDCl_3$) δ: 8.38 (s, 1H), 7.77 – 7.67 (m, 3H), 7.62 – 7.53 (m, 2H), 7.45 (ddd, *J* = 8.4, 7.2, 1.4 Hz, 1H), 7.43 – 7.36 (m, 2H), 7.32 (ddd, *J* = 8.1, 7.2, 1.0 Hz, 1H), 7.22 – 7.14 (m, 1H).

^{13}C NMR (101 MHz, $CDCl_3$) δ: 156.7, 154.9, 148.6, 137.3, 129.3, 127.8, 127.4, 124.9, 124.0, 123.0, 120.2, 111.9, 111.6.

HRMS-ESI (*m/z*): $[M + Na]^+$ calcd for $C_{15}H_{11}NO_2$ 260.0682; found, 260.0682

3-(4-Methoxyphenyl)-*N*-(quinolin-8-yl)benzofuran-2-carboxamide (**2a**)

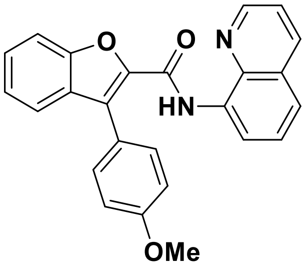

This compound was synthesized according to General procedure B using 5 mol% $Pd(OAc)_2$. Purified by column chromatography (0-2% MeOH in DCM).

Regular scale: 0.15 mmol (7 h reaction time). Isolated yield: 86% (51.0 mg).
Larger scale: 0.9 mmol (24 h reaction time). Isolated yield: 92% (327 mg).

^{1}H NMR (400 MHz, $CDCl_3$) δ: 10.97 (s, 1H), 8.91 (dd, *J* = 5.0, 4.0 Hz, 1H), 8.84 (dd, *J* = 4.2, 1.7 Hz, 1H), 8.18 (dd, *J* = 8.3, 1.7 Hz, 1H), 7.73 (dt, *J* = 8.4, 0.8 Hz, 1H), 7.73 – 7.62 (m, 3H), 7.57 – 7.44 (m, 4H), 7.34 (ddd, *J* = 8.0, 7.2, 0.9 Hz, 1H), 7.13 – 7.04 (m, 2H), 3.90 (s, 3H).

^{13}C NMR (101 MHz, $CDCl_3$) δ: 160.0, 157.6, 154.0, 148.4, 142.7, 138.9, 136.4, 134.5, 131.6, 129.3, 128.1, 127.6, 127.5, 126.8, 123.8, 122.8, 122.1, 122.0, 121.8, 117.2, 114.2, 112.3, 55.5.

HRMS-ESI (*m/z*): $[M + Na]^+$ calcd for $C_{25}H_{18}N_2O_3$ 417.1210; found, 417.1191.

3-(3,5-Dimethylphenyl)-*N*-(quinolin-8-yl)benzofuran-2-carboxamide (**2b**)

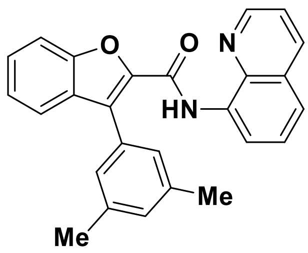

This compound was synthesized according to General procedure B using 5 mol% $Pd(OAc)_2$ for 14 h. Scale: 0.15 mmol. Isolated yield: 76% (45.0 mg). Purified by column chromatography (0-30% EtOAc in pentane).

^{1}H NMR (400 MHz, $CDCl_3$) δ: 10.86 (s, 1H), 8.91 (dd, *J* = 6.7, 2.3 Hz, 1H), 8.79 – 8.73 (m, 1H), 8.16 (dd, *J* = 8.3, 1.7 Hz, 1H), 7.72 (dt, *J* = 8.3, 0.8 Hz, 1H), 7.61 (ddd, *J* = 7.9, 1.3, 0.7 Hz, 1H), 7.59 – 7.46 (m, 3H), 7.46 (dd, *J* = 8.2, 4.2 Hz, 1H), 7.37 – 7.28 (m, 3H), 7.15 (dt, *J* = 1.7, 0.9 Hz, 1H), 2.41 (q, *J* = 0.7 Hz, 6H).

^{13}C NMR (101 MHz, $CDCl_3$) δ: 157.5, 154.0, 148.3, 143.1, 139.0, 138.3, 136.3, 134.6, 130.5, 130.4, 129.5, 128.1, 128.0, 127.55, 127.52, 126.9, 123.8, 122.2, 121.9, 121.7, 117.2, 112.3, 21.6.

HRMS-ESI (*m/z*): $[M + Na]^+$ calcd for $C_{26}H_{20}N_2O_2$ 415.1417; found, 415.1404.

N-(quinolin-8-yl)-3-(p-tolyl)benzofuran-2-carboxamide (**2c**)

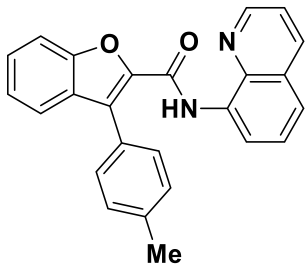

This compound was synthesized according to General procedure B using 5 mol% $Pd(OAc)_2$ for 14 h. Scale: 0.15 mmol. Isolated yield: 88% (50.0 mg). Purified by column chromatography (40-100% DCM in pentane).

^{1}H NMR (400 MHz, $CDCl_3$) δ: 10.96 (s, 1H), 8.91 (dd, *J* = 5.5, 3.5 Hz, 1H), 8.83 (dd, *J* = 4.2, 1.7 Hz, 1H), 8.16 (dd, *J* = 8.3, 1.7 Hz, 1H), 7.73 (dt, *J* = 8.3, 0.8 Hz, 1H), 7.67 – 7.61 (m, 3H), 7.56 – 7.49 (m, 3H), 7.47 (dd, *J* = 8.3, 4.2 Hz, 1H), 7.39 – 7.31 (m, 3H), 2.48 (s, 3H).

^{13}C NMR (101 MHz, $CDCl_3$) δ: 157.5, 154.0, 148.4, 142.8, 138.9, 138.5, 136.4, 134.5, 130.2, 129.4, 129.3, 128.1, 127.7, 127.6, 127.5, 127.0, 123.8, 122.1, 122.0, 121.8, 117.2, 112.3, 21.6.

HRMS-ESI (*m/z*): $[M + Na]^+$ calcd for $C_{25}H_{18}N_2O_2$ 401.1260; found, 401.1242.

3-Phenyl-*N*-(quinolin-8-yl)benzofuran-2-carboxamide (**2d**)

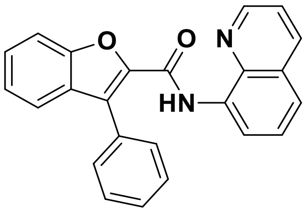

This compound was synthesized according to General procedure B using 5 mol% $Pd(OAc)_2$ for 16 h. Scale: 0.15 mmol. Isolated yield: 84% (46.0 mg). Purified by column chromatography (40-100% DCM in pentane).

^{1}H NMR (400 MHz, $CDCl_3$) δ: 10.97 (s, 1H), 8.90 (p, *J* = 4.4 Hz, 1H), 8.83 (dd, *J* = 4.2, 1.7 Hz, 1H), 8.17 (dd, *J* = 8.3, 1.7 Hz, 1H), 7.77 – 7.71 (m, 3H), 7.65 (ddd, *J* = 7.9, 1.3, 0.7 Hz, 1H), 7.60 – 7.44 (m, 7H), 7.35 (ddd, *J* = 8.0, 7.2, 0.9 Hz, 1H).

^{13}C NMR (101 MHz, $CDCl_3$) δ: 157.5, 154.0, 148.5, 142.9, 138.9, 136.4, 134.4, 130.7, 130.3, 129.2, 128.7, 128.6, 128.1, 127.7, 127.5, 126.9, 123.9, 122.09, 122.07, 121.8, 117.2, 112.3.

HRMS-ESI (*m/z*): $[M + Na]^+$ calcd for $C_{24}H_{16}N_2O_2$ 387.1104; found, 387.1096.

3-(Naphthalen-2-yl)-*N*-(quinolin-8-yl)benzofuran-2-carboxamide (**2e**)

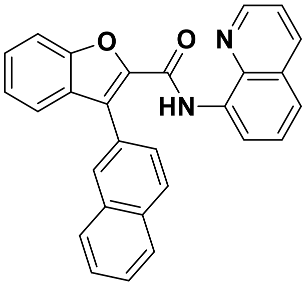

This compound was synthesized according to General procedure B using 5 mol% $Pd(OAc)_2$ for 24 h. Scale: 0.15 mmol. Isolated yield: 48% (30.0 mg). Purified by Soxhlet extraction.

^{1}H NMR (400 MHz, $CDCl_3$) δ: 10.90 (s, 1H), 8.89 (dd, *J* = 6.4, 2.6 Hz, 1H), 8.33 (dd, *J* = 4.2, 1.7 Hz, 1H), 8.22 – 8.18 (m, 1H), 8.11 (dd, *J* = 8.2, 1.7 Hz, 1H), 8.01 (d, *J* = 8.4 Hz, 1H), 7.98 – 7.90 (m, 2H), 7.81 (dd, *J* = 8.4, 1.7 Hz, 1H), 7.77 (d, *J* = 8.4 Hz, 1H), 7.69 – 7.64 (m, 1H), 7.61 – 7.48 (m, 5H), 7.40 – 7.33 (m, 2H).

^{13}C NMR (101 MHz, $CDCl_3$) δ: 157.4, 154.1, 152.1, 148.3, 143.3, 138.8, 136.3, 134.4, 133.6, 133.5, 129.6, 129.4, 128.5, 128.4, 128.24, 128.18, 128.03, 127.98, 127.7, 127.5, 126.7, 126.4, 124.0, 122.1, 122.0, 121.7, 117.1, 112.4.

HRMS-ESI (*m/z*): $[M + Na]^+$ calcd for $C_{28}H_{18}N_2O_2$ 437.1260; found, 437.1249.

3-(3-Fluorophenyl)-*N*-(quinolin-8-yl)benzofuran-2-carboxamide (**2f**)

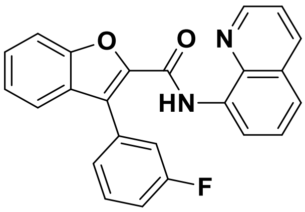

This compound was synthesized according to General procedure B using 5 mol% $Pd(OAc)_2$ for 16 h. Scale: 0.15 mmol. Isolated yield: 56% (32.0 mg). Purified by column chromatography (40-100% DCM in pentane).

^{1}H NMR (400 MHz, $CDCl_3$) δ: 11.00 (s, 1H), 8.91 – 8.85 (m, 2H), 8.19 (dd, *J* = 8.3, 1.7 Hz, 1H), 7.76 (dt, *J* = 8.3, 0.9 Hz, 1H), 7.63 (ddd, *J* = 7.9, 1.3, 0.7 Hz, 1H), 7.59 – 7.48 (m, 6H), 7.48 – 7.43 (m, 1H), 7.36 (ddd, *J* = 8.0, 7.1, 0.9 Hz, 1H), 7.19 (ddt, *J* = 8.7, 6.6, 2.6 Hz, 1H).

^{13}C NMR (101 MHz, $CDCl_3$) δ: 162.9 (d, *J* = 246 Hz), 157.2, 153.9, 148.5, 143.1, 138.9, 136.5, 134.3, 132.9 (d, *J* = 8.4 Hz), 130.1 (d, *J* = 8.4 Hz), 128.9, 128.2, 127.8, 127.5, 126.2 (d, *J* = 3.0 Hz), 125.7 (d, *J* = 2.3 Hz), 124.1, 122.2, 121.9, 121.8, 117.4 (d, *J* = 22.3 Hz), 117.2, 115.5 (d, *J* = 21.0 Hz), 112.4.

^{19}F NMR (377 MHz, $CDCl_3$) δ: -112.9.

HRMS-ESI (*m/z*): $[M + Na]^+$ calcd for $C_{24}H_{15}FN_2O_2$ 405.1010; found, 405.1003.

3-(4-Chlorophenyl)-*N*-(quinolin-8-yl)benzofuran-2-carboxamide (**2g**)

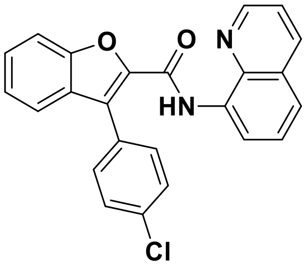

This compound was synthesized according to General procedure B using 5 mol% $Pd(OAc)_2$ for 16 h. Scale: 0.15 mmol. Isolated yield: 59% (35.0 mg). Purified by column chromatography (0-20% EtOAc in pentane).

^{1}H NMR (400 MHz, $CDCl_3$) δ: δ 10.99 (s, 1H), 8.90 – 8.85 (m, 2H), 8.18 (dd, *J* = 8.3, 1.7 Hz, 1H), 7.75 (dt, *J* = 8.4, 0.8 Hz, 1H), 7.70 – 7.65 (m, 2H), 7.65 – 7.57 (m, 1H), 7.58 – 7.45 (m, 6H), 7.36 (ddd, *J* = 8.0, 7.2, 0.9 Hz, 1H).

^{13}C NMR (101 MHz, $CDCl_3$) δ: 157.3, 154.0, 148.6, 143.0, 138.9, 136.5, 134.7, 134.3, 131.7, 129.2, 128.92, 128.89, 128.2, 127.9, 127.5, 125.8, 124.1, 122.2, 121.9, 121.8, 117.2, 112.5.

HRMS-ESI (*m/z*): $[M + Na]^+$ calcd for $C_{24}H_{15}ClN_2O_2$ 421.0714; found, 421.0693.

3-(4-Bromophenyl)-*N*-(quinolin-8-yl)benzofuran-2-carboxamide (**2h**)

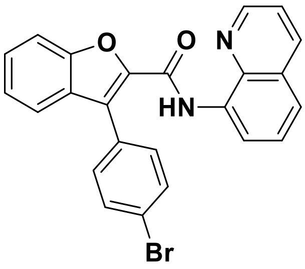

This compound was synthesized according to General procedure B using 5 mol% $Pd(OAc)_2$ for 24 h. Scale: 0.15 mmol. Isolated yield: 78% (52.0 mg). Purified by Soxhlet extraction.

^{1}H NMR (400 MHz, $CDCl_3$) δ: 10.99 (s, 1H), 8.92 – 8.83 (m, 2H), 8.18 (dd, *J* = 8.3, 1.7 Hz, 1H), 7.75 (dt, *J* = 8.4, 0.9 Hz, 1H), 7.70 – 7.66 (m, 2H), 7.64 – 7.58 (m, 3H), 7.57 – 7.51 (m, 3H), 7.49 (dd, *J* = 8.3, 4.2 Hz, 1H), 7.36 (ddd, *J* = 8.1, 7.2, 0.9 Hz, 1H).

^{13}C NMR (101 MHz, $CDCl_3$) δ: 157.3, 153.9, 148.6, 142.9, 138.9, 136.4, 134.3, 132.0, 131.8, 129.7, 128.8, 128.1, 127.8, 127.5, 125.8, 124.1, 122.9, 122.2, 121.9, 121.7, 117.2, 112.4.

HRMS-ESI (*m/z*): $[M + Na]^+$ calcd for $C_{24}H_{15}(Br^{79})N_2O_2$ 465.0209; found, 465.0192. For $C_{24}H_{15}(Br^{81})N_2O_2$ 467.0190; found, 467.0190.

3-(4-Iodophenyl)-*N*-(quinolin-8-yl)benzofuran-2-carboxamide (**2i**)

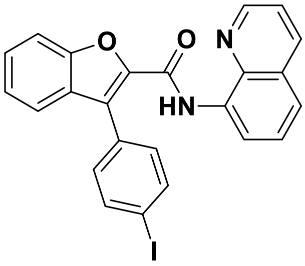

This compound was synthesized according to General procedure B using 5 mol% $Pd(OAc)_2$ for 24 h. Scale: 0.15 mmol. Isolated yield: 67% (49.0 mg). Purified by Soxhlet extraction.

^{1}H NMR (400 MHz, $CDCl_3$) δ: 10.99 (s, 1H), 8.88 (dt, *J* = 5.2, 3.8 Hz, 2H), 8.18 (dd, *J* = 8.3, 1.7 Hz, 1H), 7.93 – 7.84 (m, 2H), 7.75 (dd, *J* = 8.4, 0.9 Hz, 1H), 7.60 (dt, *J* = 7.9, 1.0 Hz, 1H), 7.58 – 7.45 (m, 6H), 7.36 (ddd, *J* = 8.1, 7.2, 1.0 Hz, 1H).

^{13}C NMR (101 MHz, $CDCl_3$) δ: 157.3, 154.0, 148.6, 142.9, 138.9, 137.8, 136.5, 134.3, 132.1, 130.3, 128.7, 128.2, 127.9, 127.5, 125.9, 124.1, 122.2, 121.9, 121.8, 117.2, 112.4, 94.8.

HRMS-ESI (*m/z*): $[M + Na]^+$ calcd for $C_{24}H_{15}IN_2O_2$ 513.0070; found, 513.0047.

3-(4-Acetylphenyl)-*N*-(quinolin-8-yl)benzofuran-2-carboxamide (**2j**)

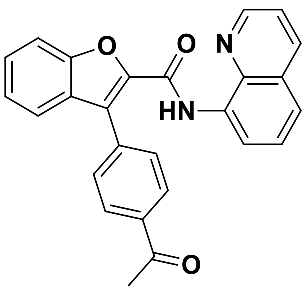

This compound was synthesized according to General procedure B using 5 mol% $Pd(OAc)_2$ for 16 h. Scale: 0.15 mmol. Isolated yield: 74% (45.0 mg). Purified by column chromatography (50-100% DCM in pentane).

^{1}H NMR (400 MHz, $CDCl_3$) δ: 11.07 (s, 1H), 8.91 – 8.85 (m, 2H), 8.20 (dd, *J* = 8.3, 1.7 Hz, 1H), 8.16 – 8.12 (m, 2H), 7.88 – 7.82 (m, 2H), 7.77 (dt, *J* = 8.4, 0.9 Hz, 1H), 7.63 (ddd, *J* = 7.9, 1.3, 0.7 Hz, 1H), 7.58 – 7.53 (m, 3H), 7.51 (dd, *J* = 8.2, 4.2 Hz, 1H), 7.37 (ddd, *J* = 8.1, 7.2, 0.9 Hz, 1H), 2.69 (s, 3H).

^{13}C NMR (101 MHz, $CDCl_3$) δ: 197.9, 157.2, 154.0, 148.6, 143.3, 139.0, 137.0, 136.5, 135.9, 134.3, 130.7, 128.8, 128.5, 128.2, 127.9, 127.5, 126.1, 124.3, 122.3, 121.9, 121.8, 117.3, 112.5, 26.9.

HRMS-ESI (*m/z*): $[M + Na]^+$ calcd for $C_{26}H_{18}N_2O_3$ 429.1210; found, 429.1202.

Methyl 4-(2-(quinolin-8-ylcarbamoyl)benzofuran-3-yl)benzoate (**2k**)

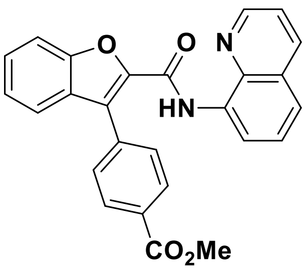

This compound was synthesized according to General procedure B using 5 mol% $Pd(OAc)_2$ for 16 h. Scale: 0.15 mmol. Isolated yield: 72% (45.0 mg). Purified by column chromatography (75-100% DCM in pentane).

^{1}H NMR (400 MHz, $CDCl_3$) δ: 11.02 (s, 1H), 8.89 – 8.84 (m, 2H), 8.26 – 8.18 (m, 2H), 8.18 (dd, *J* = 8.3, 1.7 Hz, 1H), 7.86 – 7.78 (m, 2H), 7.78 – 7.73 (m, 1H), 7.61 (ddd, *J* = 7.9, 1.3, 0.7 Hz, 1H), 7.58 – 7.51 (m, 3H), 7.49 (dd, *J* = 8.2, 4.2 Hz, 1H), 7.36 (ddd, *J* = 8.0, 7.2, 0.9 Hz, 1H), 3.98 (s, 3H).

^{13}C NMR (101 MHz, $CDCl_3$) δ: 167.0, 157.2, 154.0, 148.6, 143.2, 138.9, 136.5, 135.7, 134.3, 130.4, 130.2, 129.8, 128.8, 128.2, 127.9, 127.5, 126.0, 124.2, 122.3, 121.9, 121.8, 117.3, 112.5, 52.3.

HRMS-ESI (*m/z*): $[M + Na]^+$ calcd for $C_{26}H_{18}N_2O_4$ 445.1159; found, 445.1138.

3-(4-Nitrophenyl)-*N*-(quinolin-8-yl)benzofuran-2-carboxamide (**2l**)

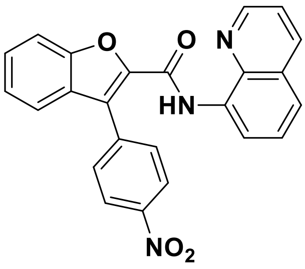

This compound was synthesized according to General procedure B using 5 mol% $Pd(OAc)_2$ for 16 h. Scale: 0.15 mmol. Isolated yield: 31% (19.0 mg). Purified by column chromatography (40-100% DCM in pentane).

^{1}H NMR (400 MHz, $CDCl_3$) δ: 11.11 (s, 1H), 8.92 (dd, *J* = 4.2, 1.7 Hz, 1H), 8.84 (dd, *J* = 7.0, 2.0 Hz, 1H), 8.42 – 8.37 (m, 2H), 8.21 (dd, *J* = 8.3, 1.7 Hz, 1H), 7.95 – 7.89 (m, 2H), 7.79 (d, *J* = 8.5 Hz, 1H), 7.63 – 7.48 (m, 5H), 7.43 – 7.36 (m, 1H).

^{13}C NMR (101 MHz, $CDCl_3$) δ: 157.0, 154.0, 148.7, 147.9, 143.5, 138.9, 138.0, 136.6, 134.1, 131.4, 128.3, 128.22, 128.21, 127.5, 125.0, 124.5, 123.7, 122.5, 122.0, 121.4, 117.3, 112.7.

HRMS-ESI (*m/z*): $[M + Na]^+$ calcd for $C_{24}H_{15}N_3O_4$ 432.0955; found, 432.0940.

N-(quinolin-8-yl)-3-(thiophen-2-yl)benzofuran-2-carboxamide (**2m**)

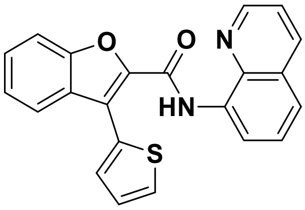

This compound was synthesized according to General procedure B using 5 mol% $Pd(OAc)_2$ for 16 h. Scale: 0.15 mmol. Isolated yield: 86% (48.0 mg). Purified by column chromatography (50-100% DCM in pentane).

^{1}H NMR (400 MHz, $CDCl_3$) δ: 11.06 (s, 1H), 8.95 (dd, *J* = 6.2, 2.8 Hz, 1H), 8.89 (dd, *J* = 4.2, 1.7 Hz, 1H), 8.19 (dd, *J* = 8.3, 1.7 Hz, 1H), 7.93 (d, *J* = 7.9 Hz, 1H), 7.77 (dd, *J* = 3.6, 1.2 Hz, 1H), 7.73 (d, *J* = 8.4 Hz, 1H), 7.60 – 7.51 (m, 4H), 7.49 (dd, *J* = 8.2, 4.2 Hz, 1H), 7.39 (ddd, *J* = 8.1, 7.1, 1.0 Hz, 1H), 7.25 (dd, *J* = 5.1, 3.6 Hz, 1H).

^{13}C NMR (101 MHz, $CDCl_3$) δ: 157.3, 153.8, 148.5, 143.0, 138.9, 136.4, 134.4, 130.9, 130.1, 128.7, 128.2, 127.9, 127.52, 127.47, 127.46, 124.1, 122.6, 122.2, 121.8, 120.2, 117.2, 112.4.

HRMS-ESI (*m/z*): $[M + Na]^+$ calcd for $C_{22}H_{14}N_2O_2S$ 393.0668; found, 393.0650.

Ethyl 4-oxo-6-(2-(quinolin-8-ylcarbamoyl)benzofuran-3-yl)-4*H*-chromene-2-carboxylate (**2n**)

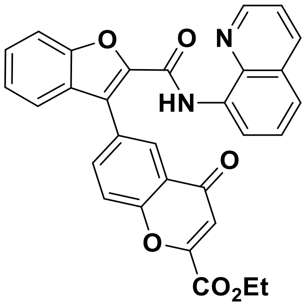

This compound was synthesized according to General procedure B using 10 mol% $Pd(OAc)_2$ for 24 h.. Scale: 0.15 mmol. Isolated yield: 94% (72.0 mg). Purified by Soxhlet extraction.

^{1}H NMR (400 MHz, $CDCl_3$) δ: 11.10 (s, 1H), 8.91 (dd, *J* = 4.2, 1.7 Hz, 1H), 8.83 (dd, *J* = 6.8, 2.2 Hz, 1H), 8.52 (d, *J* = 2.2 Hz, 1H), 8.20 (ddd, *J* = 8.7, 7.2, 2.0 Hz, 2H), 7.78 (dd, *J* = 8.5, 3.9 Hz, 2H), 7.68 – 7.63 (m, 1H), 7.60 – 7.48 (m, 4H), 7.38 (ddd, *J* = 8.0, 7.2, 0.9 Hz, 1H), 7.18 (s, 1H), 4.50 (q, *J* = 7.1 Hz, 2H), 1.47 (t, *J* = 7.1 Hz, 3H).

^{13}C NMR (101 MHz, $CDCl_3$) δ: 178.3, 160.7, 157.2, 156.0, 153.9, 152.4, 148.7, 143.2, 138.9, 137.6, 136.5, 134.1, 129.1, 128.5, 128.2, 128.1, 127.4, 126.9, 125.3, 124.44, 124.37, 122.4, 121.9, 121.7, 119.0, 117.3, 115.1, 112.5, 63.2, 14.3.

HRMS-ESI (*m/z*): $[M + Na]^+$ calcd for $C_{30}H_{20}N_2O_6$ 527.1214; found, 527.1200.

5-Methoxy-3-(4-methoxyphenyl)-*N*-(quinolin-8-yl)benzofuran-2-carboxamide (**2o**)

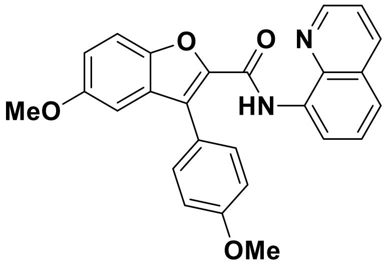

This compound was synthesized according to General procedure B using 10 mol% $Pd(OAc)_2$ for 24 h. Scale: 0.15 mmol. Isolated yield: 80% (50.0 mg). Purified by column chromatography (50-100% DCM in pentane).

^{1}H NMR (400 MHz, $CDCl_3$) δ: 10.91 (s, 1H), 8.89 (dd, *J* = 5.8, 3.2 Hz, 1H), 8.82 (dd, *J* = 4.2, 1.7 Hz, 1H), 8.15 (dd, *J* = 8.3, 1.7 Hz, 1H), 7.71 – 7.63 (m, 2H), 7.61 (d, *J* = 9.0 Hz, 1H), 7.56 – 7.49 (m, 2H), 7.46 (dd, *J* = 8.2, 4.2 Hz, 1H), 7.16 – 7.05 (m, 3H), 7.01 (d, *J* = 2.6 Hz, 1H), 3.90 (s, 3H), 3.83 (s, 3H).

^{13}C NMR (101 MHz, $CDCl_3$) δ: 159.8, 157.4, 156.7, 148.8, 148.3, 143.3, 138.7, 136.2, 134.3, 131.4, 129.7, 128.0, 127.3, 126.6, 122.8, 121.8, 121.6, 117.3, 117.0, 114.1, 112.8, 103.0, 55.9, 55.3.

HRMS-ESI (*m/z*): $[M + Na]^+$ calcd for $C_{26}H_{20}N_2O_4$ 447.1315; found, 447.1322.

7-Methoxy-3-(4-methoxyphenyl)-*N*-(quinolin-8-yl)benzofuran-2-carboxamide (**2p**)

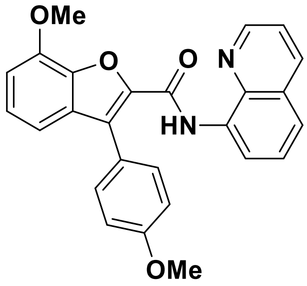

This compound was synthesized according to General procedure B using 10 mol% $Pd(OAc)_2$ for 24 h. Scale: 0.2 mmol. Isolated yield: 60% (51.0 mg). Purified by column chromatography (50-100% DCM in pentane).

^{1}H NMR (400 MHz, $CDCl_3$) δ: 11.05 (s, 1H), 8.91 (dd, *J* = 5.3, 3.7 Hz, 1H), 8.87 (dd, *J* = 4.2, 1.7 Hz, 1H), 8.18 (dd, *J* = 8.3, 1.7 Hz, 1H), 7.74 – 7.68 (m, 2H), 7.59 – 7.52 (m, 2H), 7.49 (dd, *J* = 8.2, 4.2 Hz, 1H), 7.30 – 7.23 (m, 2H + $CHCl_3$), 7.13 – 7.07 (m, 2H), 7.02 (dd, *J* = 6.1, 2.8 Hz, 1H), 4.19 (s, 3H), 3.92 (s, 3H).

^{13}C NMR (101 MHz, $CDCl_3$) δ: 159.9, 157.5, 148.5, 146.0, 143.6, 142.8, 138.9, 136.3, 134.5, 131.6, 131.1, 128.1, 127.5, 127.1, 124.5, 122.9, 122.0, 121.8, 117.1, 114.1 (two non-equivalent C's), 109.8, 56.7, 55.5.

HRMS-ESI (*m/z*): $[M + Na]^+$ calcd for $C_{26}H_{20}N_2O_4$ 447.1315; found, 447.1304.

5-Chloro-3-(4-methoxyphenyl)-*N*-(quinolin-8-yl)benzofuran-2-carboxamide (**2q**)

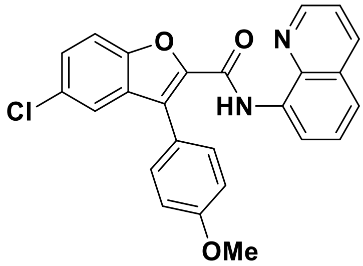

This compound was synthesized according to General procedure B using 10 mol% $Pd(OAc)_2$ for 24 h.. Scale: 0.2 mmol. Isolated yield: 83% (71.5 mg). Purified by column chromatography (0-30% EtOAc in pentane).

^{1}H NMR (400 MHz, $CDCl_3$) δ: 10.92 (s, 1H), 8.97 – 8.73 (m, 2H), 8.16 (d, *J* = 7.9 Hz, 1H), 7.71 – 7.40 (m, 8H), 7.09 (d, *J* = 8.2 Hz, 2H), 3.90 (s, 3H).

^{13}C NMR (101 MHz, $CDCl_3$) δ: 160.2, 157.1, 152.2, 148.4, 143.9, 138.8, 136.4, 134.3, 131.5, 130.7, 129.6, 128.1, 127.9, 127.5, 126.1, 122.2, 122.0, 121.8, 121.6, 117.2, 114.3, 113.4, 55.5.

HRMS-ESI (*m/z*): $[M + Na]^+$ calcd for $C_{25}H_{17}ClN_2O_3$ 451.0820; found, 451.0822.

tert-Butyl-(3-(4-methoxyphenyl)benzofuran-2-carbonyl)(quinolin-8-yl)carbamate

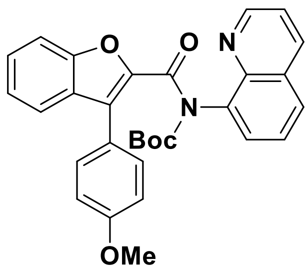

This compound was synthesized according to step 1 in General procedure C (2 h reaction time). Isolated by column chromatography (0-2% MeOH in DCM) Scale: 0.06 mmol. Isolated yield: 61% (25.5 mg) as a colourless solid.

^{1}H NMR (400 MHz, $CDCl_3$) δ: 8.88 (dd, *J* = 4.2, 1.7 Hz, 1H), 8.14 (dd, *J* = 8.3, 1.8 Hz, 1H), 7.81 (dd, *J* = 8.2, 1.4 Hz, 1H), 7.79 – 7.65 (m, 4H), 7.63 – 7.54 (m, 2H), 7.50 (ddd, *J* = 8.4, 7.2, 1.3 Hz, 1H), 7.40 – 7.33 (m, 2H), 7.04 – 6.97 (m, 2H), 3.83 (s, 3H), 1.27 (s, 9H).

^{13}C NMR (101 MHz, $CDCl_3$) δ: 163.5, 159.7, 153.8, 153.0, 150.4, 144.3, 144.0, 136.7, 136.1, 131.2, 129.2, 128.7, 128.3, 128.2, 127.4, 126.5, 126.2, 123.7, 122.9, 122.0, 121.7, 114.0, 112.2, 83.2, 55.4, 27.5.

HRMS-ESI (*m/z*): $[M + Na]^+$ calcd for $C_{30}H_{26}N_2O_5$ 517.1734; found, 517,1729.

N-benzyl-3-(4-methoxyphenyl)benzofuran-2-carboxamide (**3a**)

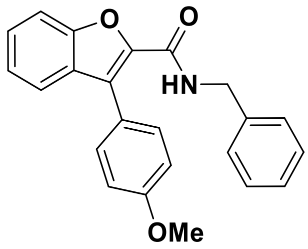

This compound was synthesized according to General procedure C (6 h reaction time). Scale: 0.1 mmol. Isolated yield: 92% (33 mg). Purified by column chromatography (0-40% EtOAc in pentane).

^{1}H NMR (400 MHz, $CDCl_3$) δ: 7.66 – 7.60 (m, 3H), 7.51 (dt, *J* = 8.4, 0.9 Hz, 1H), 7.44 (ddd, *J* = 8.4, 7.1, 1.3 Hz, 1H), 7.37 – 7.27 (m, 6H), 7.06 – 7.00 (m, 2H), 6.87 (t, *J* = 5.8 Hz, 1H), 4.62 (d, *J* = 5.8 Hz, 2H), 3.87 (s, 3H).

^{13}C NMR (101 MHz, $CDCl_3$) δ: 159.9, 159.3, 153.7, 142.1, 138.1, 131.5, 129.0, 128.9, 128.1, 127.7, 127.4, 125.9, 123.7, 122.6, 122.1, 114.0, 111.9, 55.5, 43.4.

HRMS-ESI (*m/z*): $[M + Na]^+$ calcd for $C_{23}H_{19}NO_3$ 380.1257; found, 380.1257.

3-(4-Methoxyphenyl)-*N*-(pyridin-2-ylmethyl)benzofuran-2-carboxamide (**3b**)

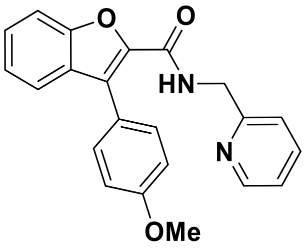

This compound was synthesized according to General procedure C (6 h reaction time). Scale: 0.1 mmol. Isolated yield: 90% (32 mg). Purified by column chromatography (0-50% EtOAc in pentane).

^{1}H NMR (400 MHz, $CDCl_3$) δ: 8.55 (ddd, *J* = 4.9, 1.8, 1.0 Hz, 1H), 7.75 (t, *J* = 5.2 Hz, 1H), 7.68 – 7.58 (m, 4H), 7.56 (dt, *J* = 8.3, 0.8 Hz, 1H), 7.44 (ddd, *J* = 8.4, 7.1, 1.3 Hz, 1H), 7.33 – 7.26 (m, 2H), 7.20 (ddd, *J* = 7.6, 4.9, 1.1 Hz, 1H), 7.05 – 7.00 (m, 2H), 4.74 (d, *J* = 5.2 Hz, 2H), 3.87 (s, 3H).

^{13}C NMR (101 MHz, $CDCl_3$) δ: 159.8, 159.4, 156.4, 153.7, 149.2, 142.3, 136.9, 131.5, 129.0, 127.3, 125.7, 123.6, 122.7, 122.5, 122.2, 122.0, 114.0, 112.0, 55.4, 44.4.

HRMS-ESI (*m/z*): $[M + Na]^+$ calcd for $C_{22}H_{18}N_2O_3$ 381.1210; found, 381.1210.

N-(4-Hydroxybenzyl)-3-(4-methoxyphenyl)benzofuran-2-carboxamide (**3c**)

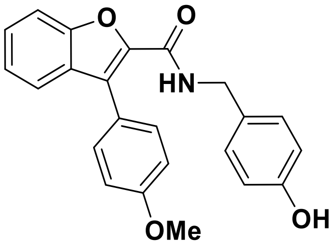

This compound was synthesized according to General procedure C (6 h aminolysis). Scale: 0.1 mmol. Isolated yield: 83% (31 mg). Purified by column chromatography (0-50% EtOAc in pentane).

^{1}H NMR (400 MHz, $CDCl_3$) δ: 7.66 – 7.55 (m, 3H), 7.50 (d, *J* = 8.3 Hz, 1H), 7.43 (ddd, *J* = 8.4, 7.0, 1.3 Hz, 1H), 7.29 (ddd, *J* = 8.0, 7.0, 1.1 Hz, 1H), 7.19 – 7.09 (m, 2H), 7.04 – 6.94 (m, 2H), 6.85 (t, *J* = 5.6 Hz, 1H), 6.80 – 6.69 (m, 2H), 6.13 (s, 1H), 4.51 (d, *J* = 5.7 Hz, 2H), 3.83 (s, 3H).

^{13}C NMR (101 MHz, $CDCl_3$) δ: 159.9, 159.5, 155.8, 153.7, 142.0, 131.5, 129.54, 129.46, 129.0, 127.5, 126.1, 123.8, 122.6, 122.1, 115.8, 114.1, 111.9, 55.4, 43.1.

HRMS-ESI (*m/z*): $[M + Na]^+$ calcd for $C_{23}H_{19}NO_4$ 396.1206; found, 396.1201.

N-(benzo[*d*][1,3]dioxol-5-ylmethyl)-3-(4-methoxyphenyl)benzofuran-2-carboxamide (**3d**)

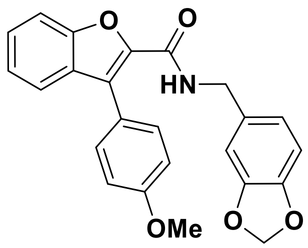

This compound was synthesized according to General procedure C (4 h reaction time). Scale: 0.1 mmol. Isolated yield: 72% (30 mg). Purified by column chromatography (10-50% EtOAc in pentane)

^{1}H NMR (400 MHz, $CDCl_3$) δ: 7.65 – 7.59 (m, 3H), 7.51 (dt, *J* = 8.4, 1.0 Hz, 1H), 7.44 (ddd, *J* = 8.3, 7.0, 1.3 Hz, 1H), 7.29 (ddd, *J* = 8.1, 7.1, 1.1 Hz, 1H), 7.06 – 7.00 (m, 2H), 6.86 – 6.76 (m, 4H), 5.95 (s, 2H), 4.51 (d, *J* = 5.8 Hz, 2H), 3.87 (s, 3H).

^{13}C NMR (101 MHz, $CDCl_3$) δ: 159.9, 159.2, 153.6, 148.1, 147.2, 142.1, 131.9, 131.5, 129.0, 127.4, 125.9, 123.7, 122.6, 122.1, 121.4, 114.0, 111.8, 108.7, 108.4, 101.2, 55.4, 43.2.

HRMS-ESI (*m/z*): $[M + Na]^+$ calcd for $C_{24}H_{19}NO_5$ 424.1155; found, 424.1155.

N-(2-(1*H*-Indol-3-yl)ethyl)-3-(4-methoxyphenyl)benzofuran-2-carboxamide (**3e**)

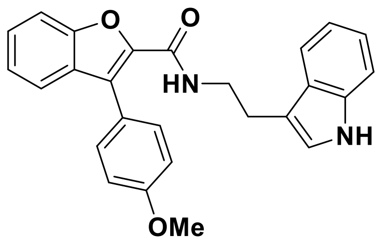

This compound was synthesized according to General procedure C (6 h reaction time). Scale: 0.1 mmol. Isolated yield: 56% (23 mg). Purified by column chromatography (10-40% EtOAc in pentane).

^{1}H NMR (400 MHz, $CDCl_3$) δ: 8.15 (s, 1H), 7.66 – 7.51 (m, 4H), 7.50 – 7.35 (m, 3H), 7.32 – 7.25 (m, 1H + $CHCl_3$), 7.27 – 7.17 (m, 1H), 7.18 – 7.09 (m, 1H), 7.04 – 6.93 (m, 3H), 6.69 (t, *J* = 5.9 Hz, 1H), 3.85 (s, 3H), 3.78 (q, *J* = 6.6 Hz, 2H), 3.06 (t, *J* = 6.7 Hz, 2H).

^{13}C NMR (101 MHz, $CDCl_3$) δ: 159.7, 159.4, 153.5, 142.2, 136.4, 131.3, 128.9, 127.3, 127.1, 125.2, 123.5, 122.6, 122.2, 122.1, 121.9, 119.5, 118.8, 113.9, 112.9, 111.8, 111.3, 55.4, 39.5, 25.4.

HRMS-ESI (*m/z*): $[M + Na]^+$ calcd for $C_{26}H_{22}N_2O_3$ 433.1523; found, 433.1524.

(3-(4-Methoxyphenyl)benzofuran-2-yl)(pyrrolidin-1-yl)methanone (**3f**)

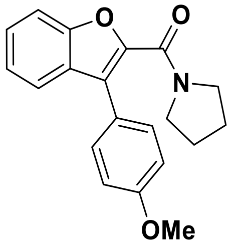

This compound was synthesized according to General procedure C (30 min reaction time). Scale: 0.15 mmol. Isolated yield: 86% (41 mg). Purified by column chromatography (20-60% EtOAc in pentane).

^{1}H NMR (400 MHz, $CDCl_3$) δ: 7.69 (dt, J = 7.8, 1.0 Hz, 1H), 7.60 – 7.51 (m, 3H), 7.41 (ddd, J = 8.4, 7.2, 1.3 Hz, 1H), 7.30 (ddd, J = 8.0, 7.2, 1.0 Hz, 1H), 7.04 – 6.98 (m, 2H), 3.87 (s, 3H), 3.62 (t, J = 6.9 Hz, 2H), 3.32 (t, J = 6.6 Hz, 2H), 1.90 – 1.73 (m, 4H).

^{13}C NMR (101 MHz, $CDCl_3$) δ: 160.6, 159.6, 154.2, 144.4, 130.5, 127.8, 126.3, 123.6, 123.5, 122.7, 121.3, 114.4, 112.0, 55.5, 47.9, 46.4, 26.2, 24.1.

HRMS-ESI (*m/z*): $[M + Na]^+$ calcd for $C_{20}H_{19}NO_3$ 344.1257; found, 344.1255.

(3-(4-Methoxyphenyl)benzofuran-2-yl)(piperidin-1-yl)methanone (**3g**)

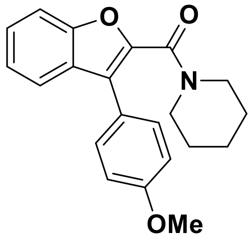

This compound was synthesized according to General procedure C (1 h reaction time). Scale: 0.15 mmol. Isolated yield: 94% (47 mg). Purified by column chromatography (20-60% EtOAc in pentane).

^{1}H NMR (400 MHz, $CDCl_3$) δ: 7.70 (ddd, J = 7.8, 1.3, 0.7 Hz, 1H), 7.59 – 7.48 (m, 3H), 7.39 (ddd, J = 8.4, 7.2, 1.3 Hz, 1H), 7.30 (ddd, J = 8.1, 7.2, 1.0 Hz, 1H), 7.06 – 6.97 (m, 2H), 3.87 (s, 3H), 3.66 (s, 2H), 3.17 (s, 2H), 1.64 – 1.46 (m, 4H), 1.15 (s, 2H).

^{13}C NMR (101 MHz, DMSO-*d6*) δ: 159.8, 159.2, 153.5, 143.6, 129.7, 126.4, 126.0, 123.8, 122.3, 120.7, 119.4, 114.6, 111.8, 55.2, 47.2, 42.3, 25.6, 25.0, 23.8.

HRMS-ESI (*m/z*): $[M + Na]^+$ calcd for $C_{21}H_{21}NO_3$ 358.1414; found, 358.1412.

(3-(4-Methoxyphenyl)benzofuran-2-yl)(morpholino)methanone (**3h**)

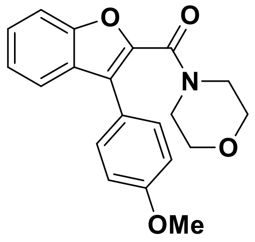

This compound was synthesized according to General procedure C (6 h reaction time). Scale: 0.15 mmol. Isolated yield: 97% (49 mg). Purified by column chromatography (20-60% EtOAc in pentane).

^{1}H NMR (400 MHz, $CDCl_3$) δ: 7.69 (ddd, *J* = 7.8, 1.2, 0.7 Hz, 1H), 7.56 (dt, *J* = 8.3, 0.8 Hz, 1H), 7.54 – 7.46 (m, 2H), 7.42 (ddd, *J* = 8.4, 7.2, 1.3 Hz, 1H), 7.31 (ddd, *J* = 8.1, 7.2, 1.0 Hz, 1H), 7.08 – 6.98 (m, 2H), 3.87 (s, 3H), 3.69 (d, *J* = 25.3 Hz, 4H), 3.23 (s, 4H).

^{13}C NMR (101 MHz, $CDCl_3$) δ: 161.3, 159.9, 154.6, 142.6, 130.3, 127.2, 126.3, 123.7, 123.0, 122.3, 121.1, 114.7, 112.2, 66.5, 55.5, 47.4, 42.7. (Hindered rotation causes equivalent carbons to be non-equivalent)

HRMS-ESI (*m/z*): $[M + Na]^+$ calcd for $C_{20}H_{19}NO_4$ 360.1206; found, 360.1221.

Methyl 4-(2-(benzylcarbamoyl)benzofuran-3-yl)benzoate (**3i**)

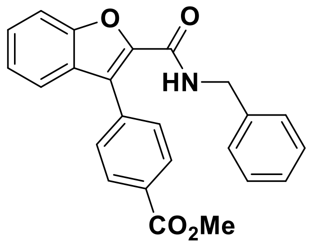

This compound was synthesized according to General procedure C (6 h reaction time). Scale: 0.1 mmol. Isolated yield: 88% (33 mg). Purified by column chromatography (0-40% EtOAc in pentane).

^{1}H NMR (400 MHz, $CDCl_3$) δ: δ 8.20 – 8.13 (m, 2H), 7.80 – 7.73 (m, 2H), 7.59 (dt, *J* = 7.9, 1.1 Hz, 1H), 7.52 (dt, *J* = 8.3, 0.9 Hz, 1H), 7.47 (ddd, *J* = 8.4, 6.9, 1.3 Hz, 1H), 7.39 – 7.28 (m, 6H), 7.00 (t, *J* = 5.8 Hz, 1H), 4.63 (d, *J* = 5.9 Hz, 2H), 3.96 (s, 3H).

^{13}C NMR (101 MHz, $CDCl_3$) δ: 167.0, 158.9, 153.6, 142.6, 137.9, 135.5, 130.3, 130.0, 129.6, 128.9, 128.4, 128.1, 127.8, 127.7, 125.3, 124.1, 121.8, 111.9, 52.3, 43.4.

HRMS-ESI (*m/z*): $[M + Na]^+$ calcd for $C_{24}H_{19}NO_4$ 4083.1206; found, 408.1207.

N-Benzyl-3-(thiophen-2-yl)benzofuran-2-carboxamide (**3j**)

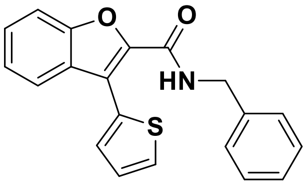

This compound was synthesized according to General procedure C (6 h reaction time). Scale: 0.66 mmol. Isolated yield: 60% (127 mg). Purified by column chromatography (0-30% EtOAc in pentane).

^{1}H NMR (400 MHz, $CDCl_3$) δ: 7.91 (dt, *J* = 7.9, 1.0 Hz, 1H), 7.75 (dd, *J* = 3.6, 1.2 Hz, 1H), 7.56 – 7.42 (m, 3H), 7.42 – 7.28 (m, 6H), 7.20 (dd, *J* = 5.2, 3.6 Hz, 1H), 7.02 (t, *J* = 5.5 Hz, 1H), 4.67 (d, *J* = 5.9 Hz, 2H).

^{13}C NMR (101 MHz, $CDCl_3$) δ: 159.0, 153.4, 142.3, 137.9, 130.9, 129.9, 128.8, 128.3, 128.1, 127.7, 127.6, 127.3, 124.0, 122.5, 119.4, 111.8, 43.4.

HRMS-ESI (*m/z*): $[M + Na]^+$ calcd for $C_{20}H_{15}NO_2S$ 356.0716; found, 356.0716.

*Basic hydrolysis of amide (**2a**) to carboxylic acid (**4**)*
3-(4-Methoxyphenyl)benzofuran-2-carboxylic acid (**4**)

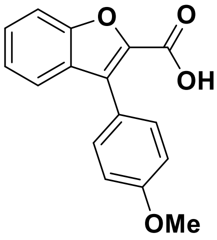

Compound **2a** (70.0 mg, 0.18 mmol) and NaOH (109 mg, 15 eq)were dissolved in dry ethanol (0.9 mL) and stirred for 24 h at 80 °C. The product was isolated by extraction. Isolated yield: 77% (36.7 mg, 0.14 mmol).

^{1}H NMR (400 MHz, DMSO-d_6) δ: 7.74 – 7.65 (m, 1H), 7.59 – 7.53 (m, 3H), 7.50 (ddd, *J* = 8.4, 7.2, 1.3 Hz, 1H), 7.33 (ddd, *J* = 8.0, 7.2, 1.0 Hz, 1H), 7.07 – 7.01 (m, 2H), 3.83 (s, 3H), 3.39 (s, 1H).

^{13}C NMR (101 MHz, DMSO-d_6) δ: 161.1, 159.0, 153.4, 142.4, 131.3, 128.1, 127.2, 125.4, 123.6, 122.9, 121.5, 113.5, 112.0, 55.2.

HRMS-ESI (*m/z*): $[M + Na]^+$ calcd for $C_{16}H_{12}O_4$ 291.0628; found, 291.0635.

NMR Spectra

N-(Quinolin-8-yl)benzofuran-2-carboxamide (**1a**)

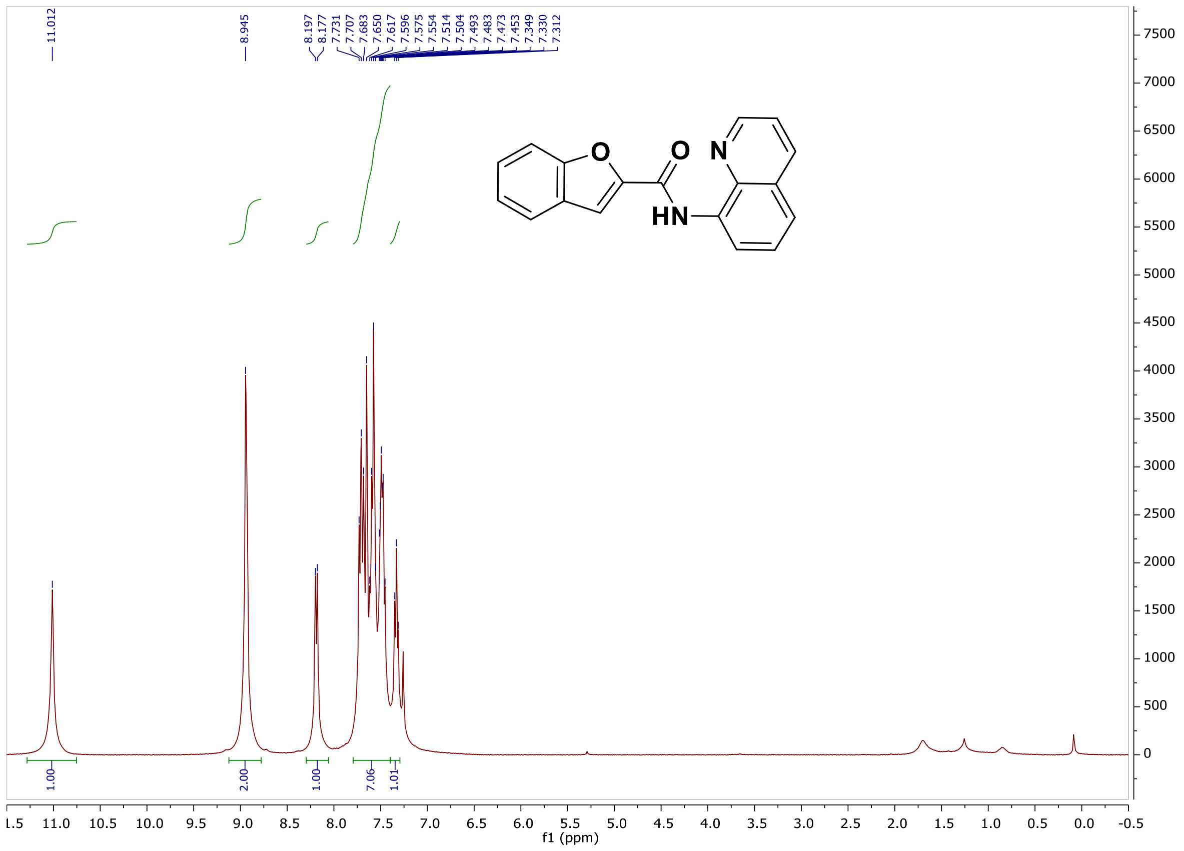

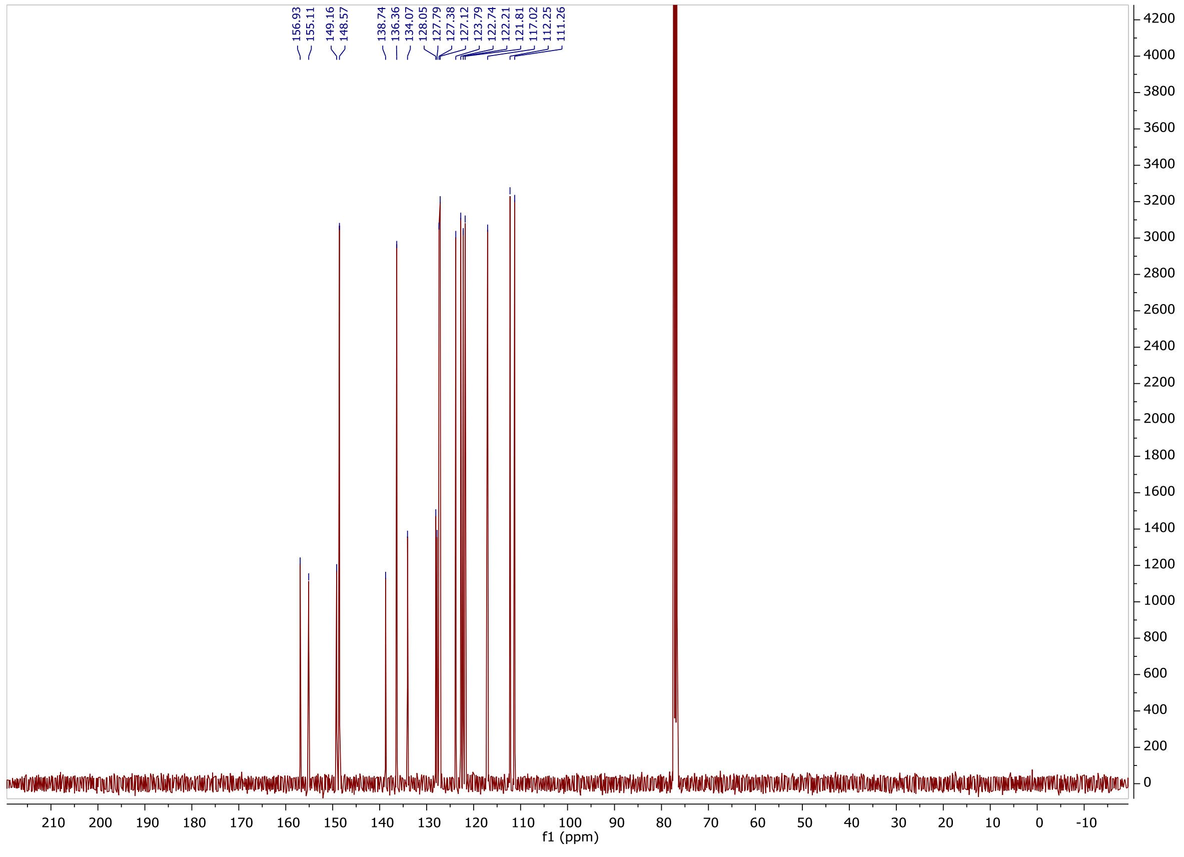

7-Methoxy-*N*-(quinolin-8-yl)benzofuran-2-carboxamide (**1b**)

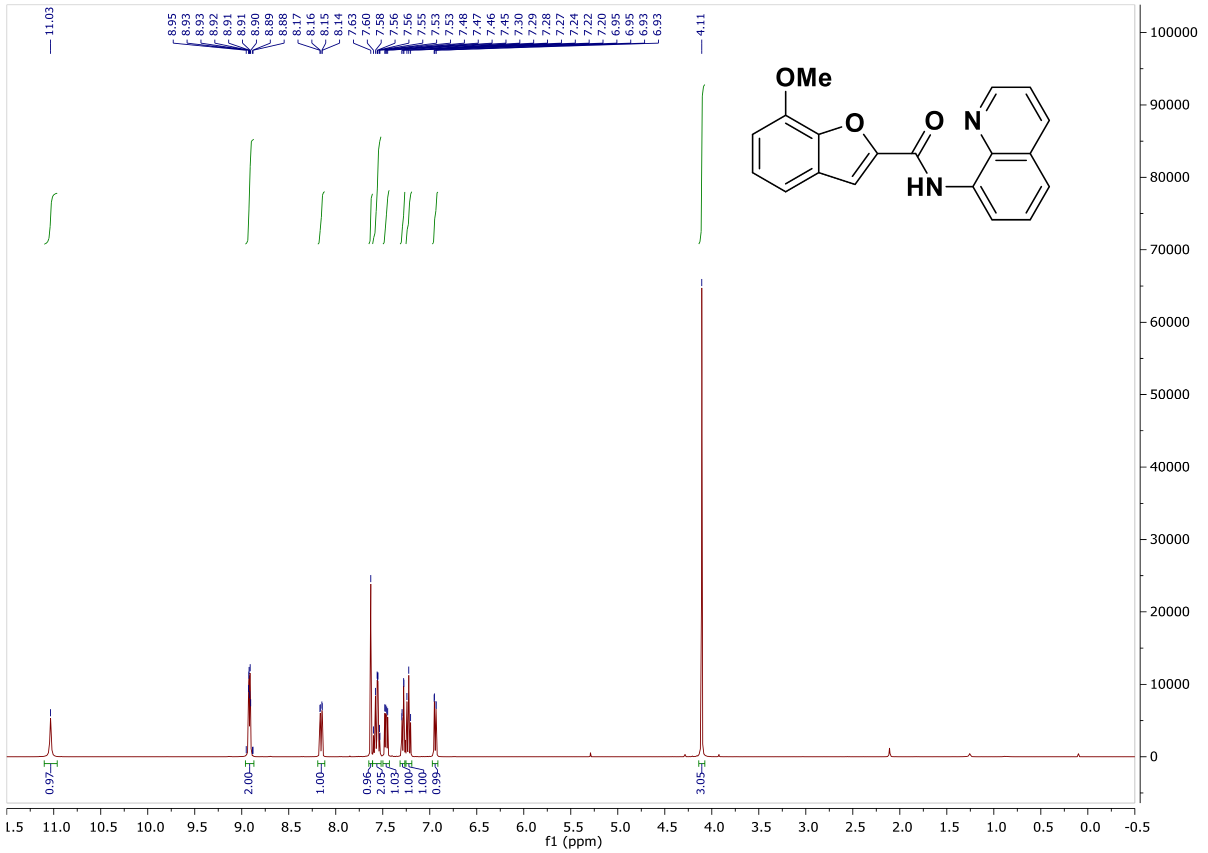

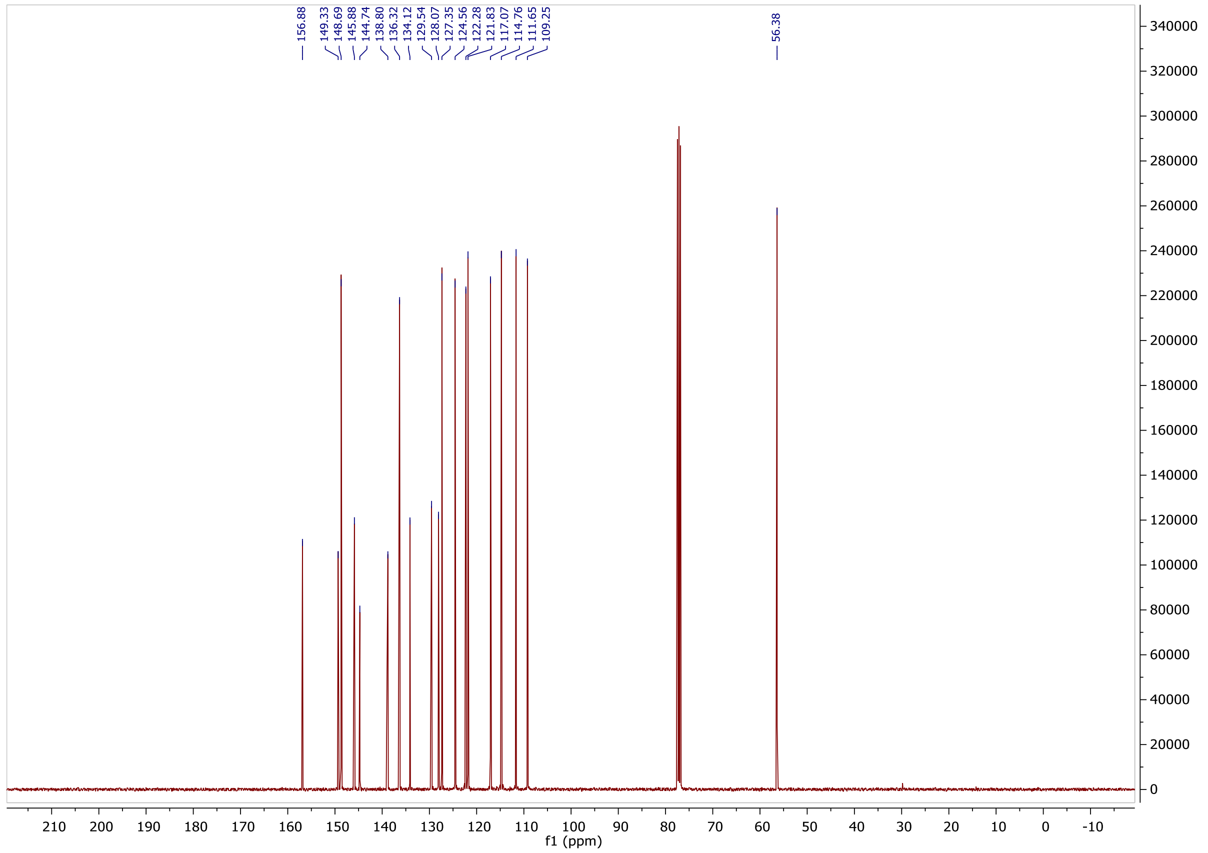

5-Methoxy-*N*-(quinolin-8-yl)benzofuran-2-carboxamide (**1c**)

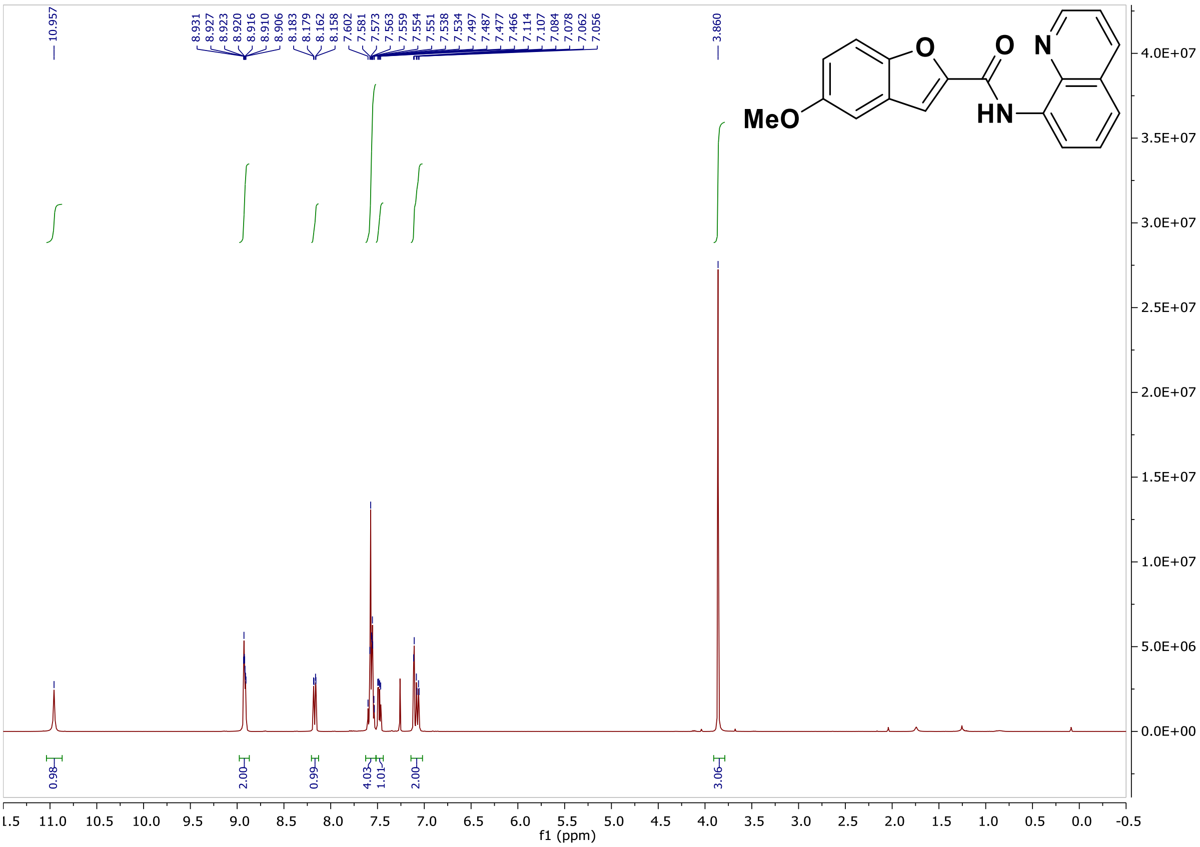

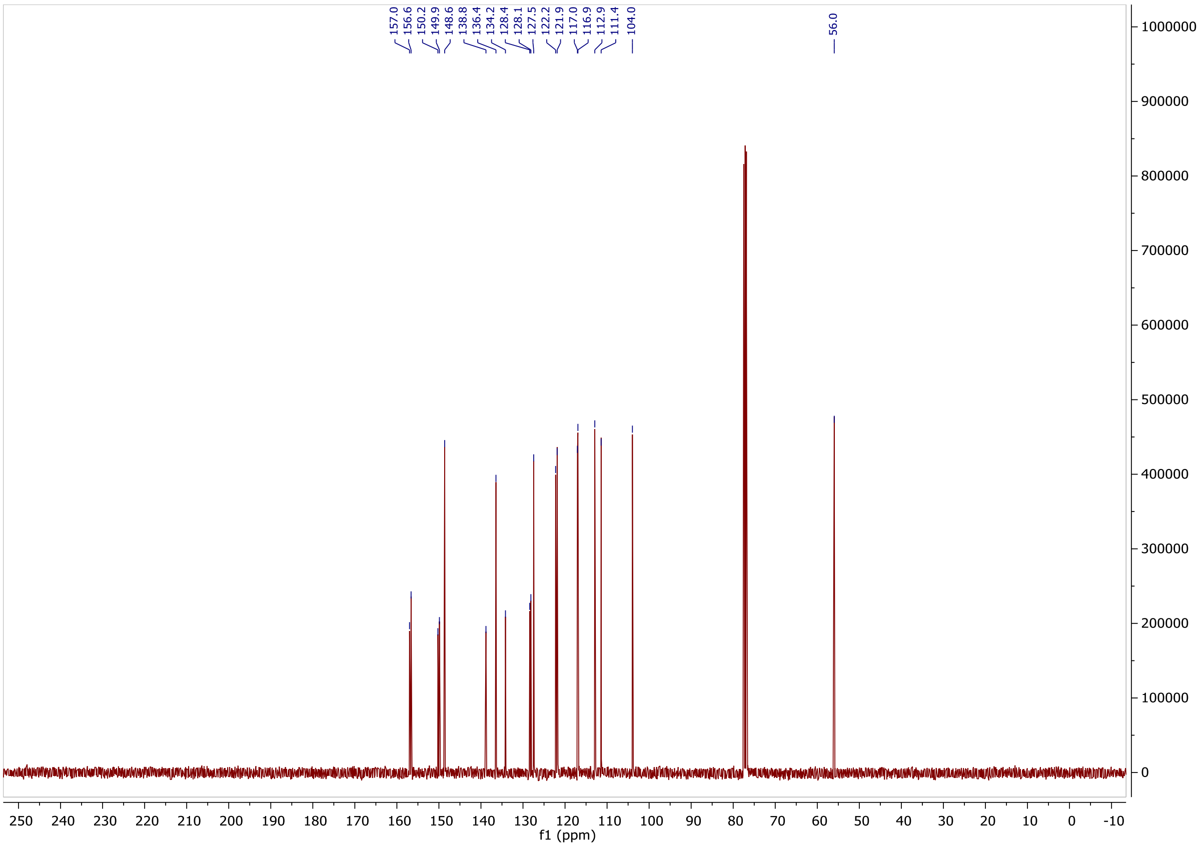

5-Chloro-*N*-(quinolin-8-yl)benzofuran-2-carboxamide (**1d**)

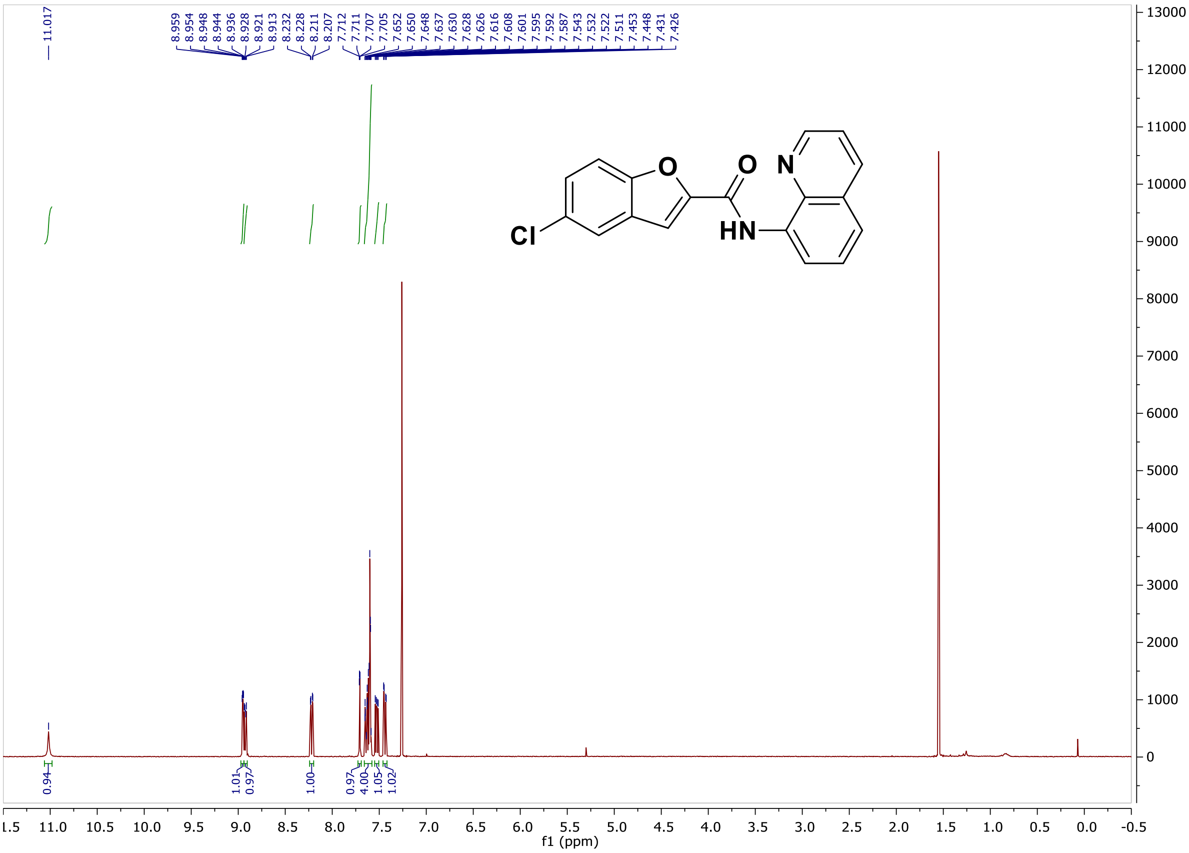

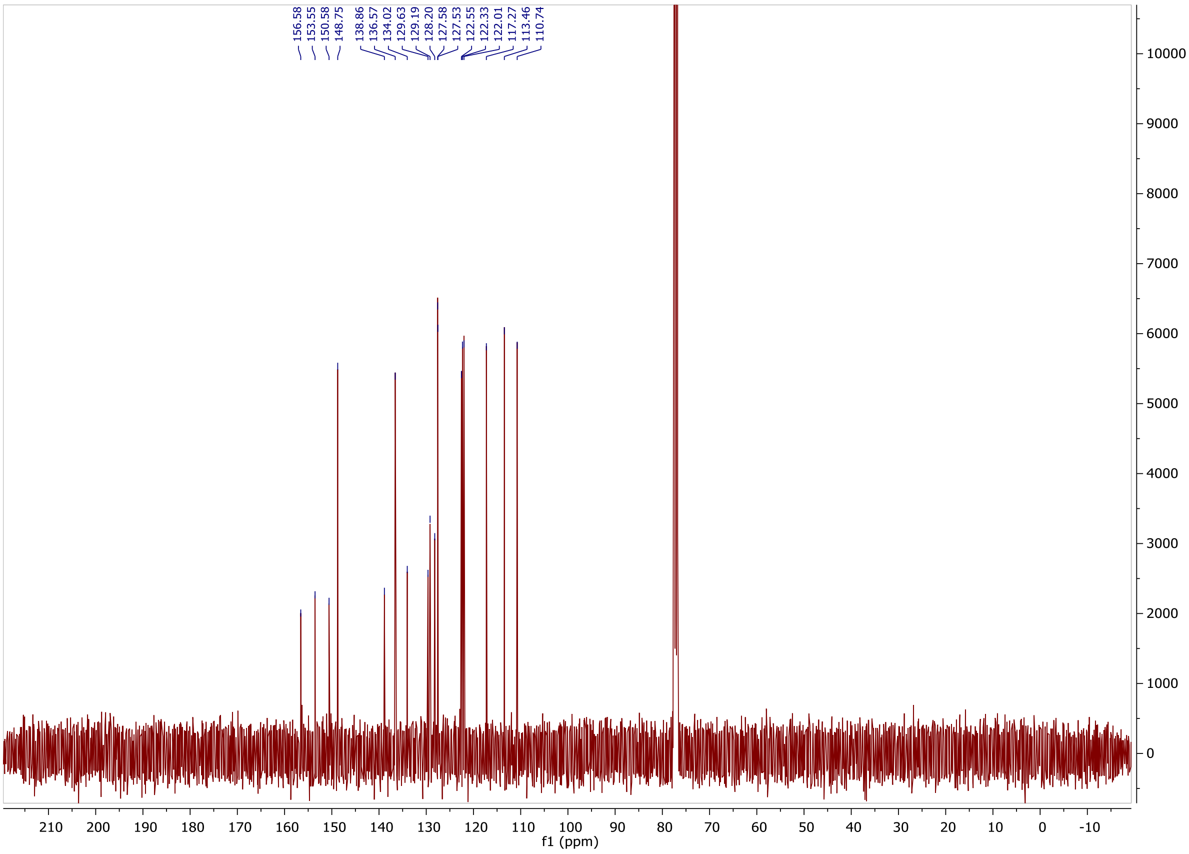

N-Phenylbenzofuran-2-carboxamide (**1e**)

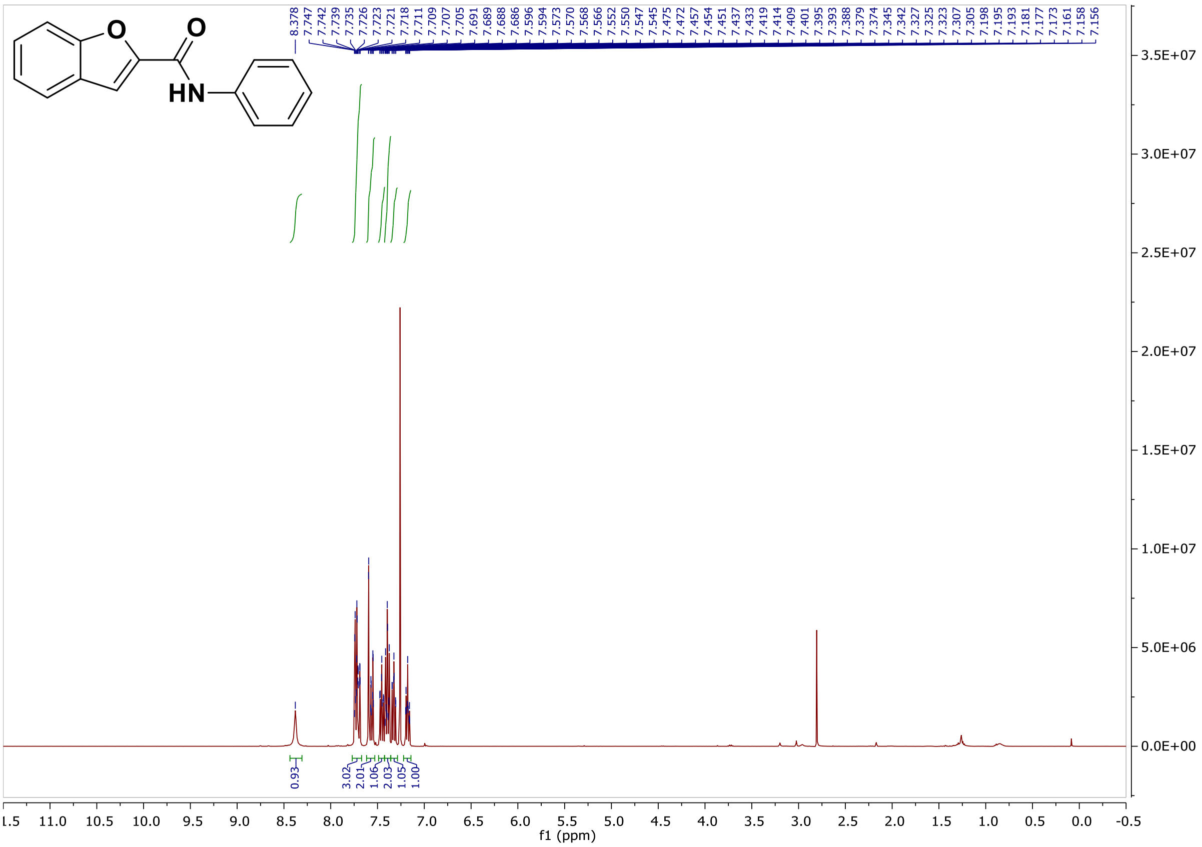

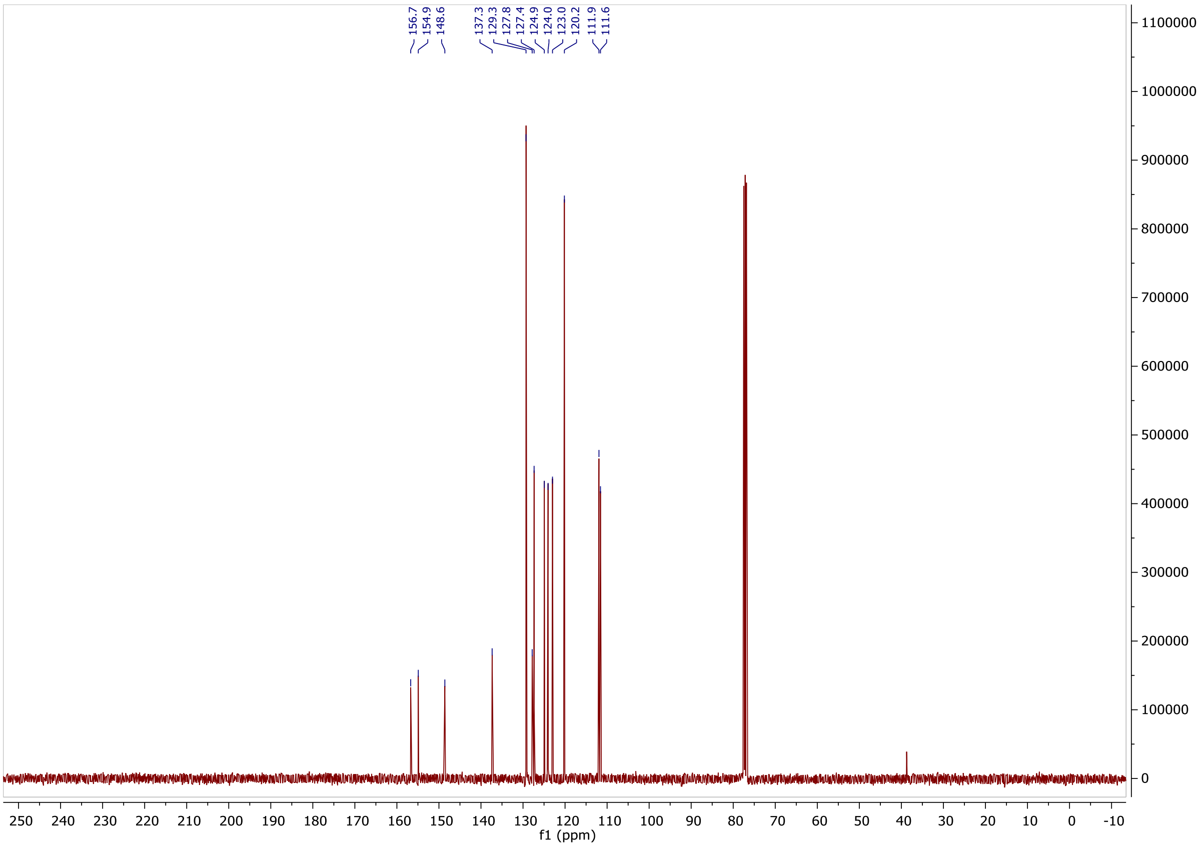

3-(4-Methoxyphenyl)-*N*-(quinolin-8-yl)benzofuran-2-carboxamide (**2a**)

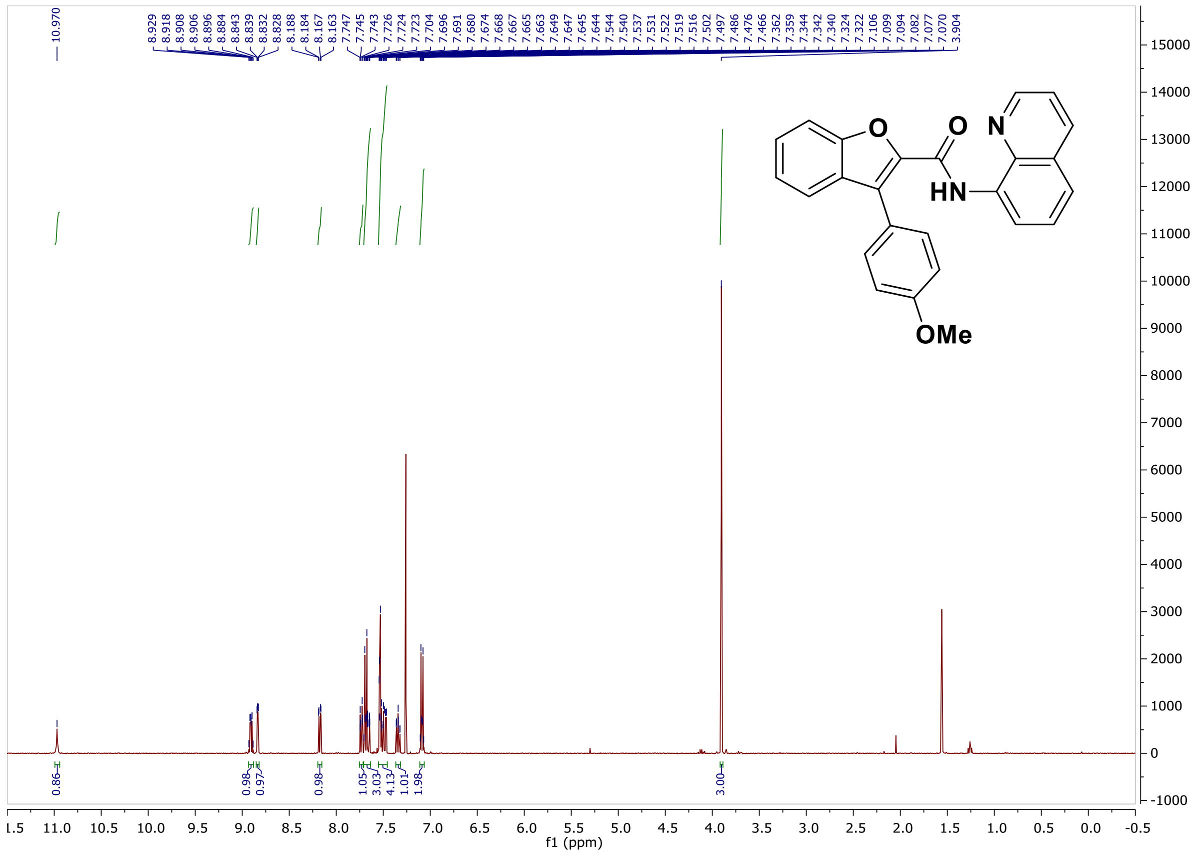

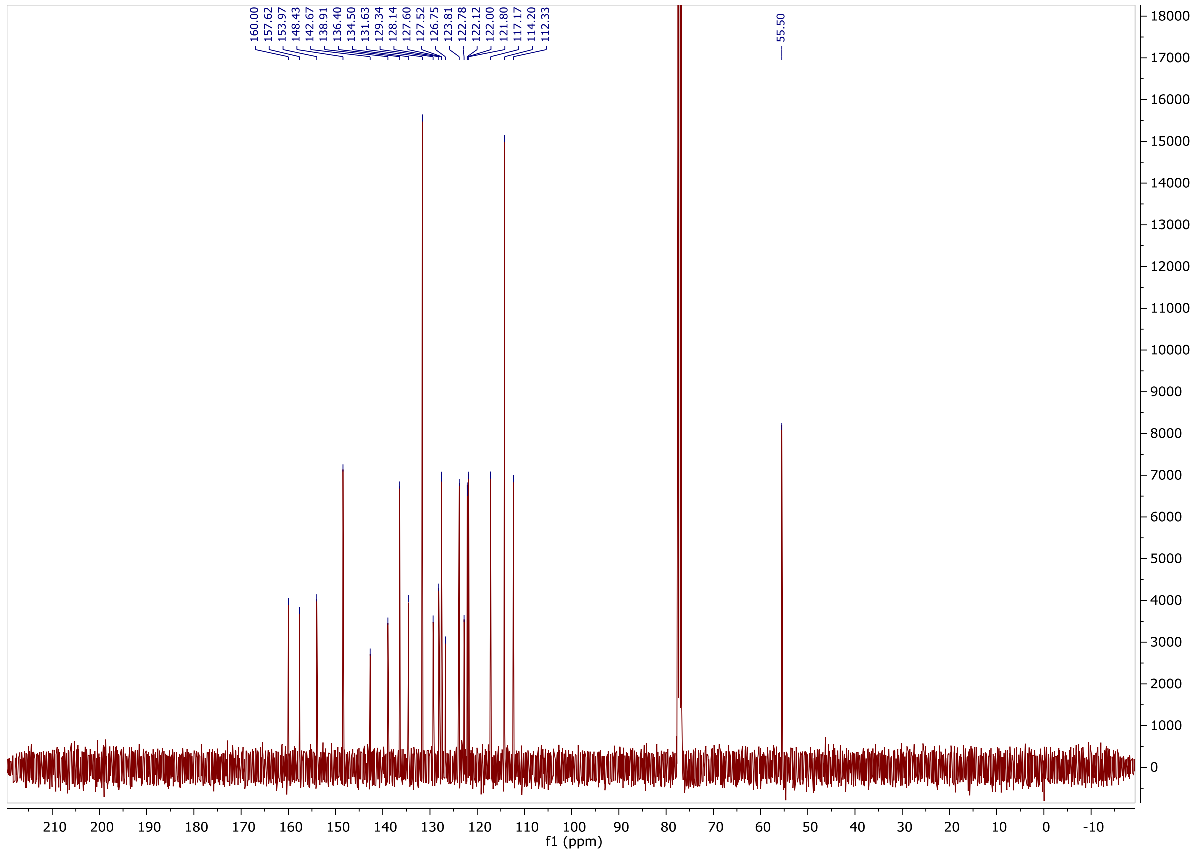

3-(3,5-Dimethylphenyl)-*N*-(quinolin-8-yl)benzofuran-2-carboxamide (**2b**)

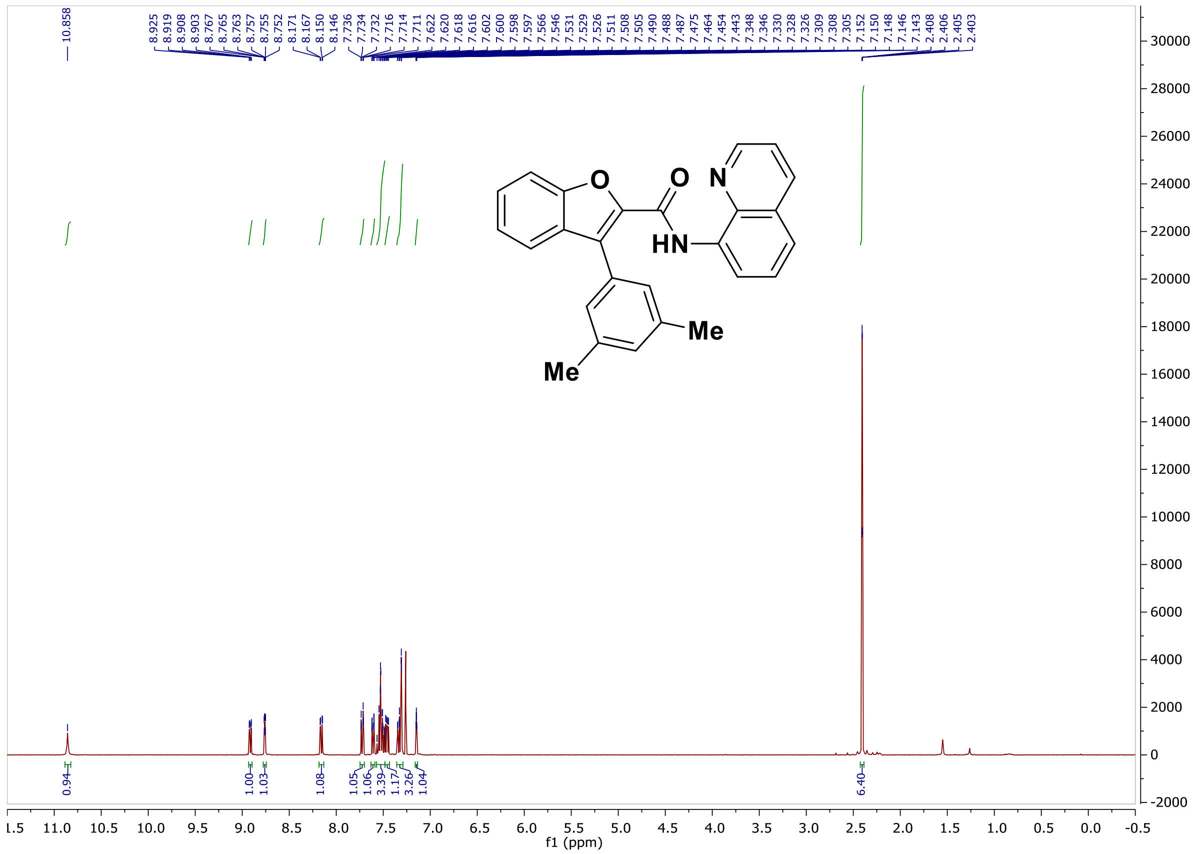

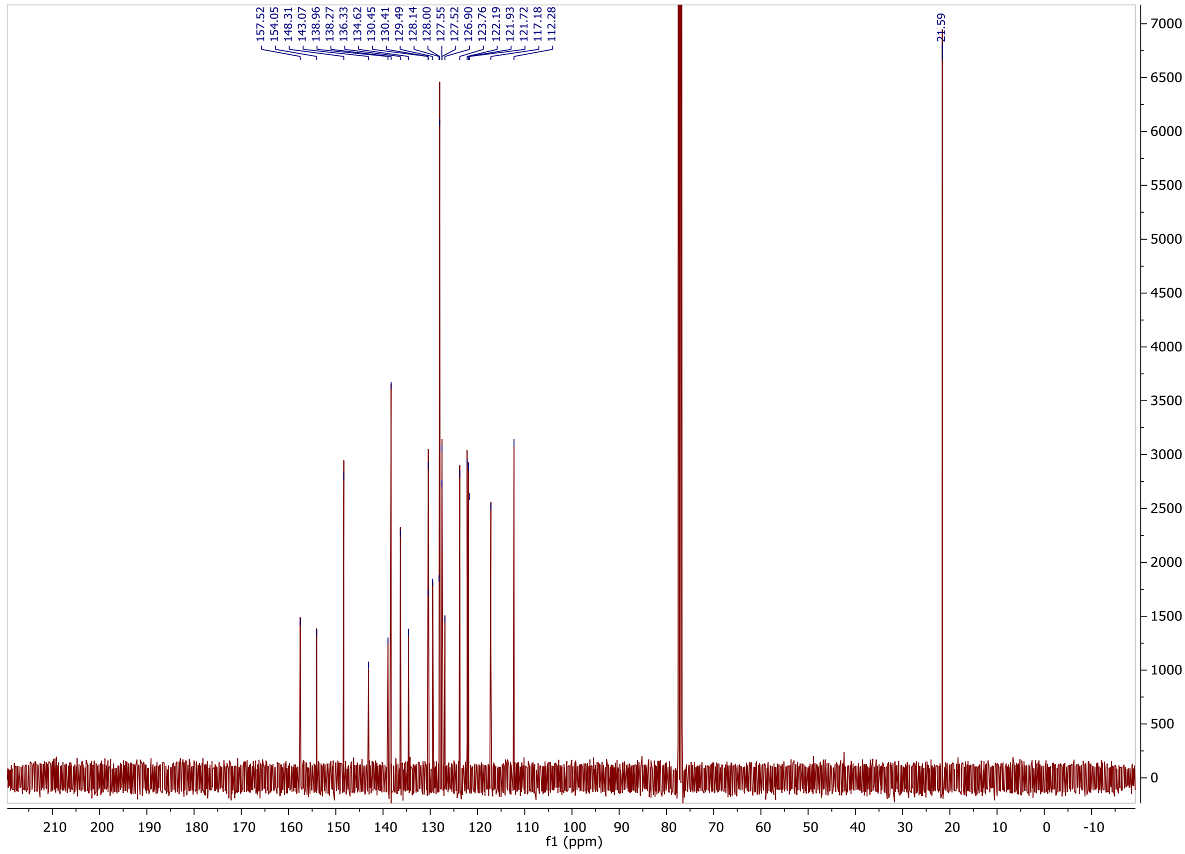

N-(Quinolin-8-yl)-3-(p-tolyl)benzofuran-2-carboxamide (**2c**)

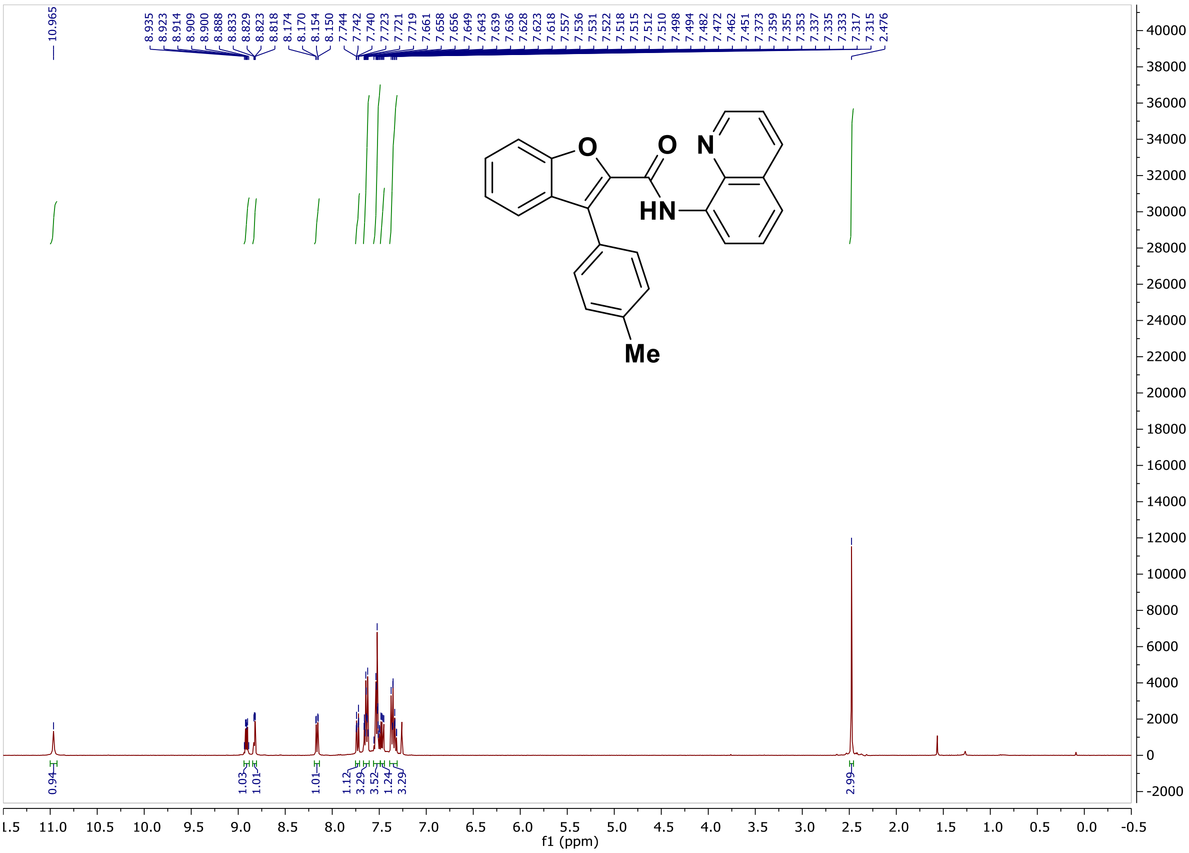

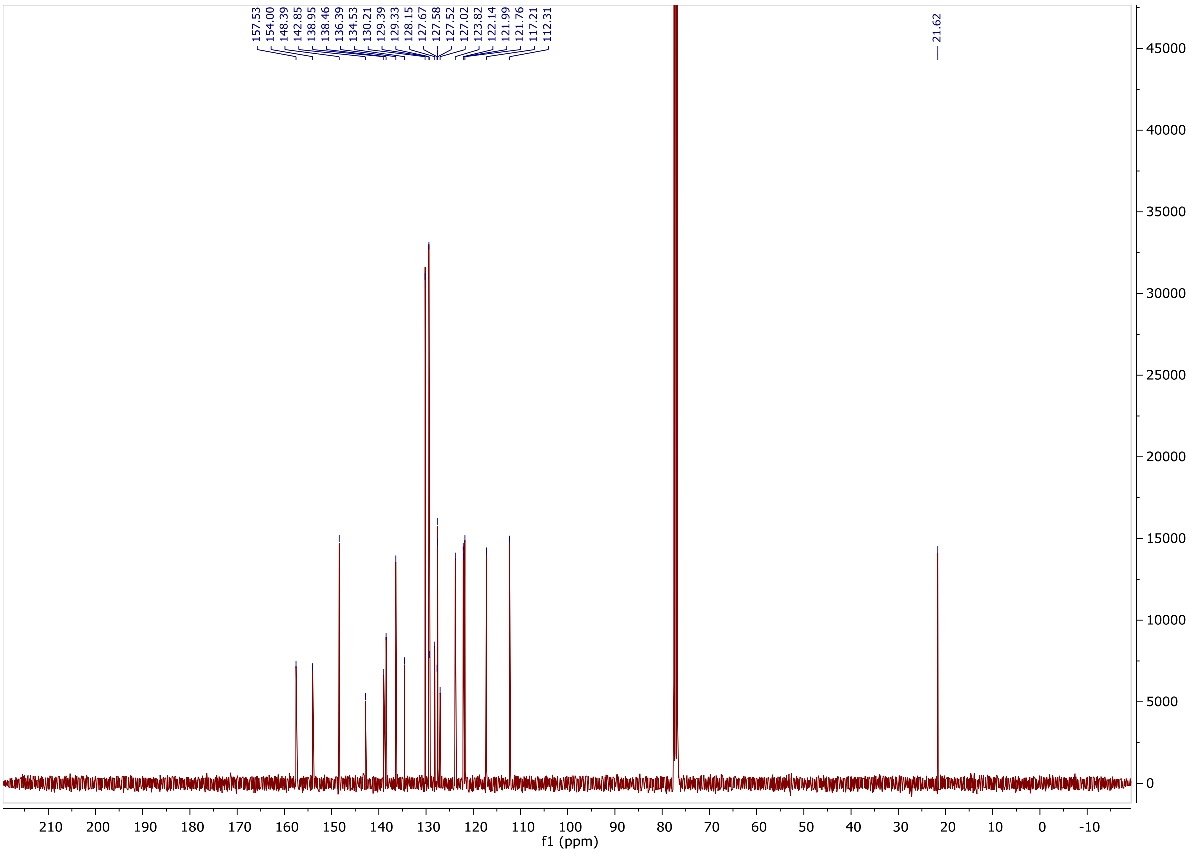

3-Phenyl-*N*-(quinolin-8-yl)benzofuran-2-carboxamide (**2d**)

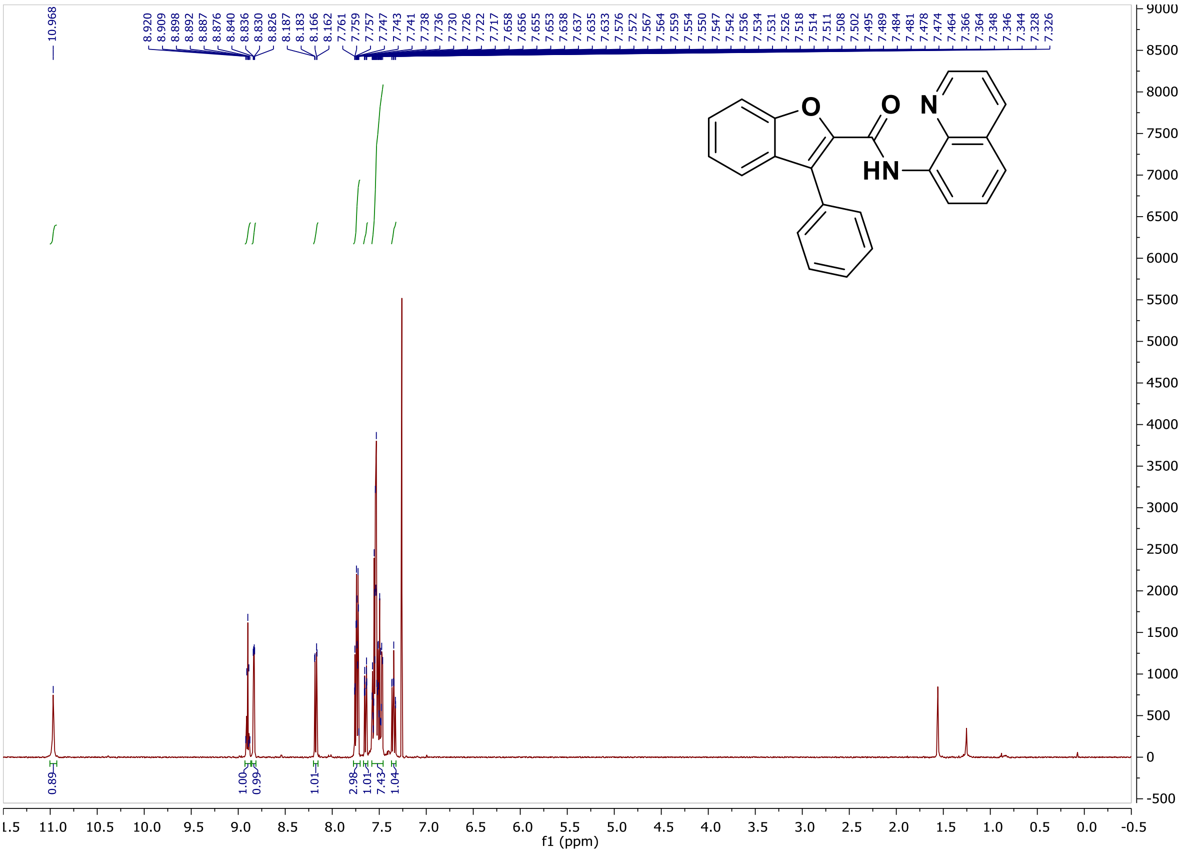

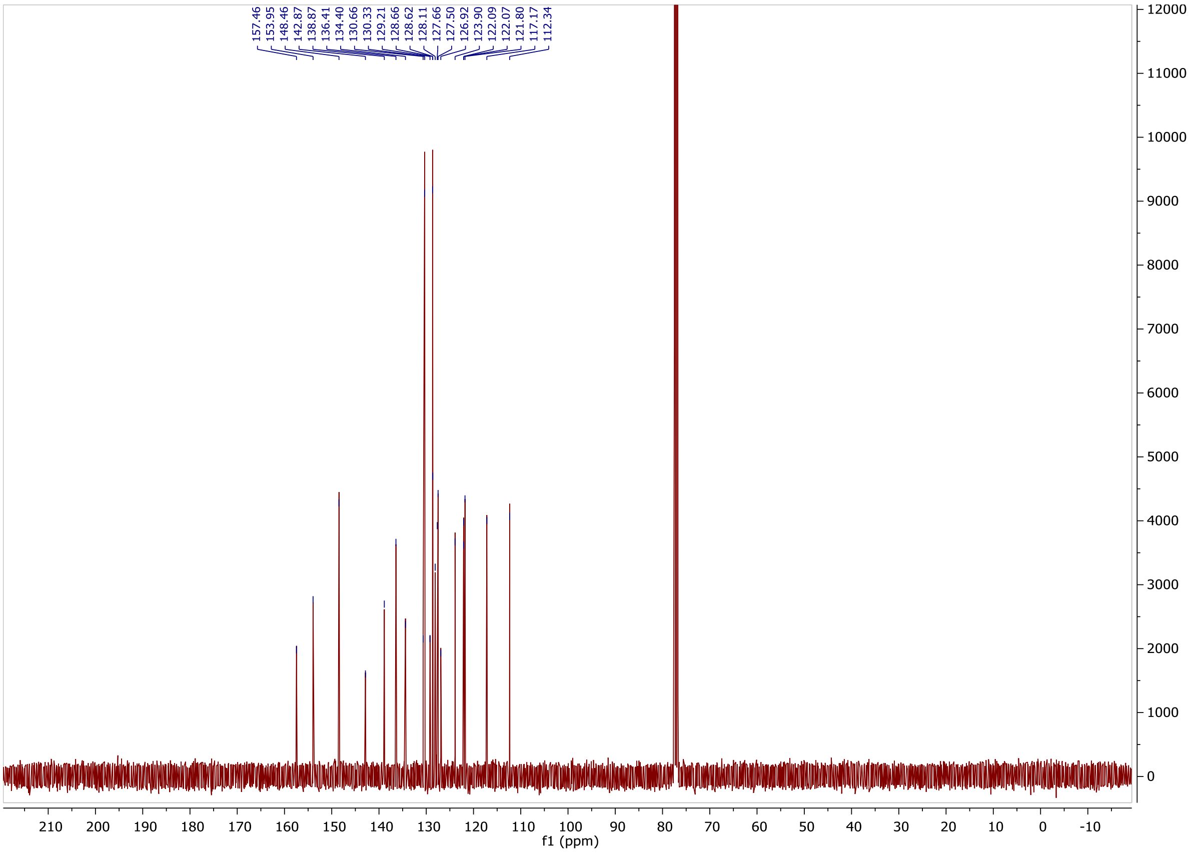

3-(Naphthalen-2-yl)-*N*-(quinolin-8-yl)benzofuran-2-carboxamide (**2e**)

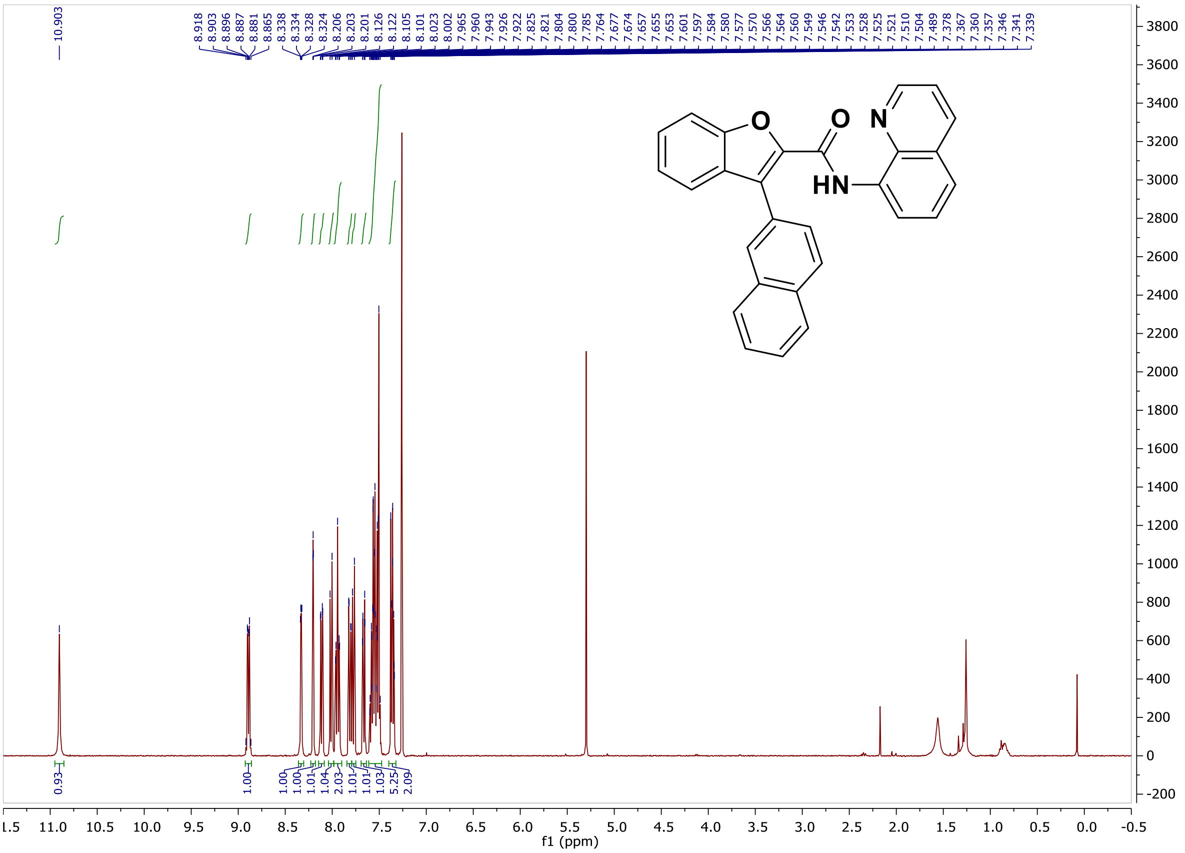

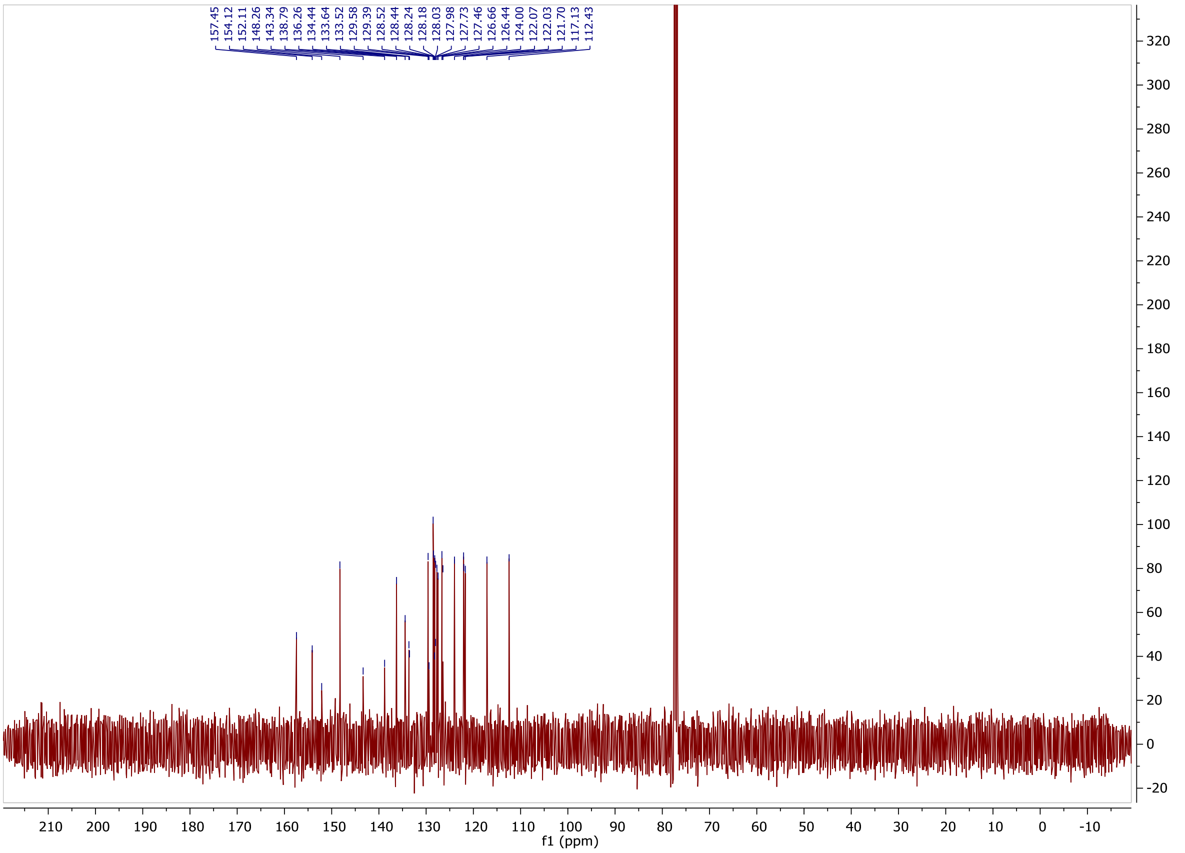

3-(3-Fluorophenyl)-*N*-(quinolin-8-yl)benzofuran-2-carboxamide (**2f**)

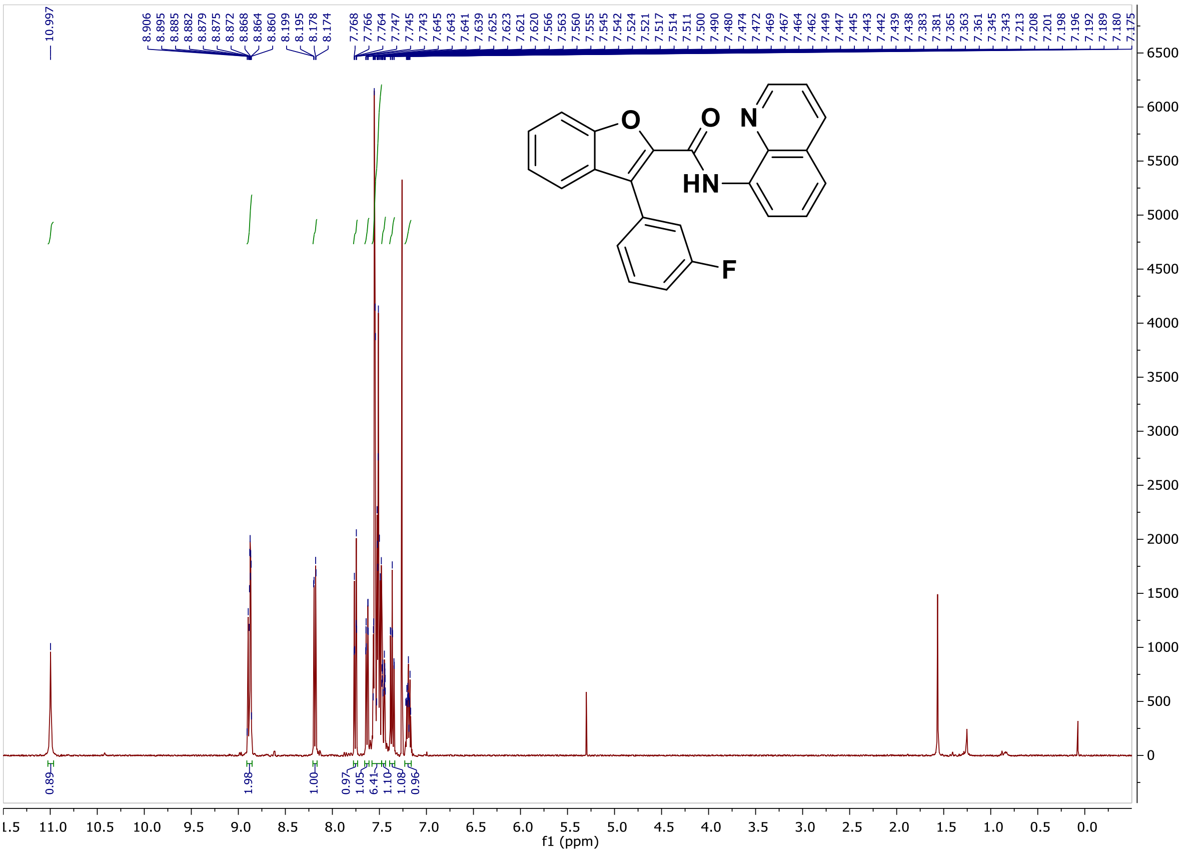

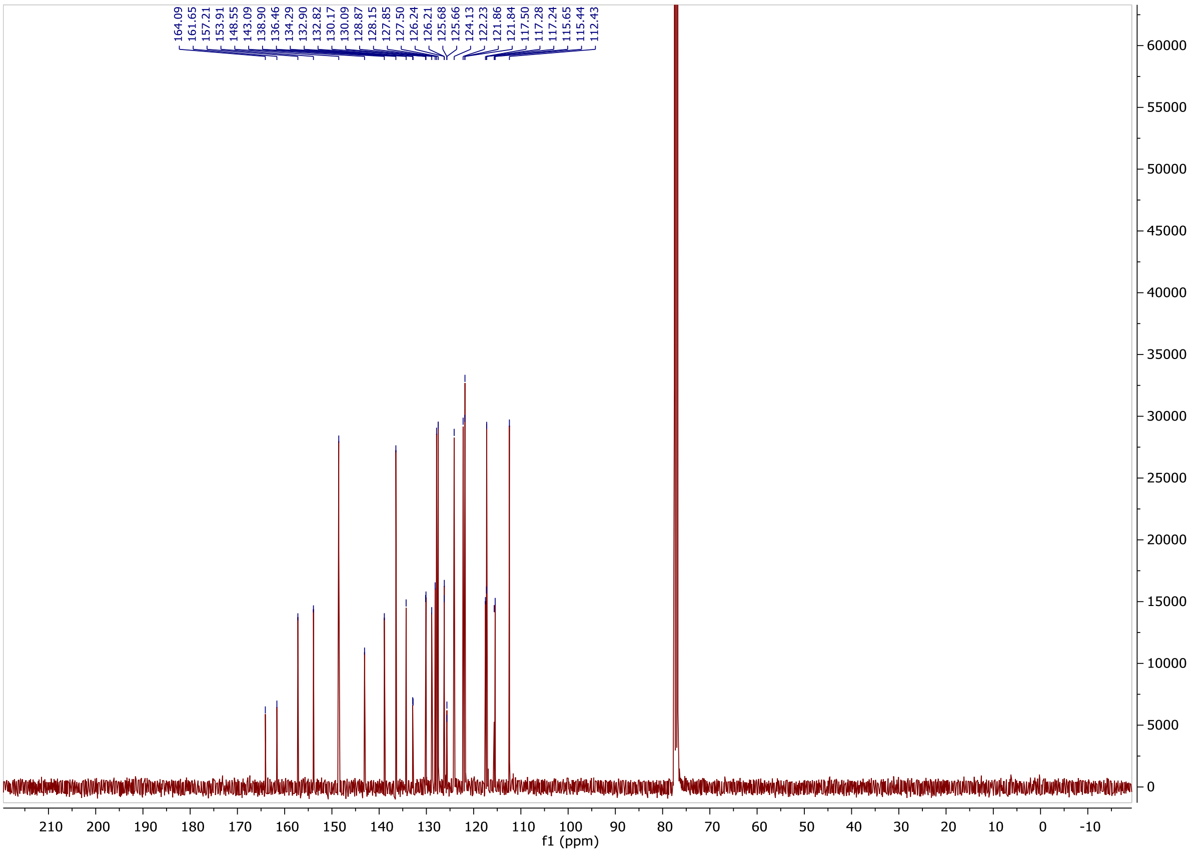

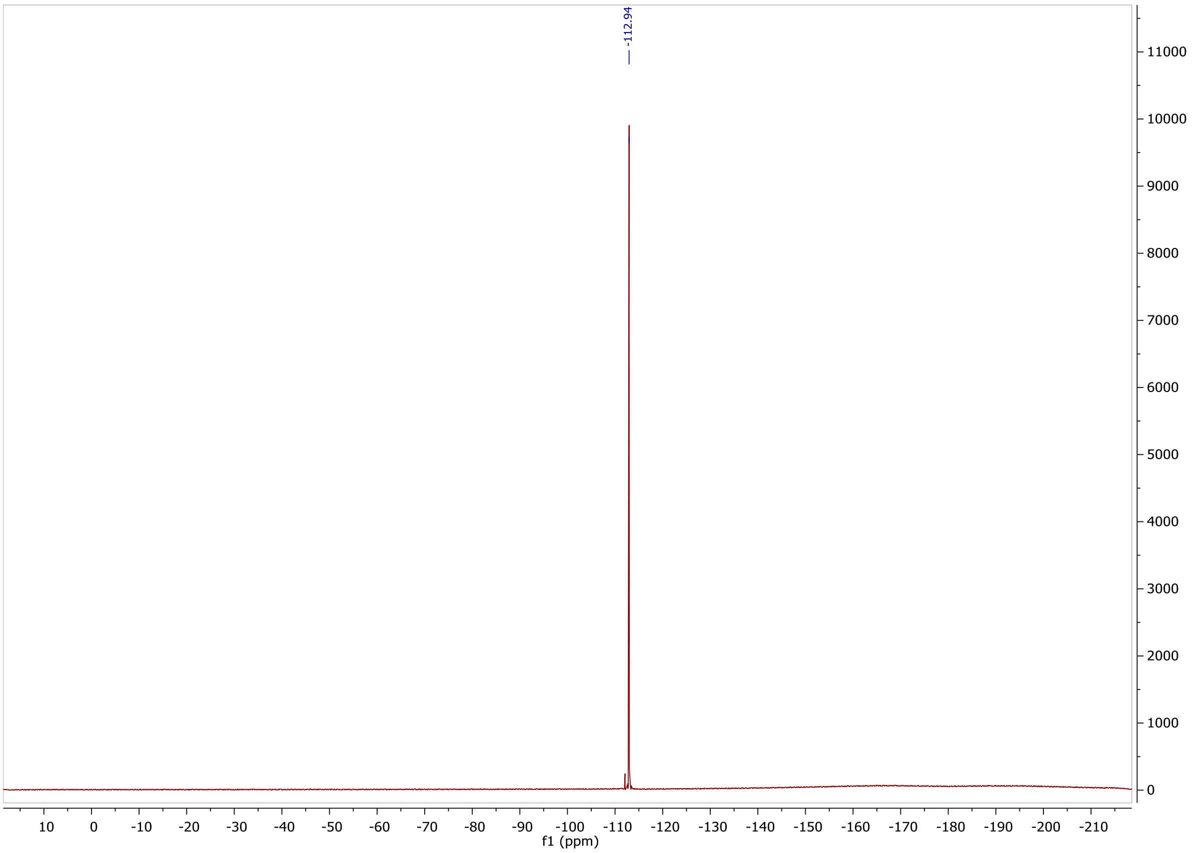

-112.94
11000
10000
9000
8000
7000
6000
5000
4000
3000
2000
1000
0
10 0 -10 -20 -30 -40 -50 -60 -70 -80 -90 -100 -110 -120 -130 -140 -150 -160 -170 -180 -190 -200 -210
f1 (ppm)

3-(4-Chlorophenyl)-*N*-(quinolin-8-yl)benzofuran-2-carboxamide (**2g**)

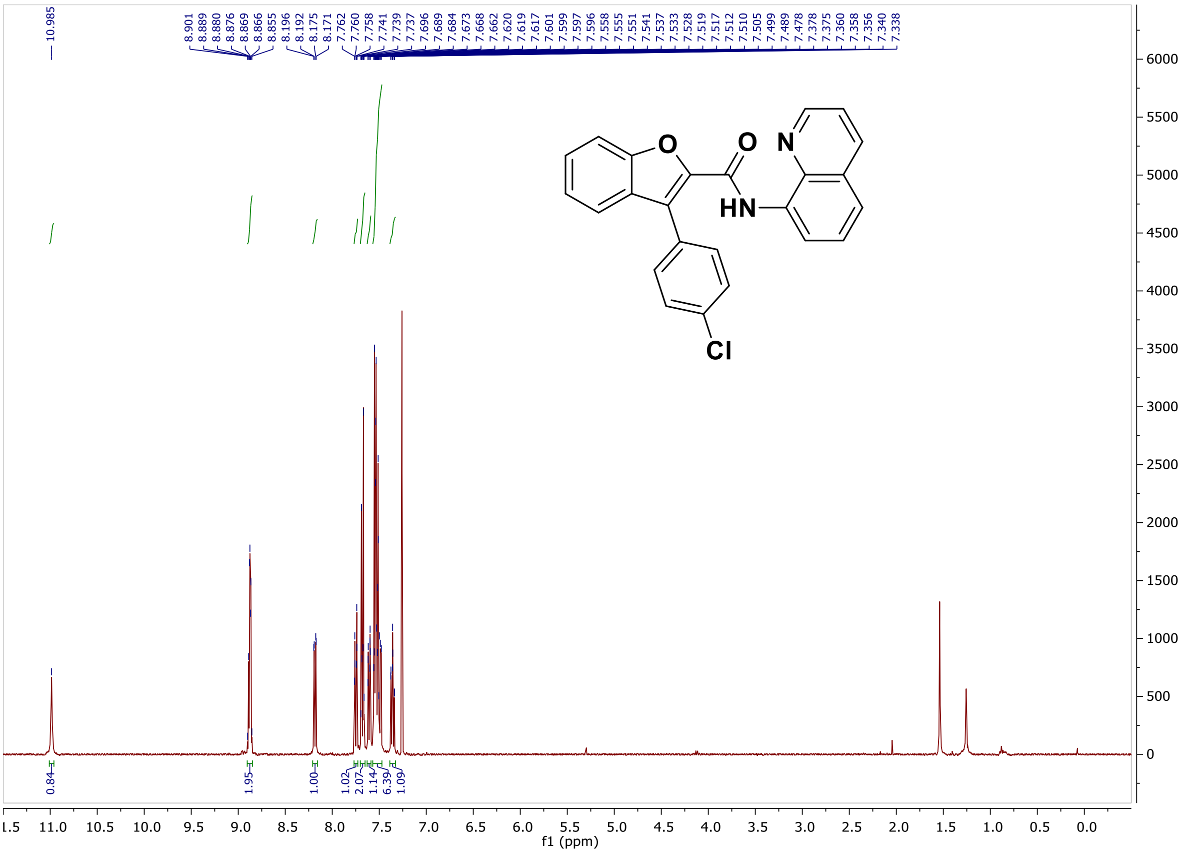

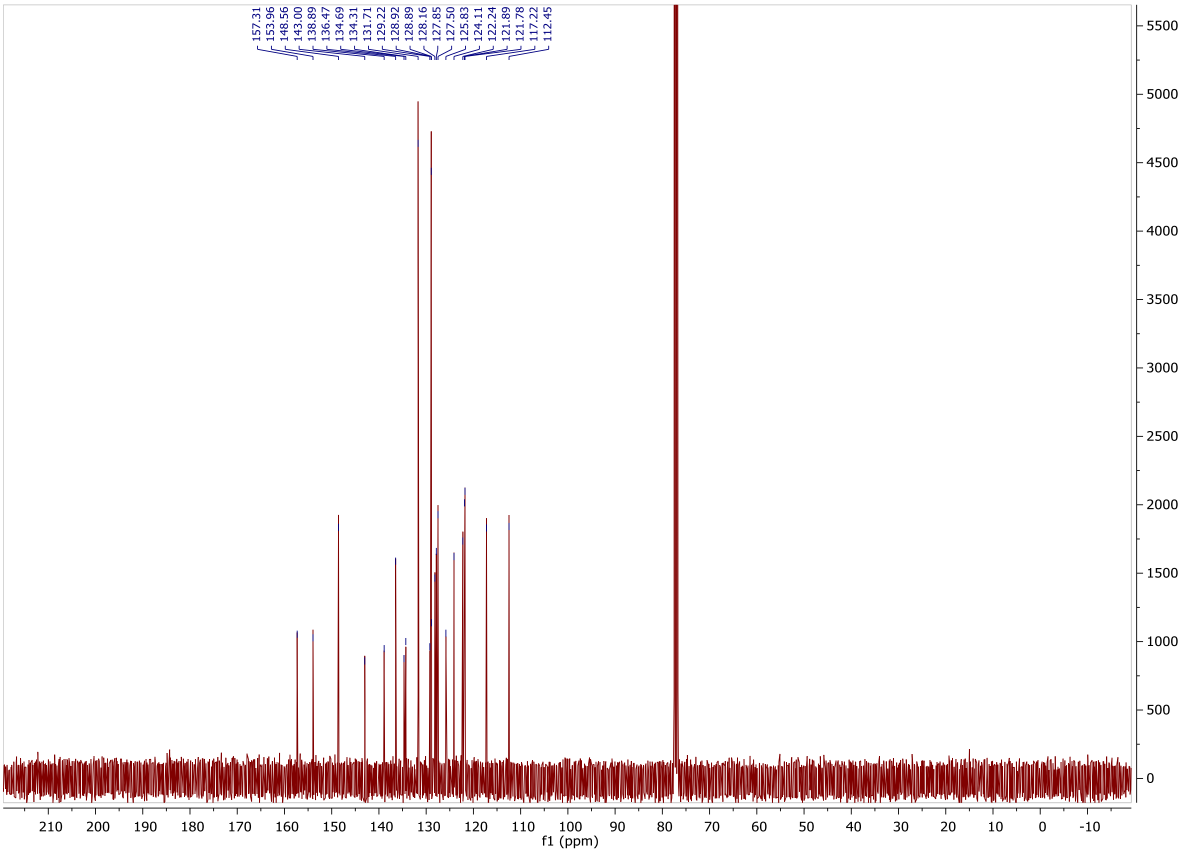

3-(4-Bromophenyl)-*N*-(quinolin-8-yl)benzofuran-2-carboxamide (**2h**)

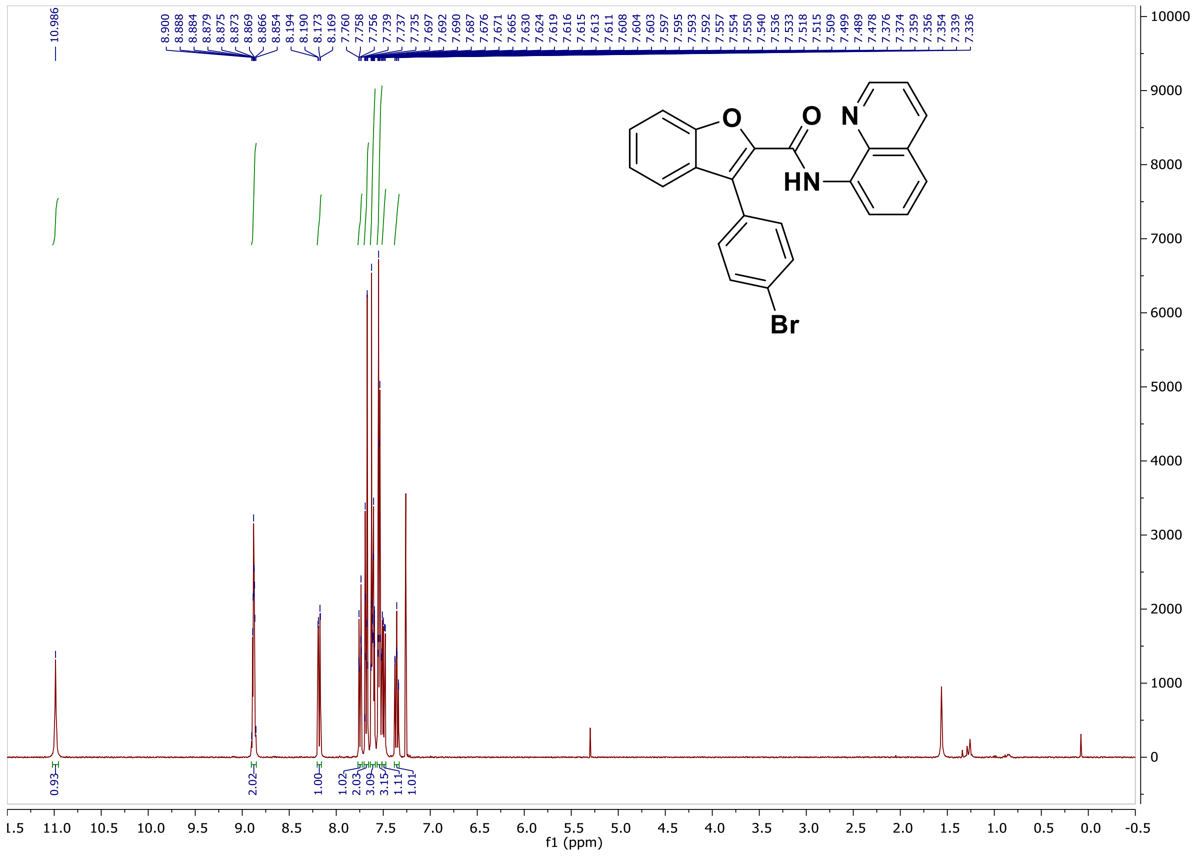

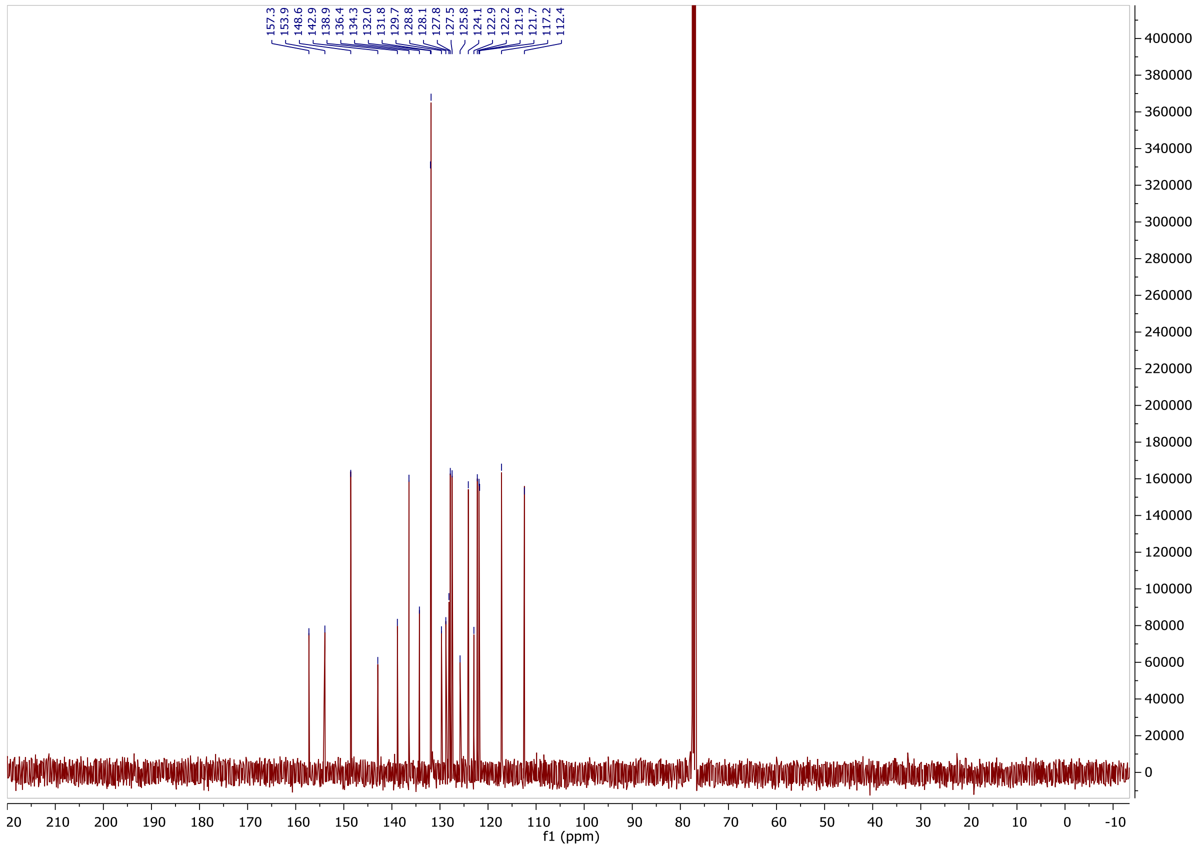

3-(4-Iodophenyl)-*N*-(quinolin-8-yl)benzofuran-2-carboxamide (**2i**)

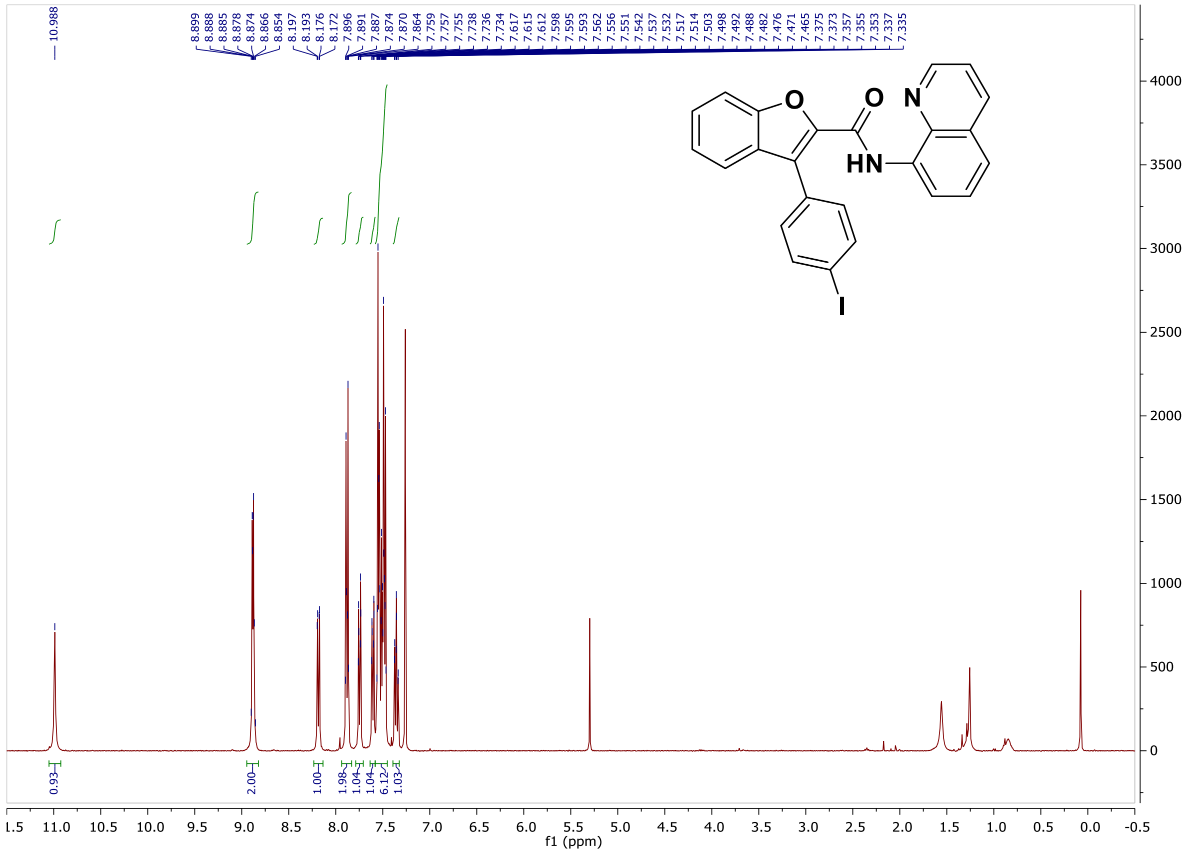

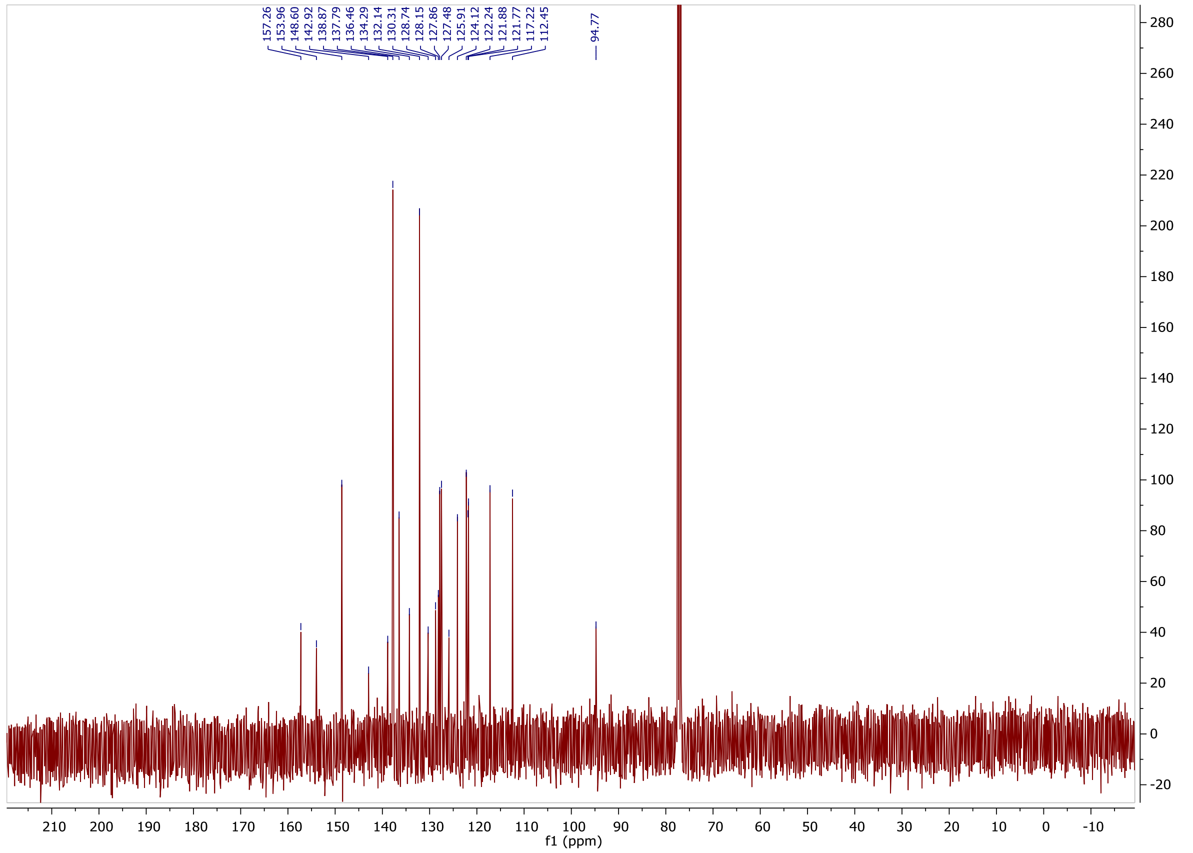

3-(4-Acetylphenyl)-*N*-(quinolin-8-yl)benzofuran-2-carboxamide (**2j**)

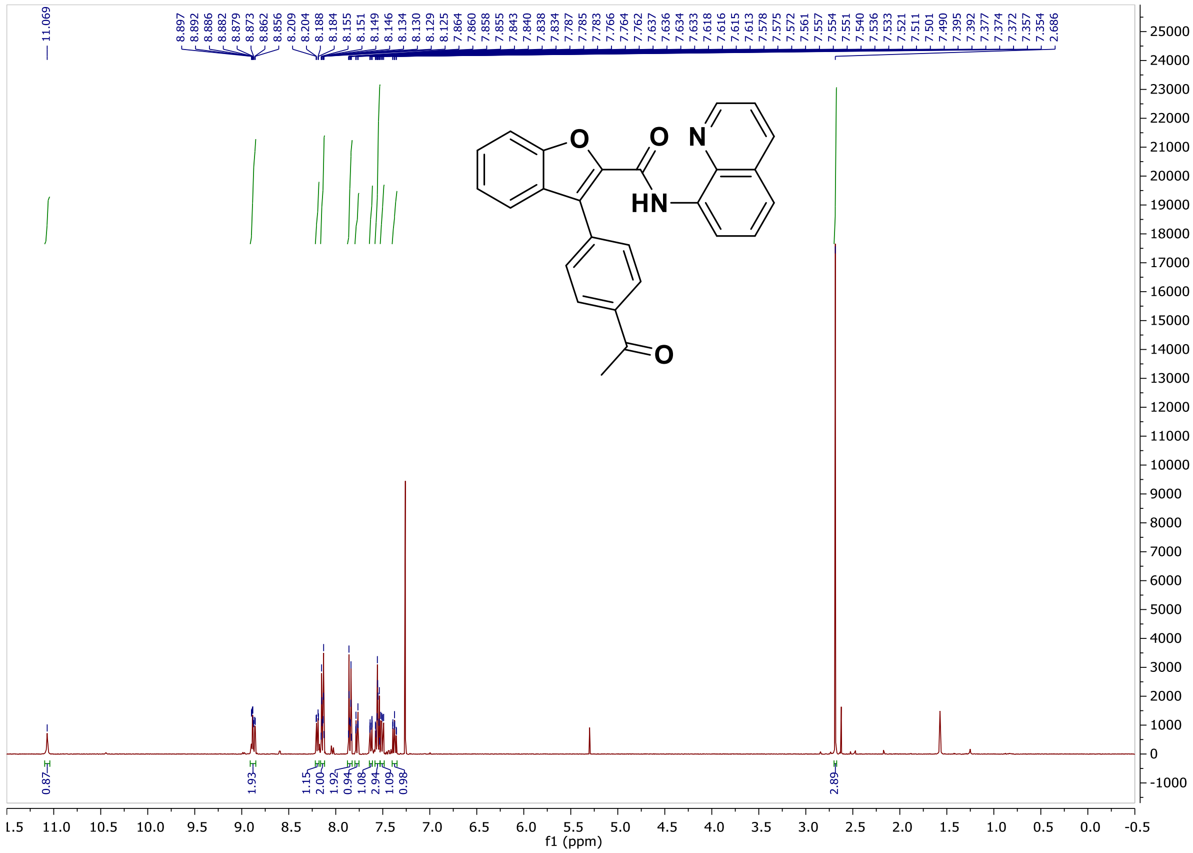

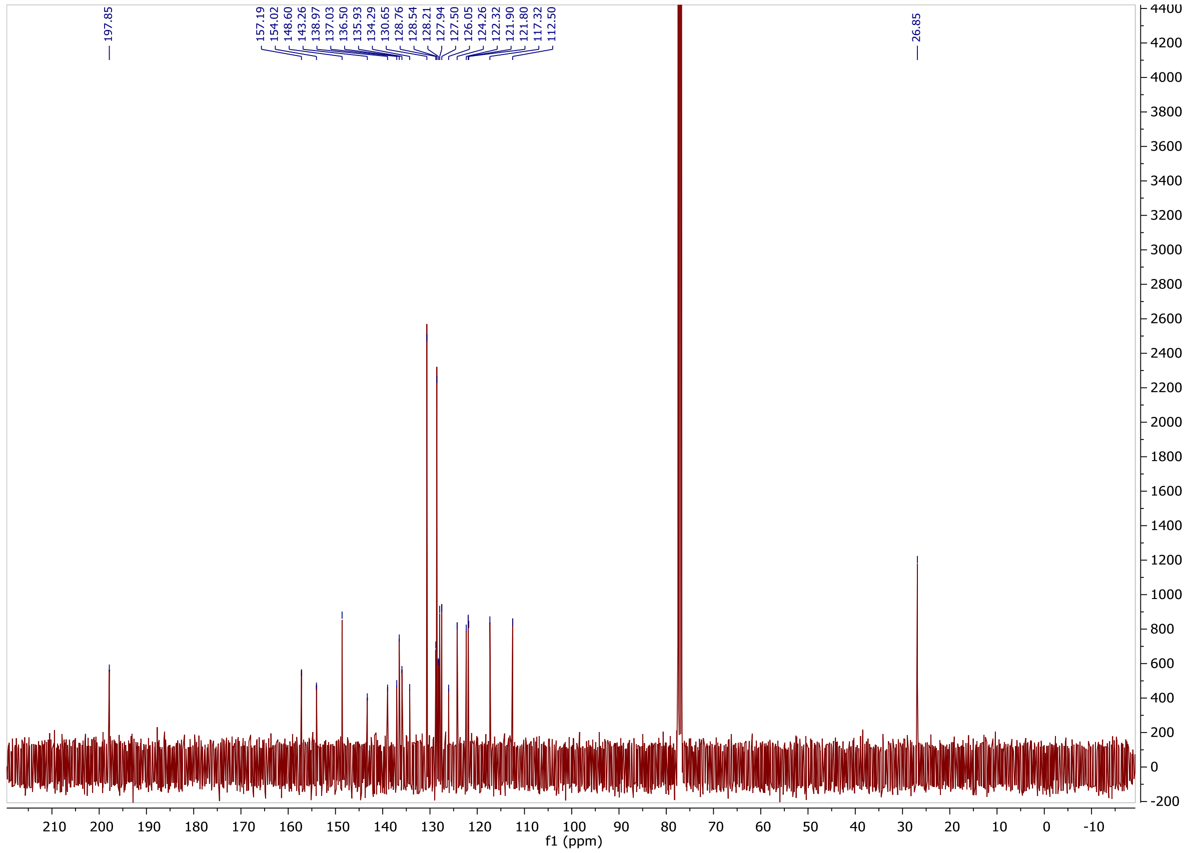

Methyl 4-(2-(quinolin-8-ylcarbamoyl)benzofuran-3-yl)benzoate (**2k**)

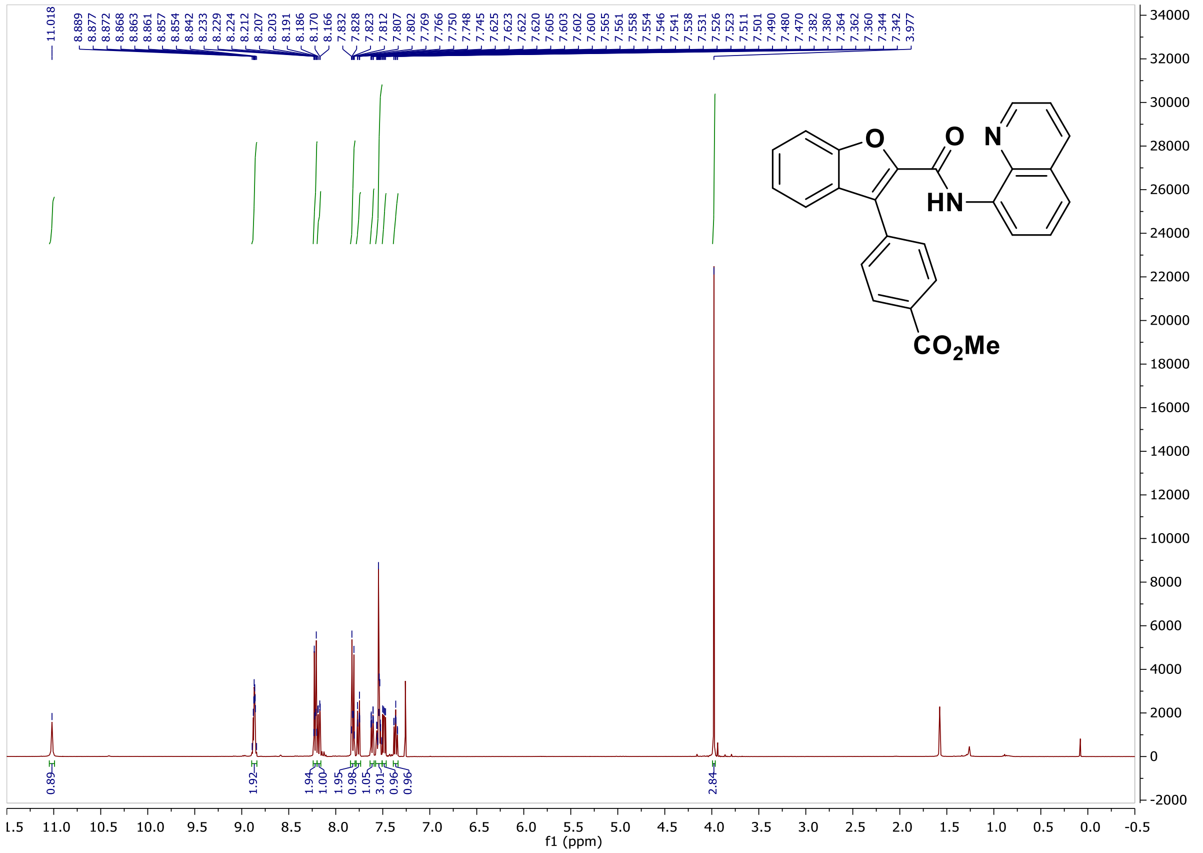

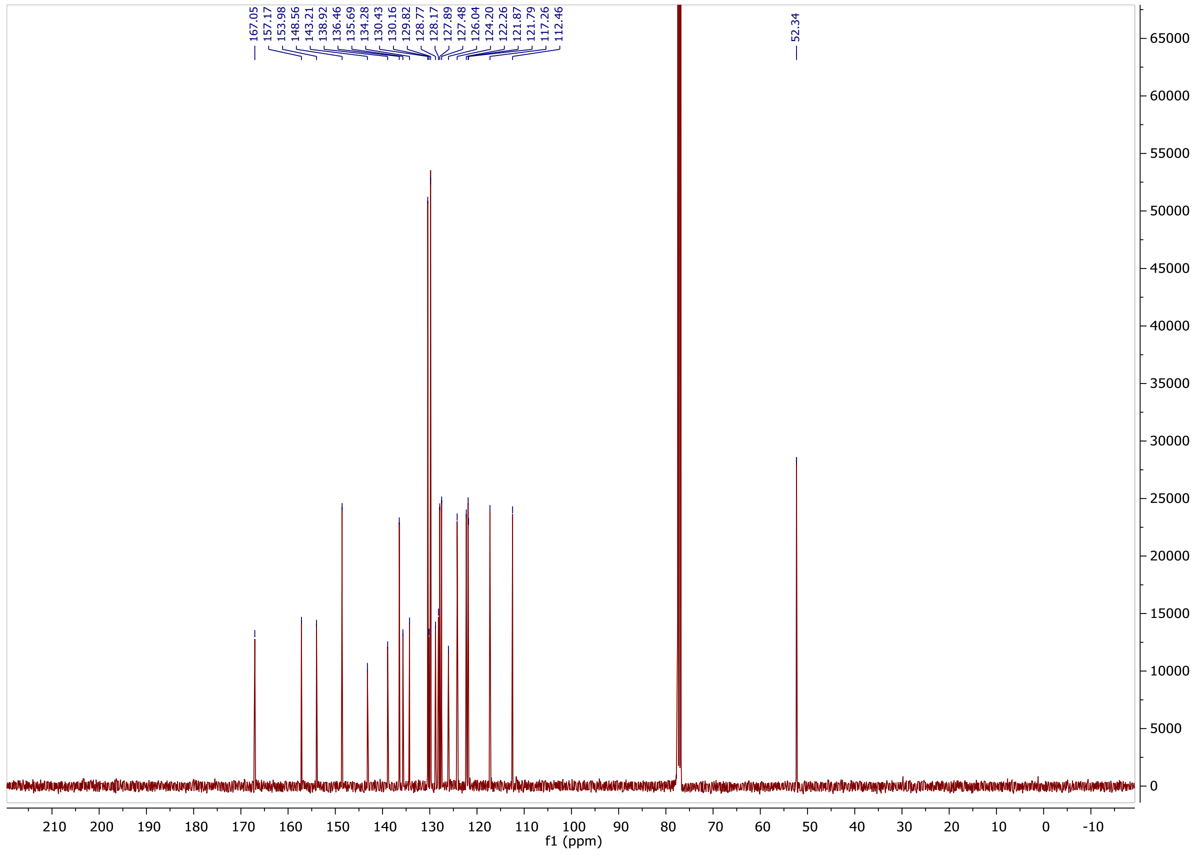

3-(4-Nitrophenyl)-*N*-(quinolin-8-yl)benzofuran-2-carboxamide (**2l**)

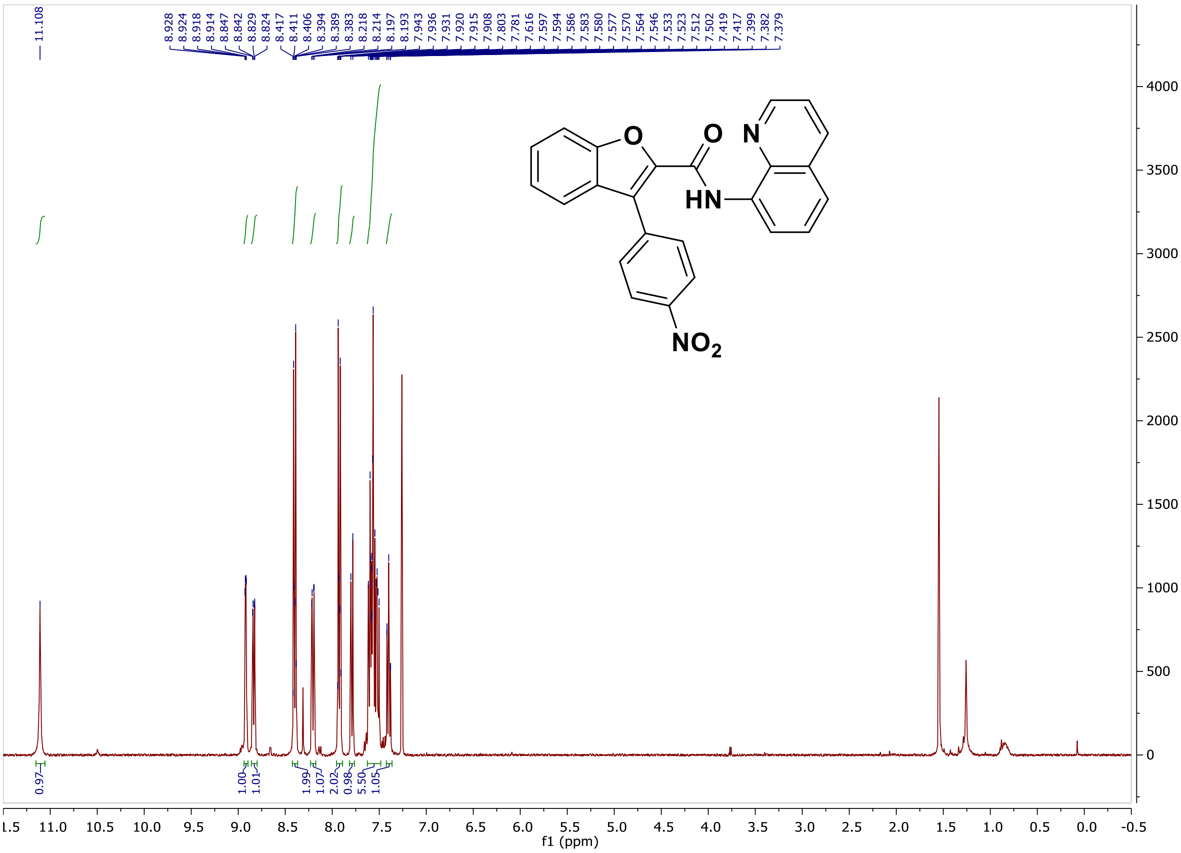

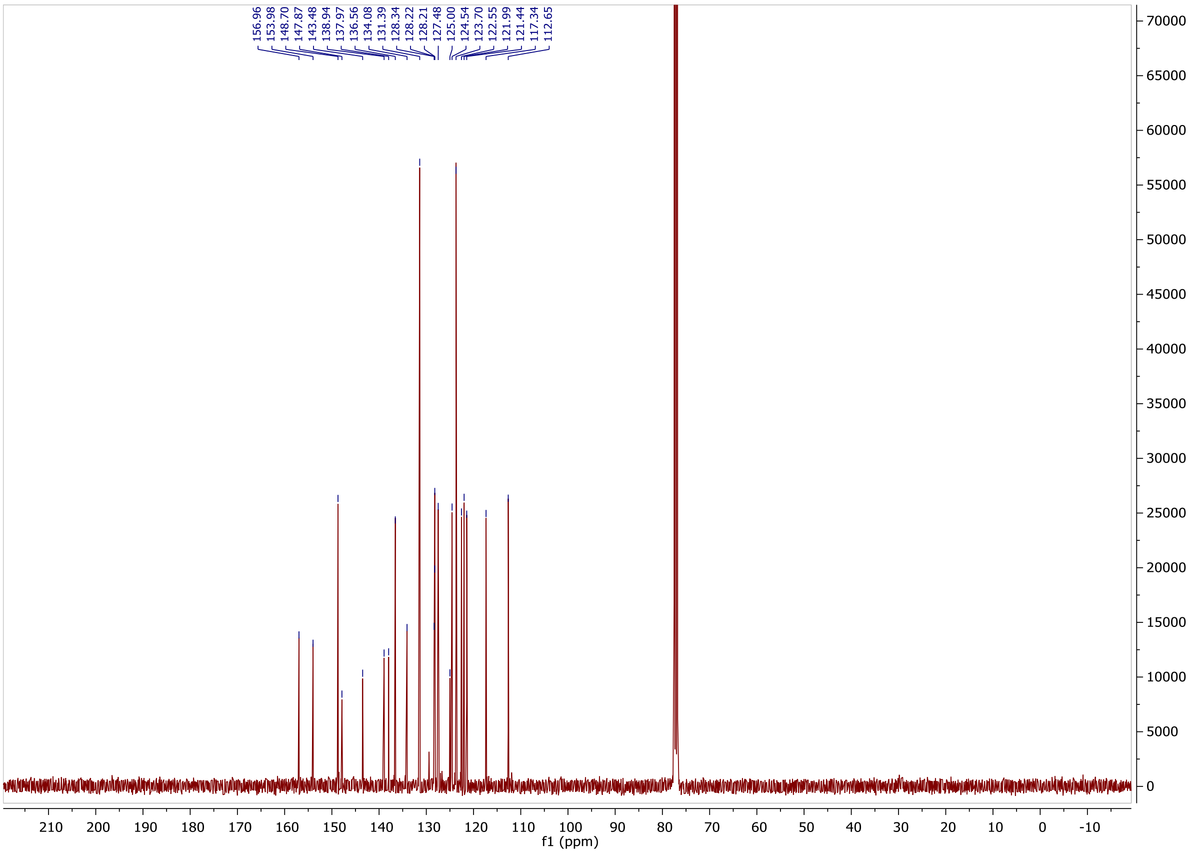

N-(Quinolin-8-yl)-3-(thiophen-2-yl)benzofuran-2-carboxamide (**2m**)

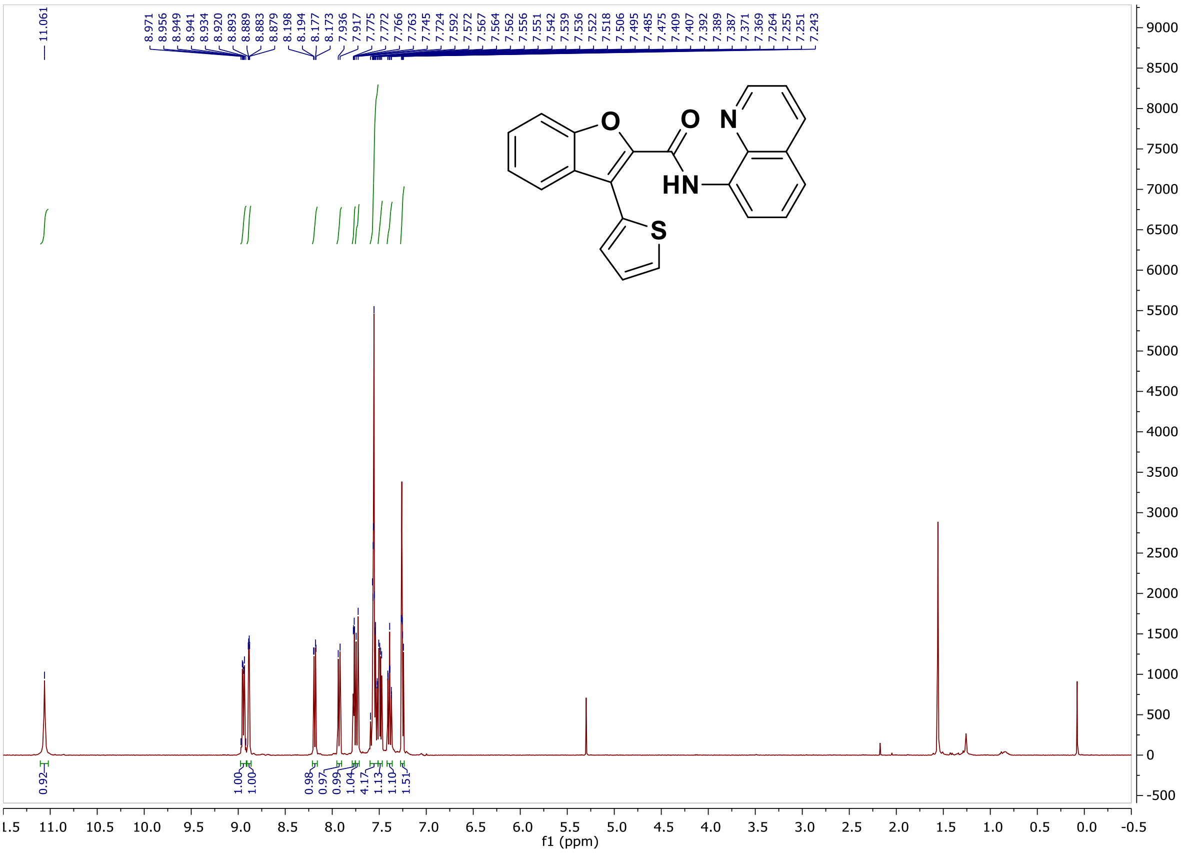

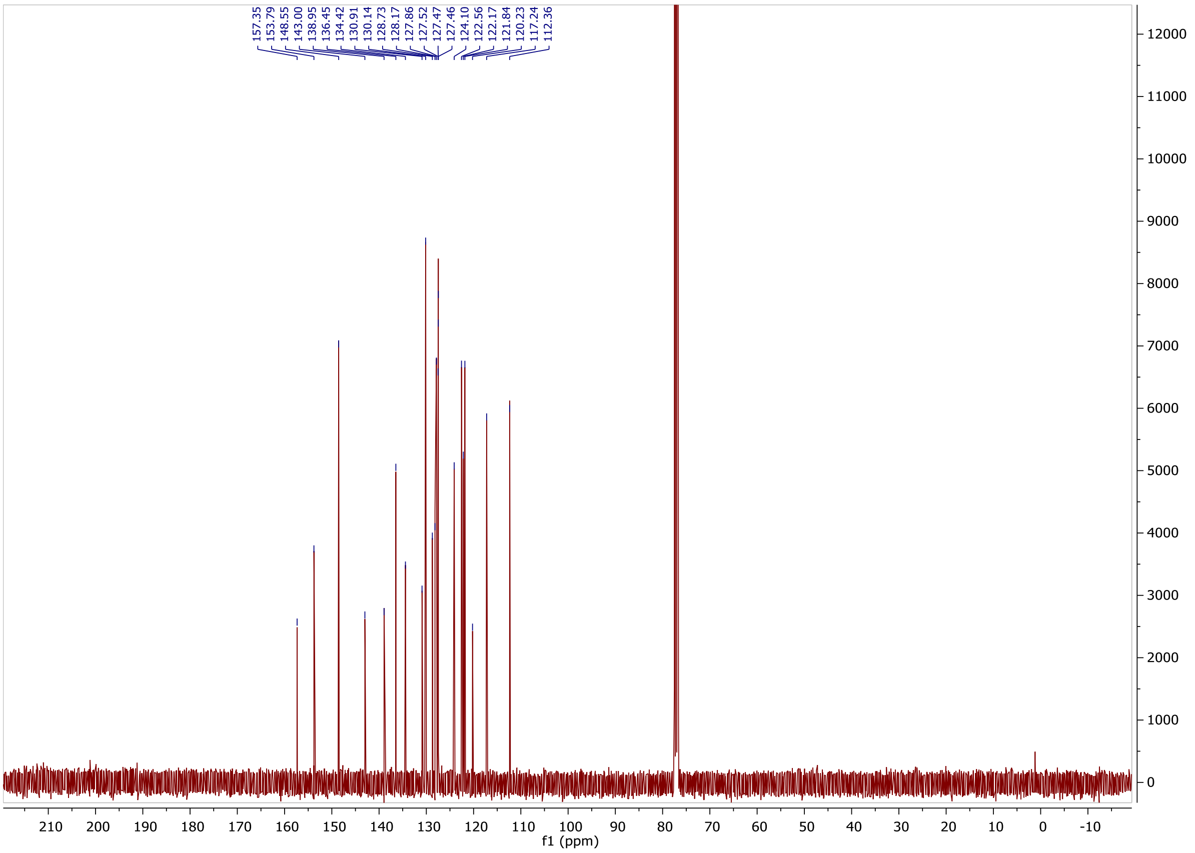

Ethyl 4-oxo-6-(2-(quinolin-8-ylcarbamoyl)benzofuran-3-yl)-4*H*-chromene-2-carboxylate (**2n**)

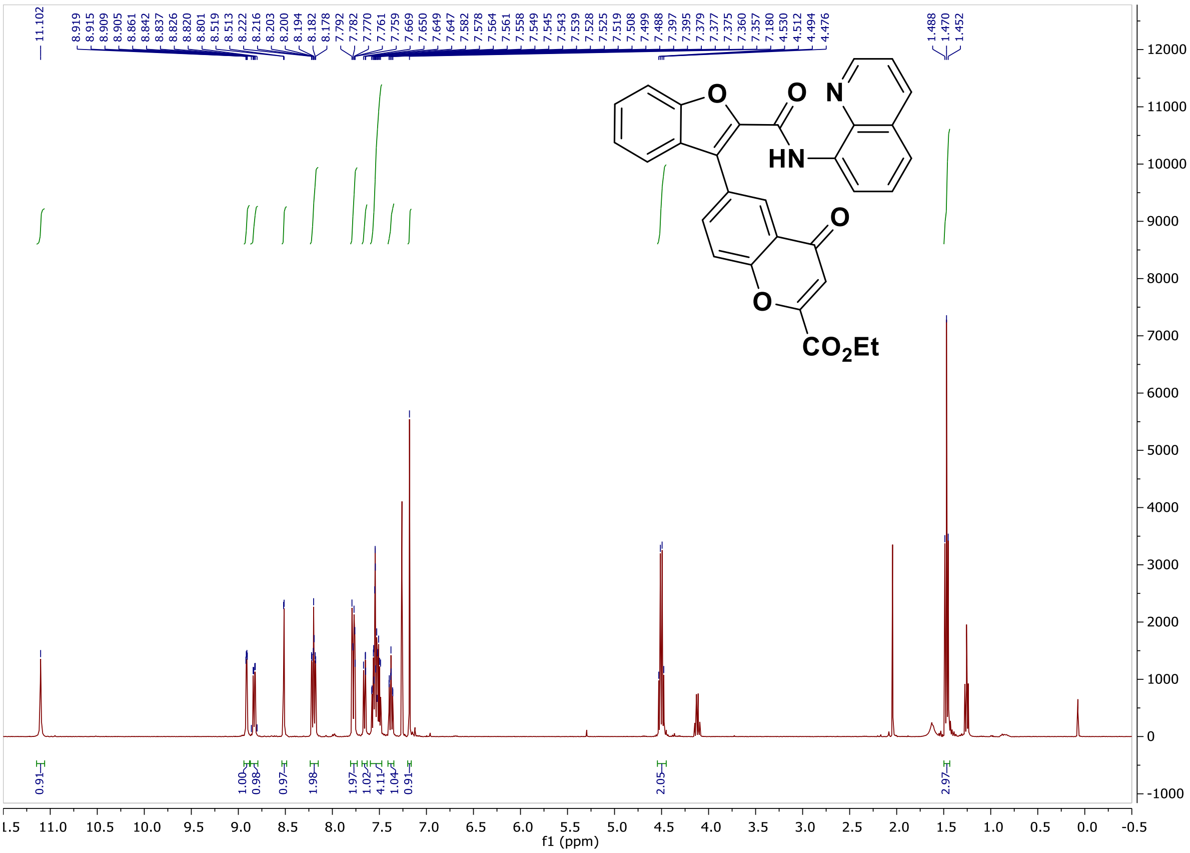

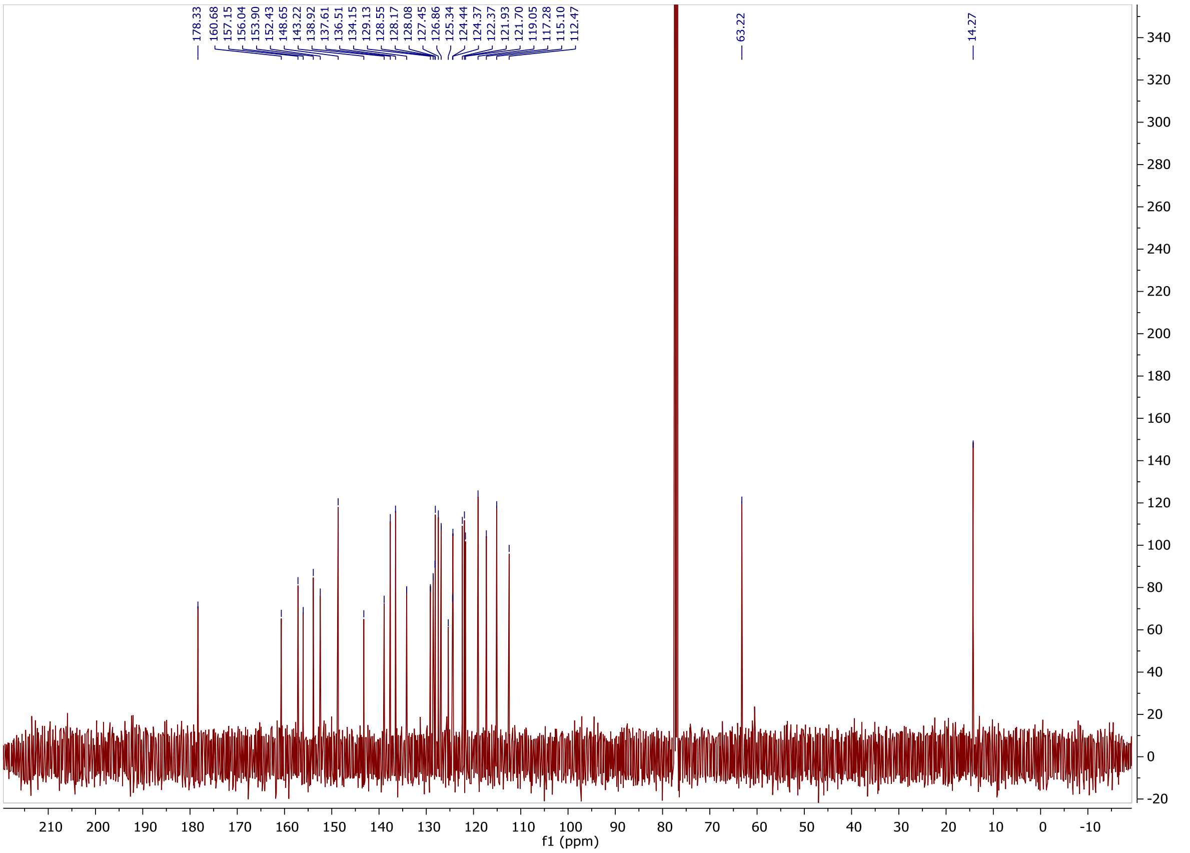

5-Methoxy-3-(4-methoxyphenyl)-*N*-(quinolin-8-yl)benzofuran-2-carboxamide (**2o**)

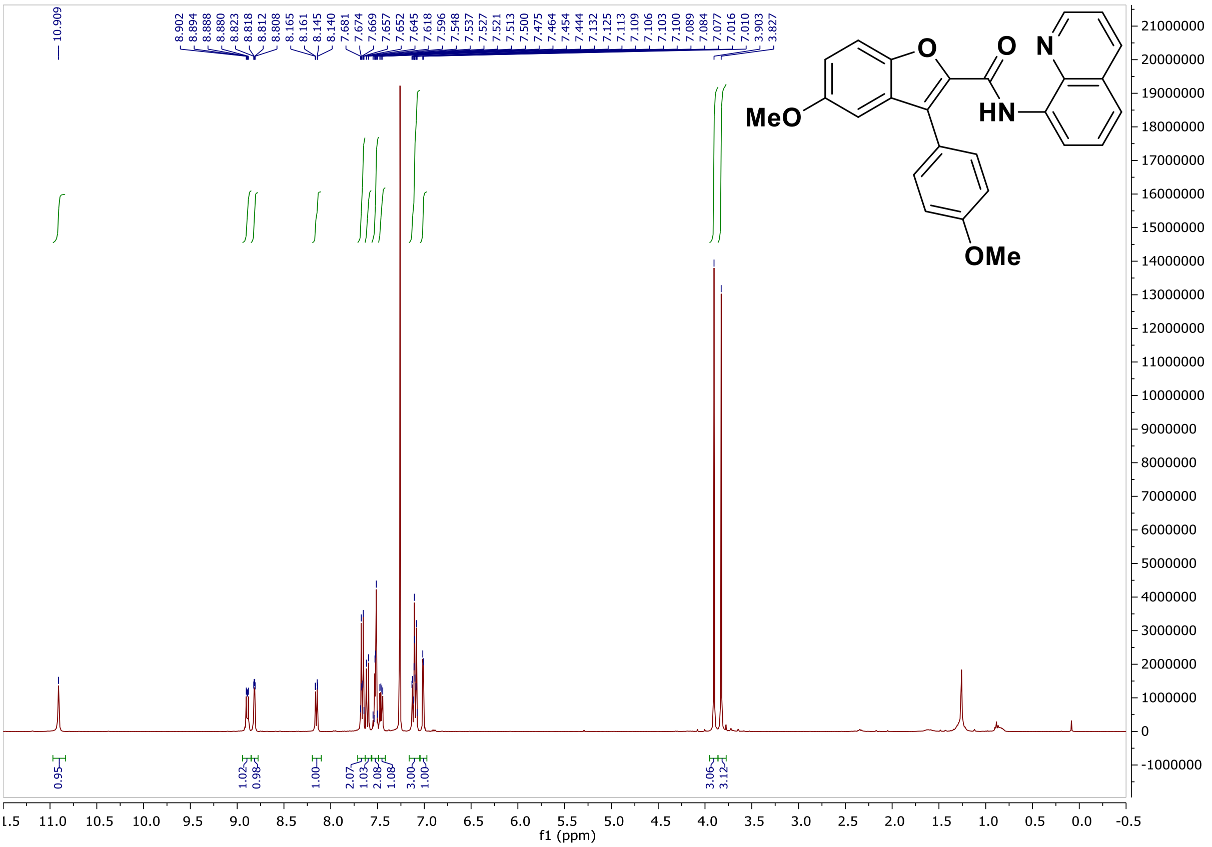

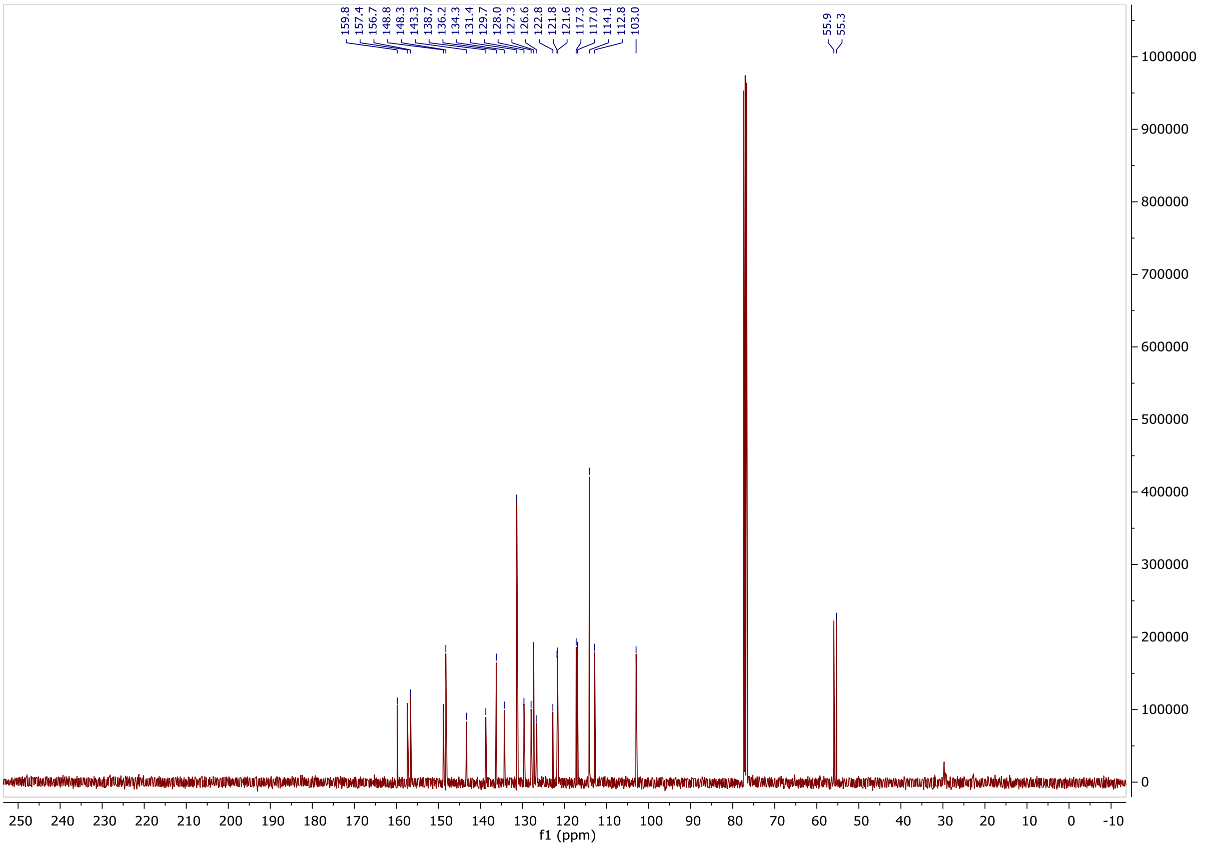

7-Methoxy-3-(4-methoxyphenyl)-*N*-(quinolin-8-yl)benzofuran-2-carboxamide (**2p**)

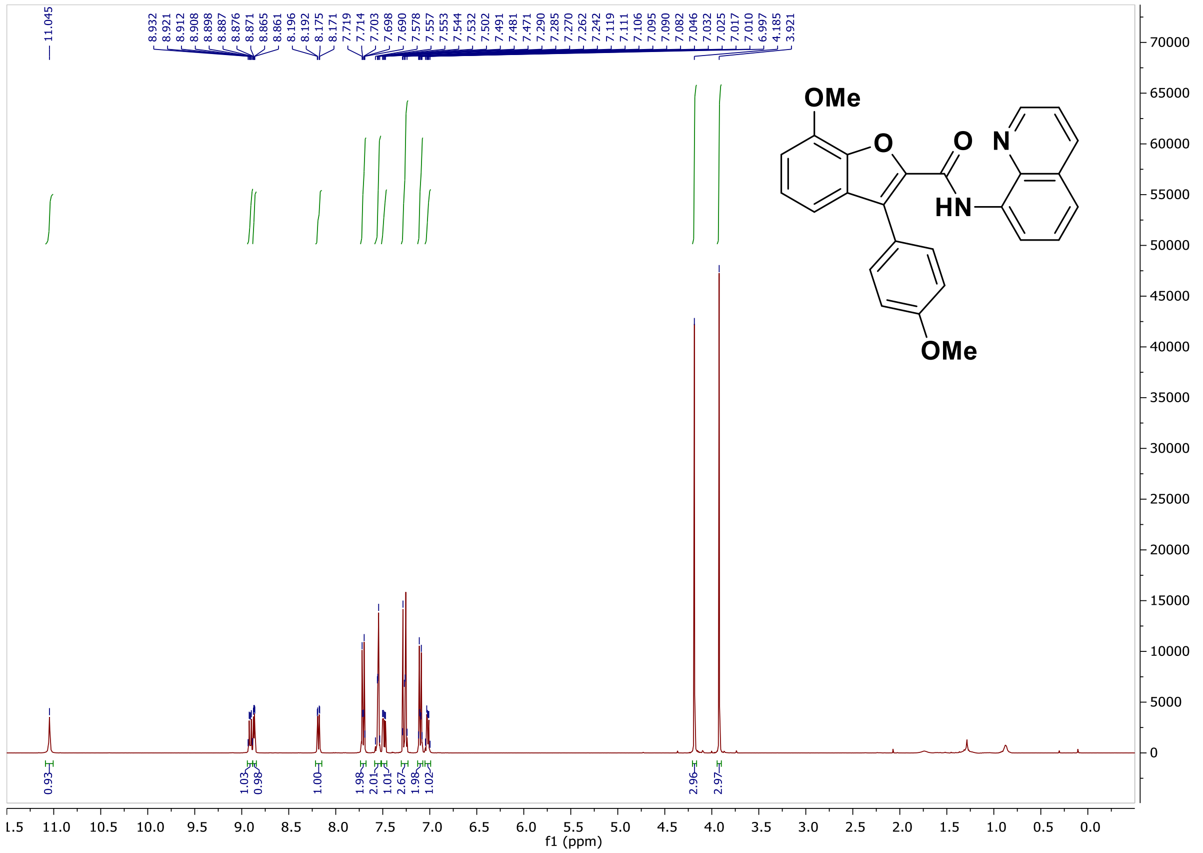

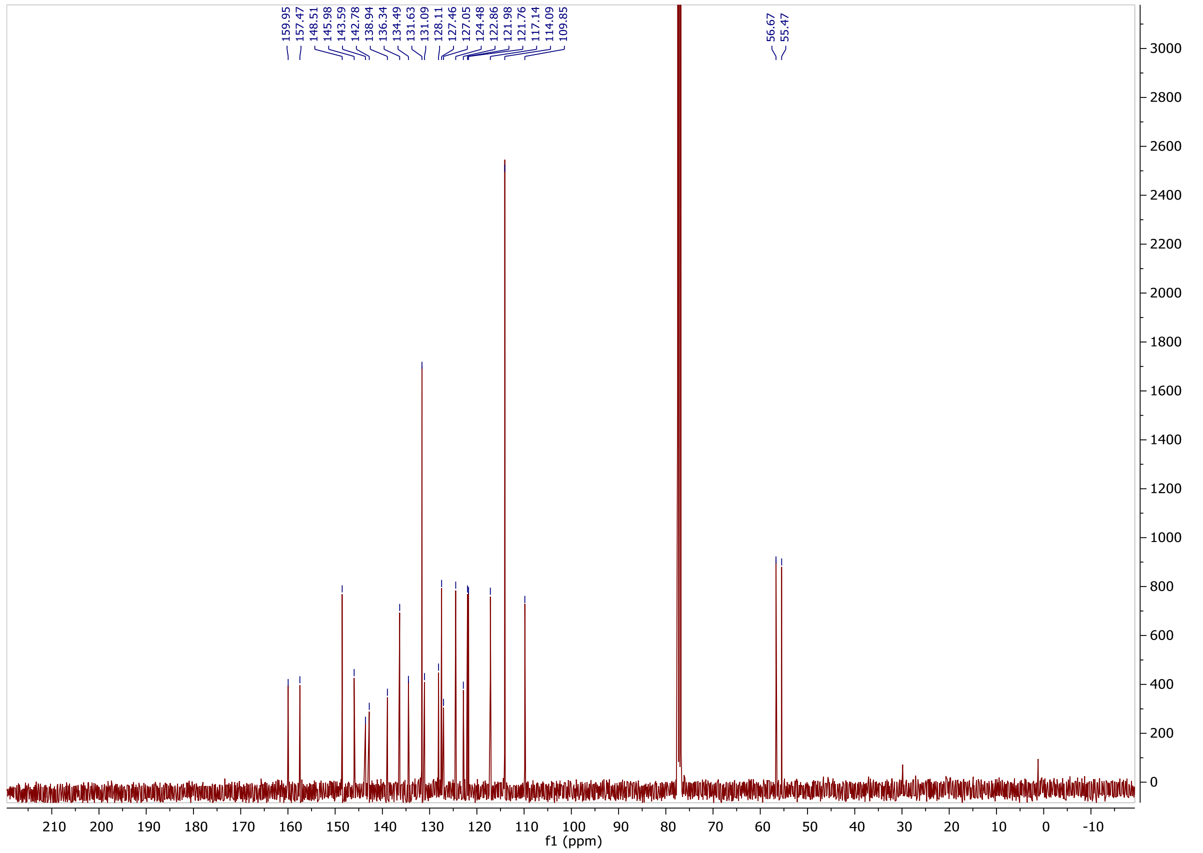

5-Chloro-3-(4-methoxyphenyl)-*N*-(quinolin-8-yl)benzofuran-2-carboxamide (**2p**)

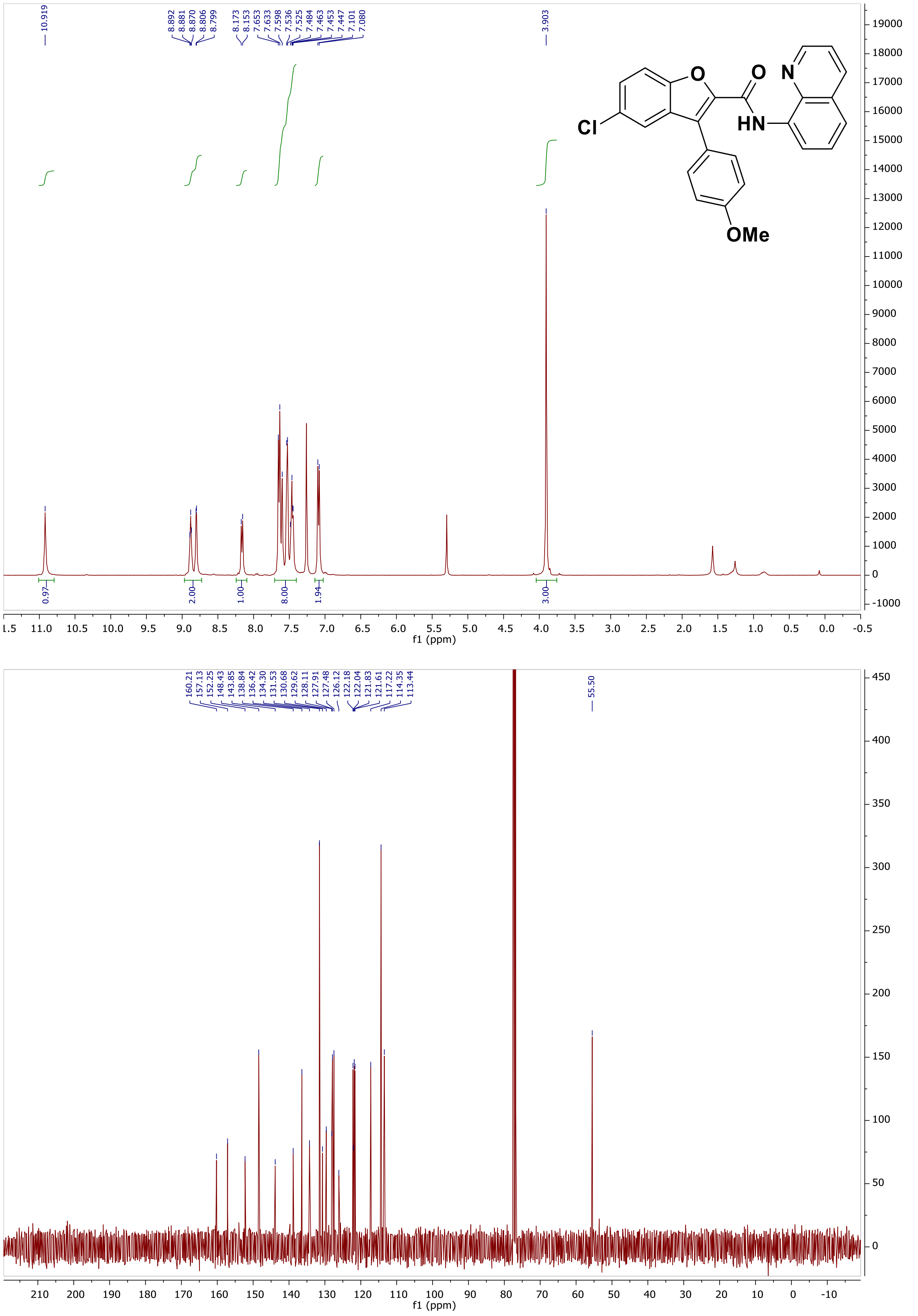

tert-Butyl (3-(4-methoxyphenyl)benzofuran-2-carbonyl)(quinolin-8-yl)carbamate

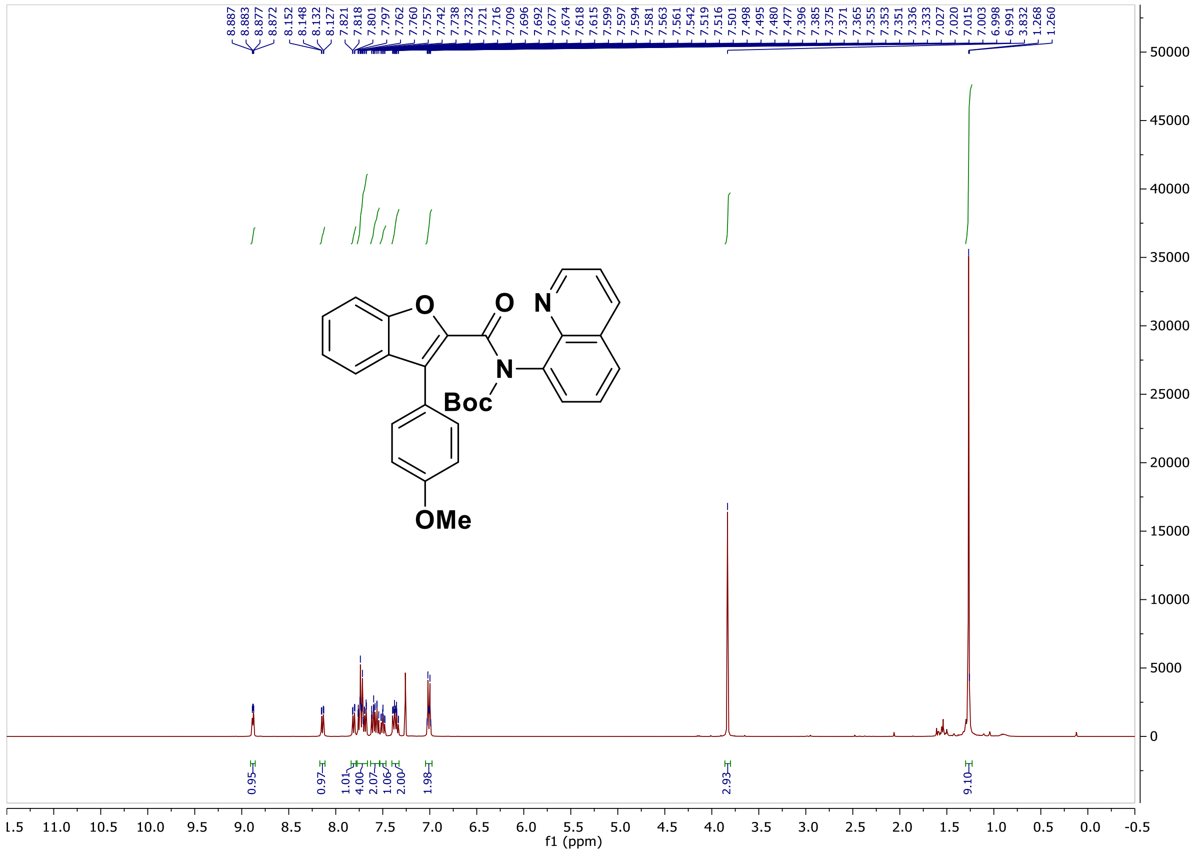

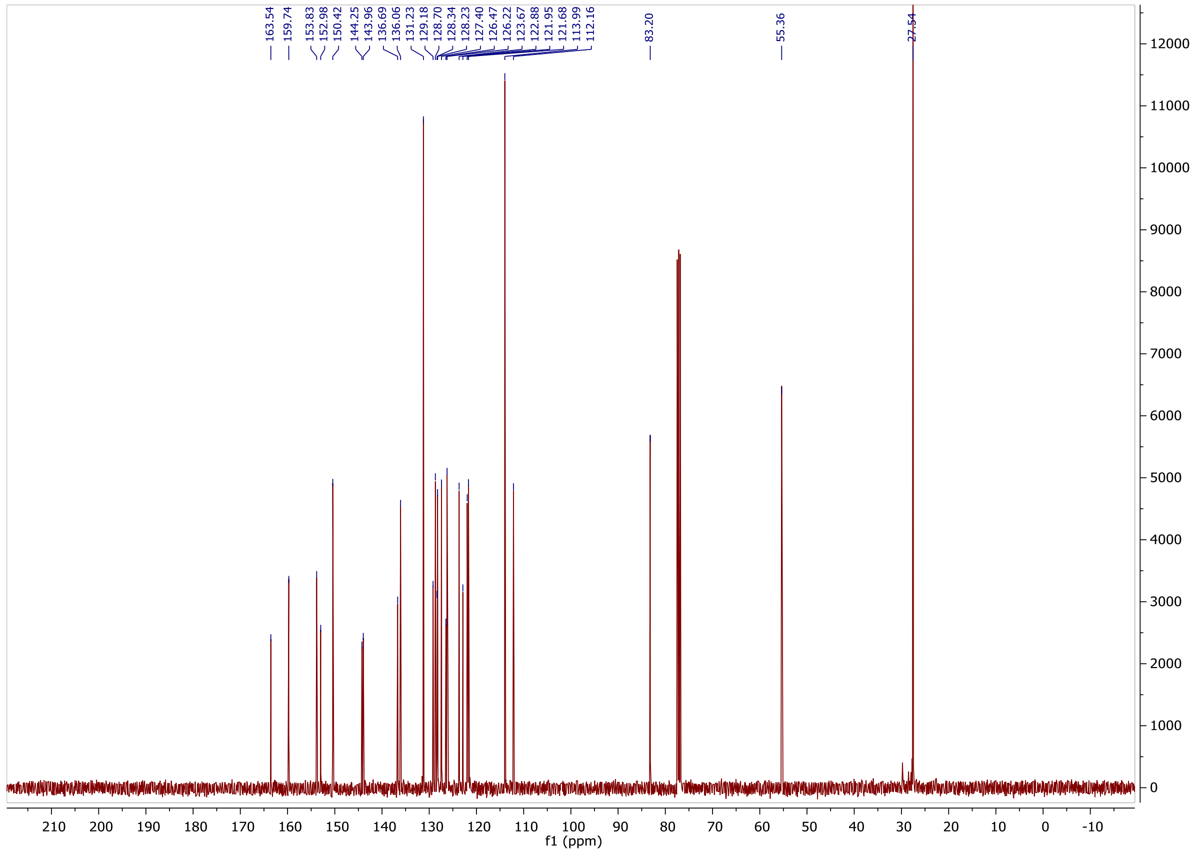

N-Benzyl-3-(4-methoxyphenyl)benzofuran-2-carboxamide (**3a**)

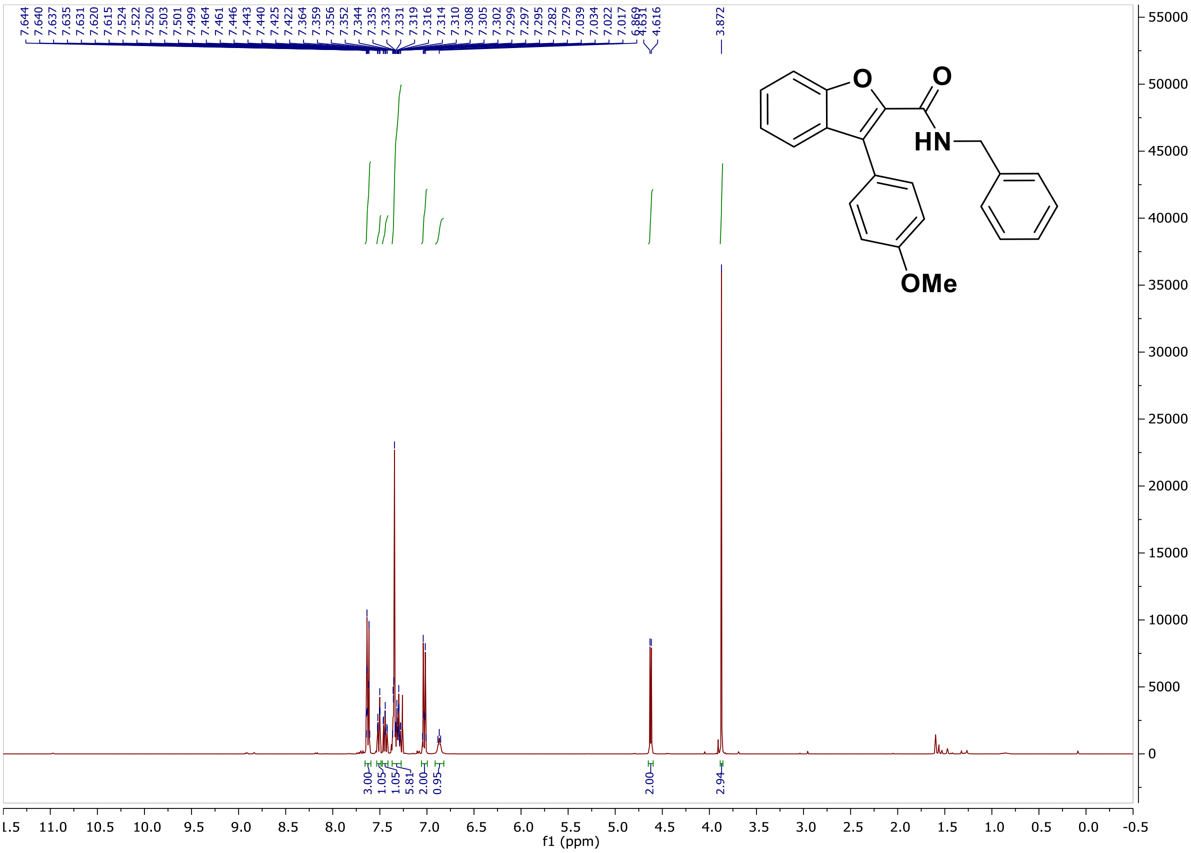

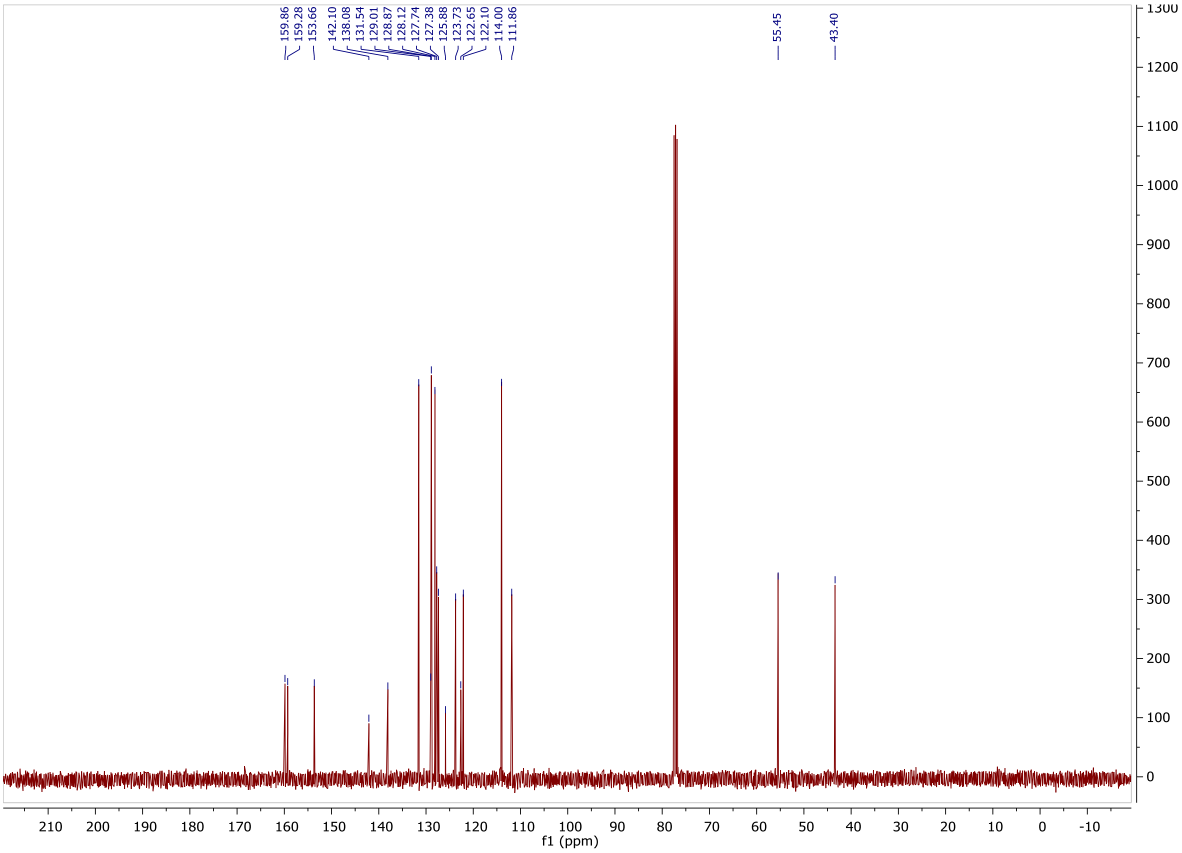

3-(4-Methoxyphenyl)-*N*-(pyridin-2-ylmethyl)benzofuran-2-carboxamide (**3b**)

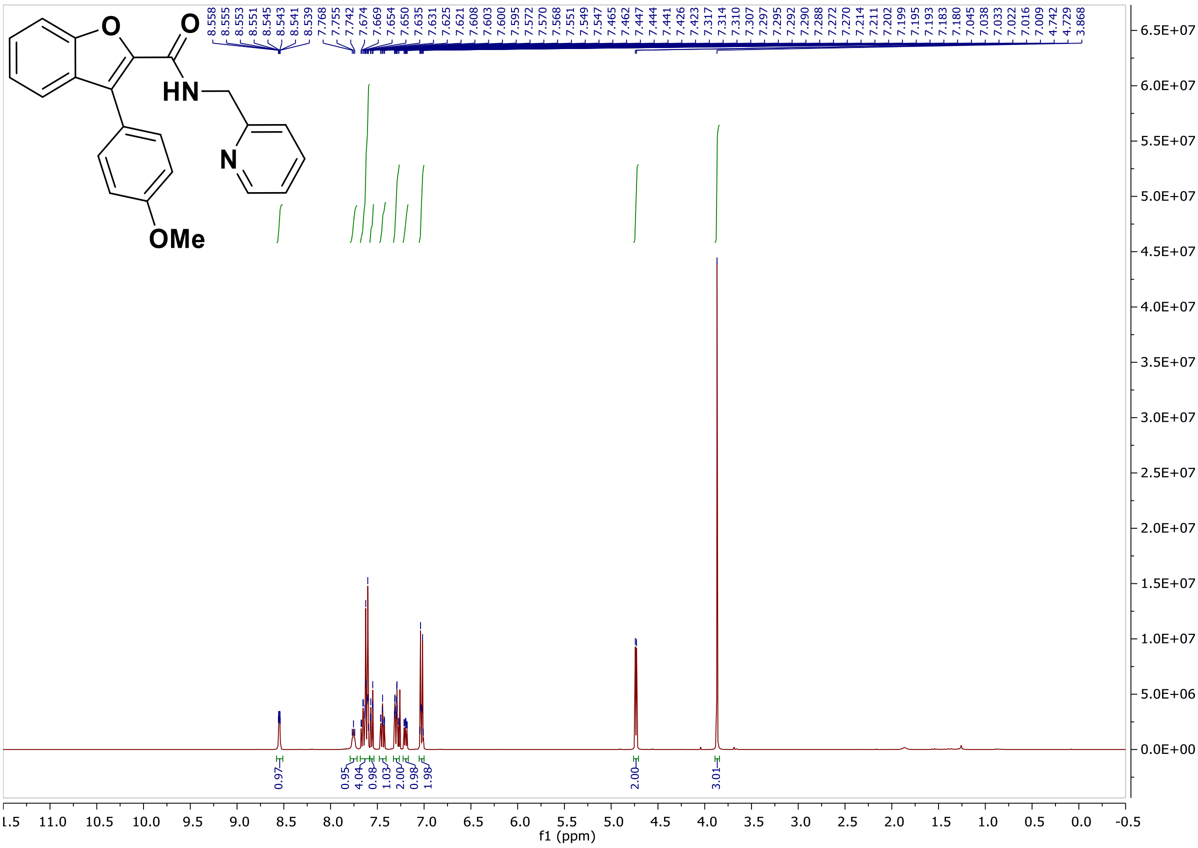

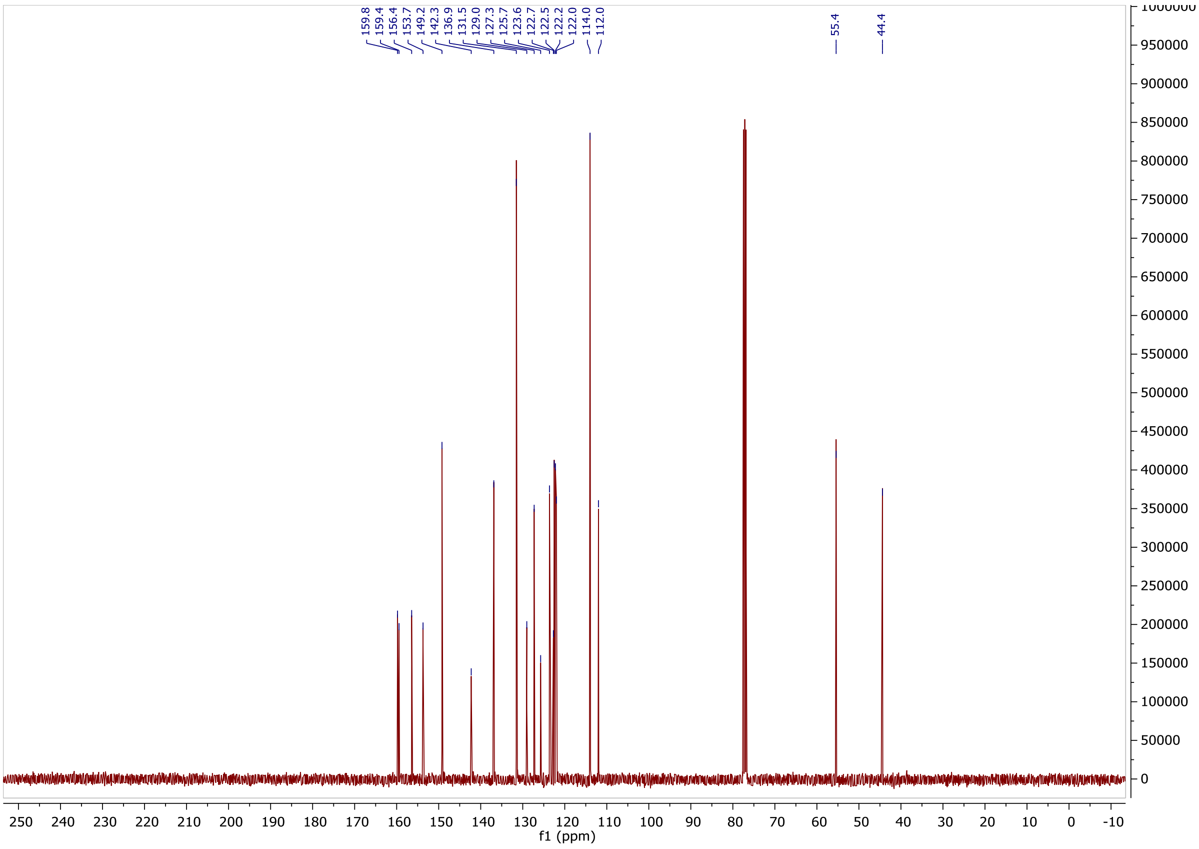

N-(4-Hydroxybenzyl)-3-(4-methoxyphenyl)benzofuran-2-carboxamide (**3c**)

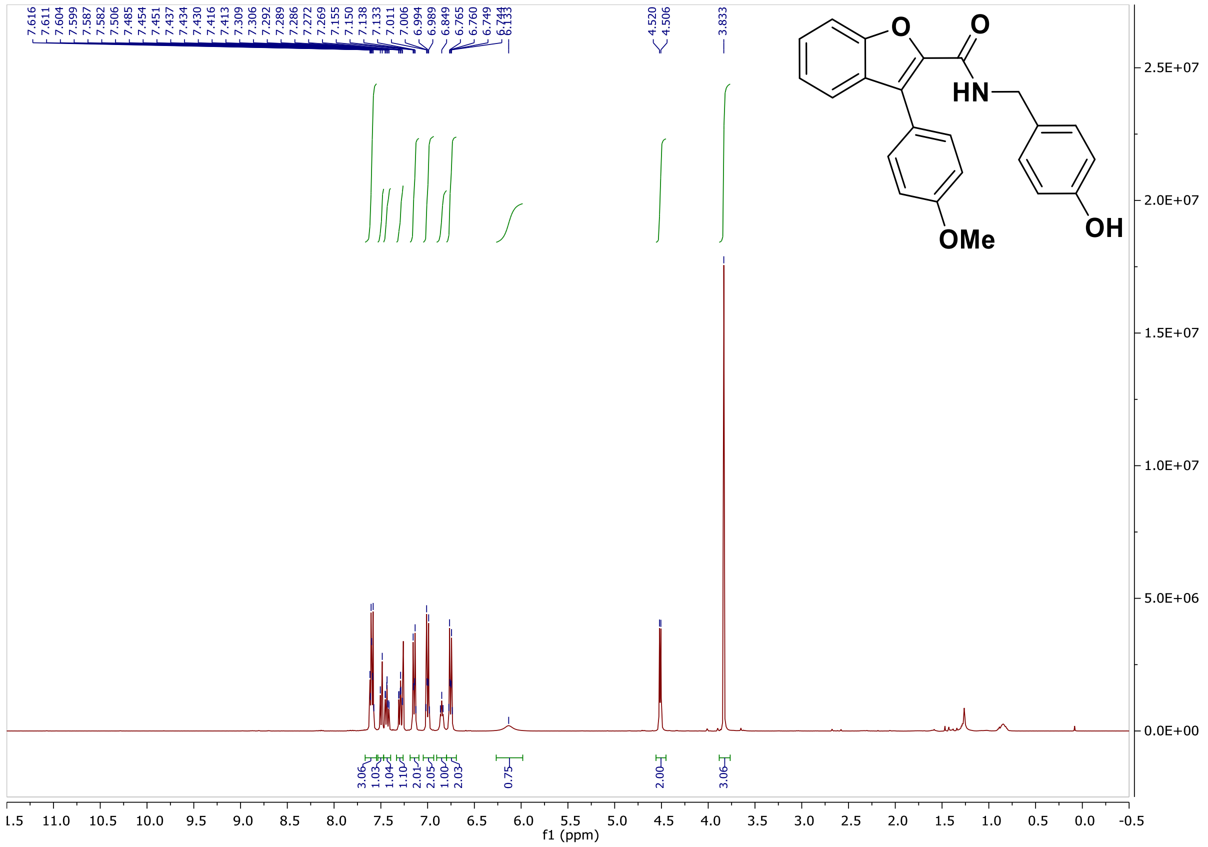

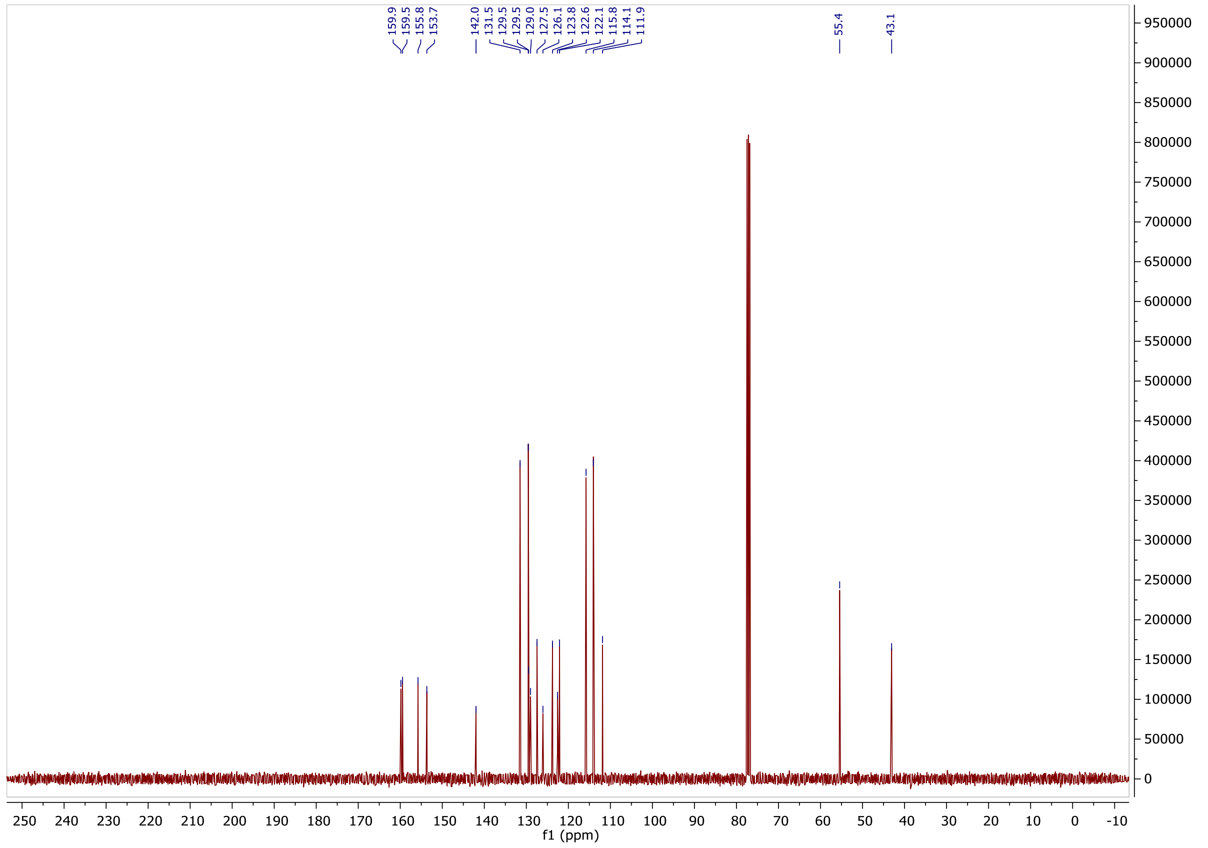

N-(Benzo[*d*][1,3]dioxol-5-ylmethyl)-3-(4-methoxyphenyl)benzofuran-2-carboxamide (**3d**)

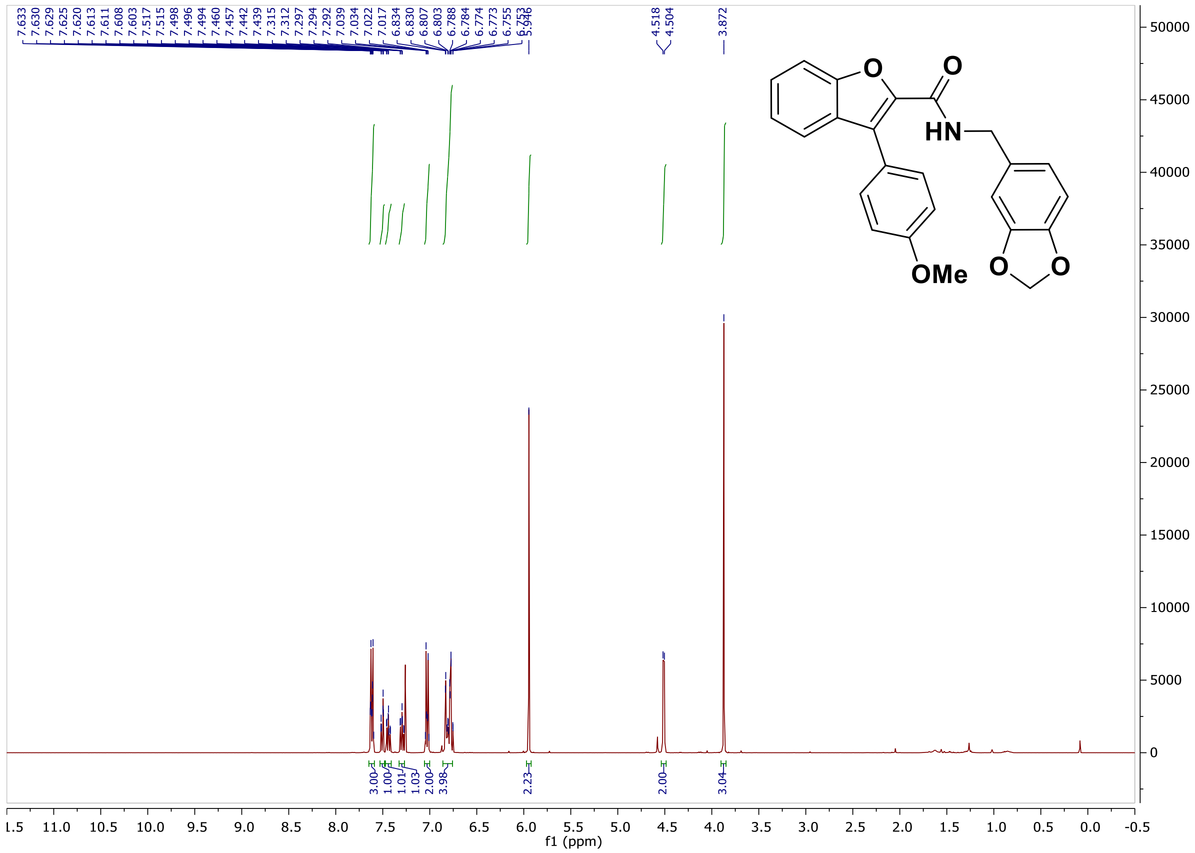

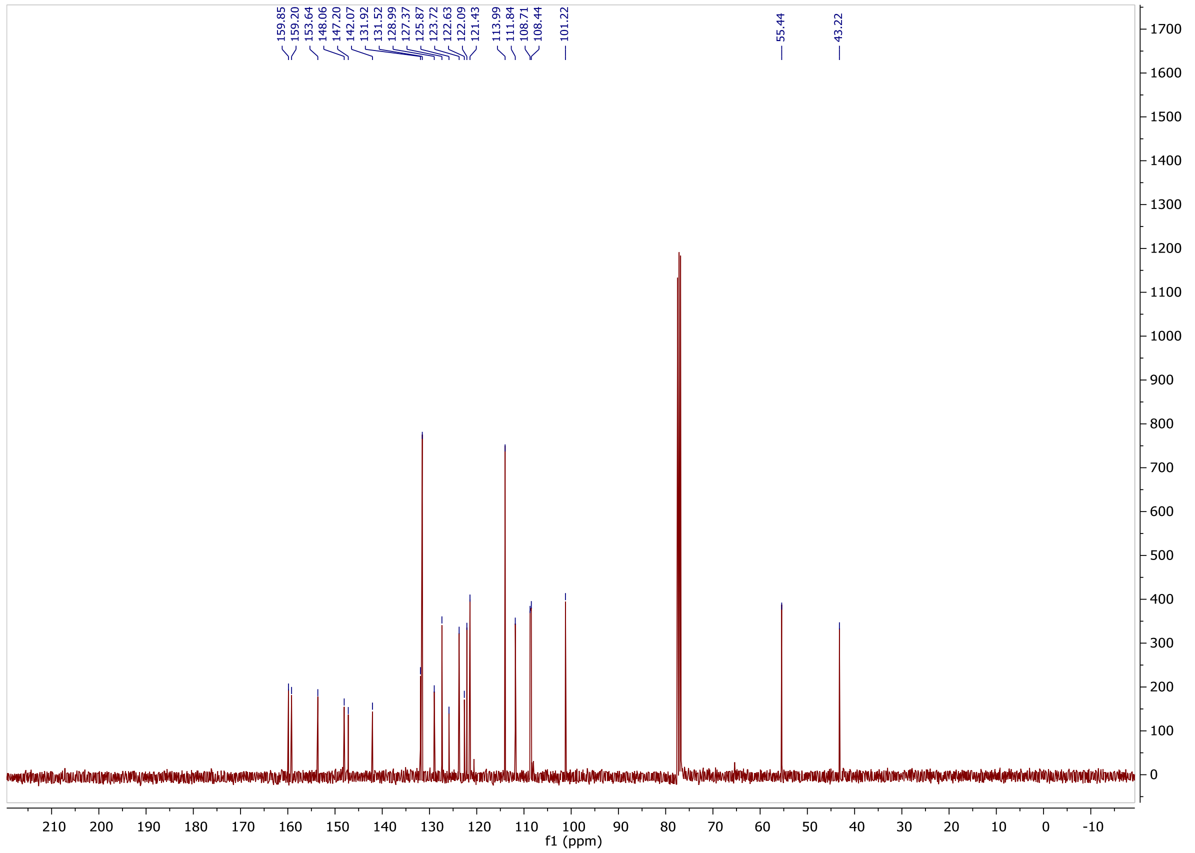

N-(2-(1*H*-Indol-3-yl)ethyl)-3-(4-methoxyphenyl)benzofuran-2-carboxamide (**3e**)

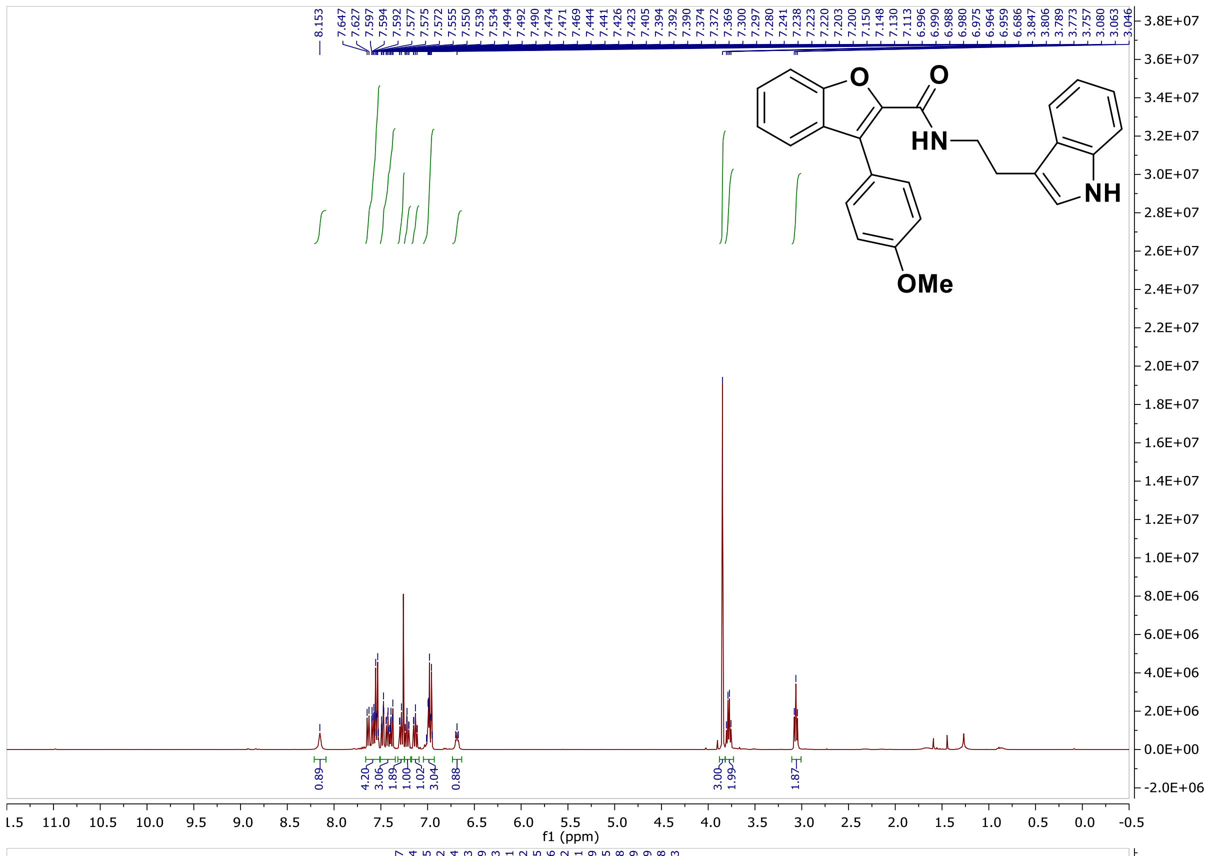

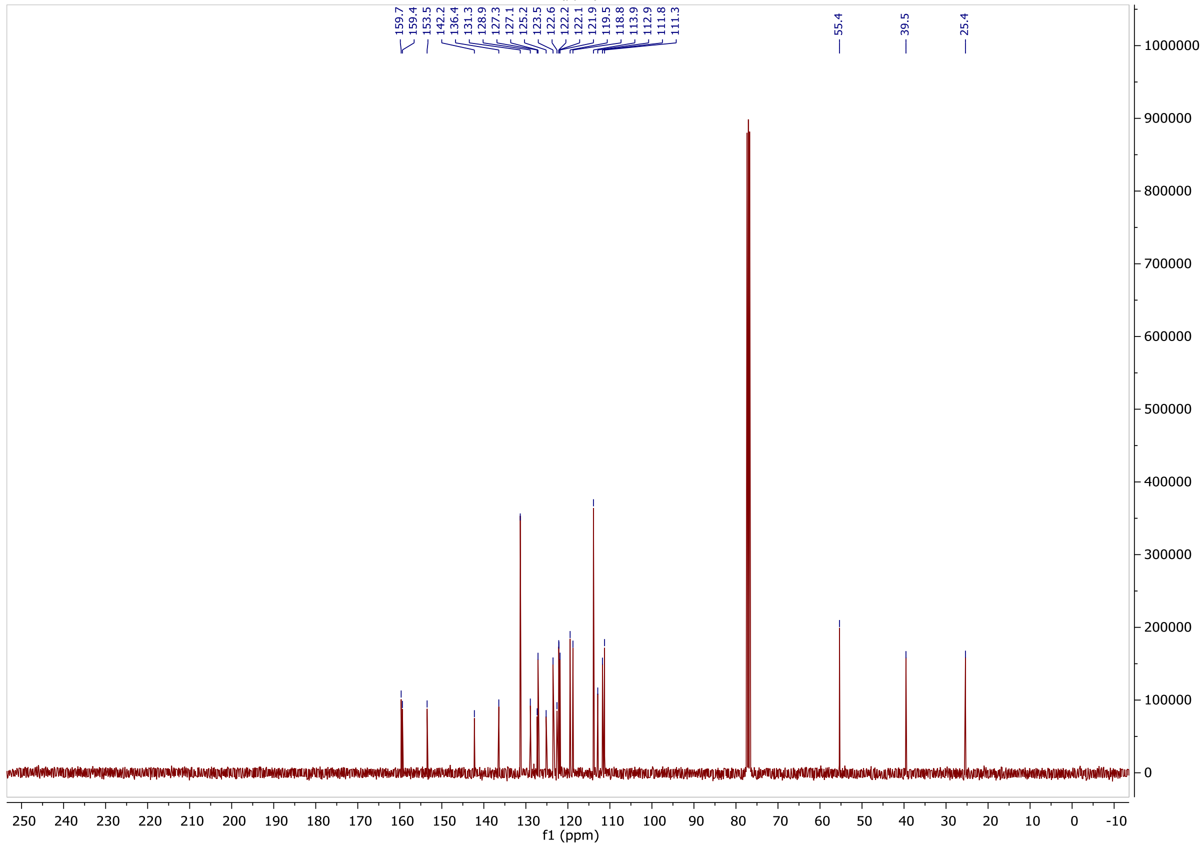

(3-(4-Bethoxyphenyl)benzofuran-2-yl)(pyrrolidin-1-yl)methanone (**3f**)

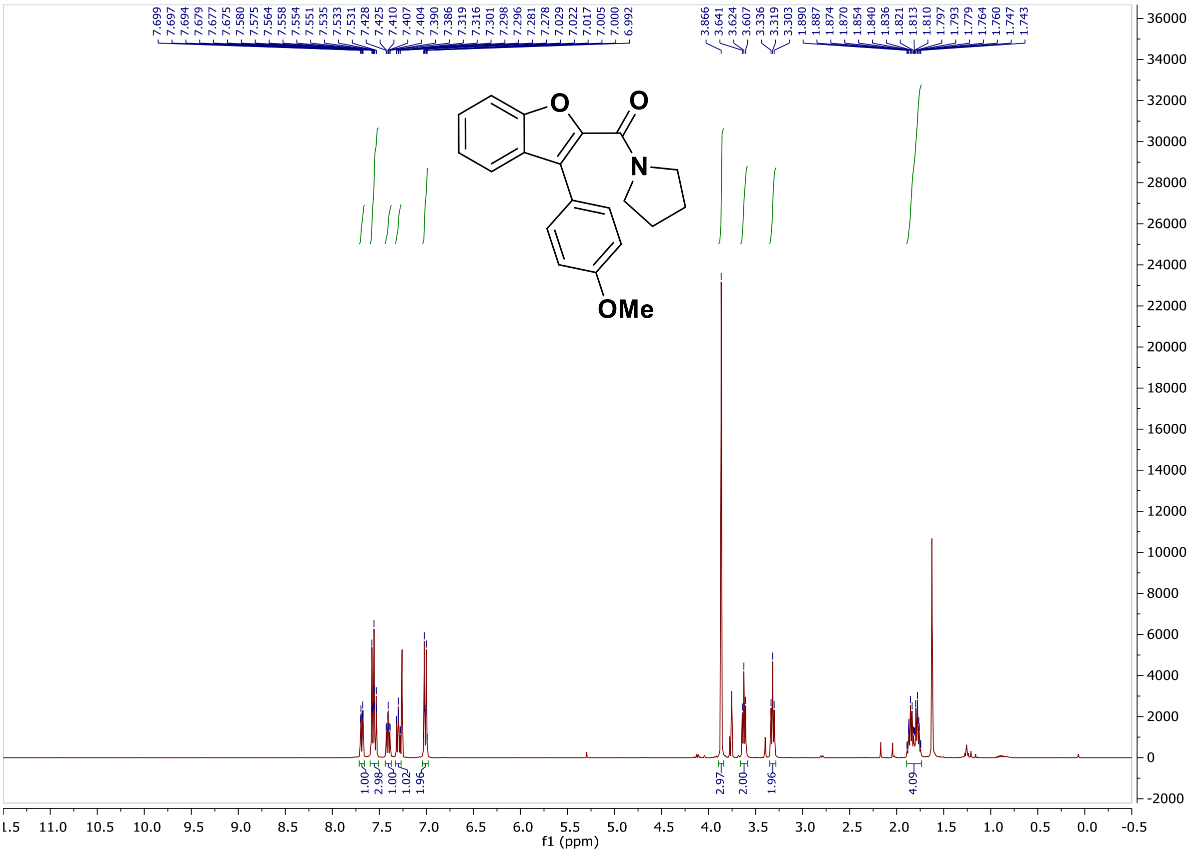

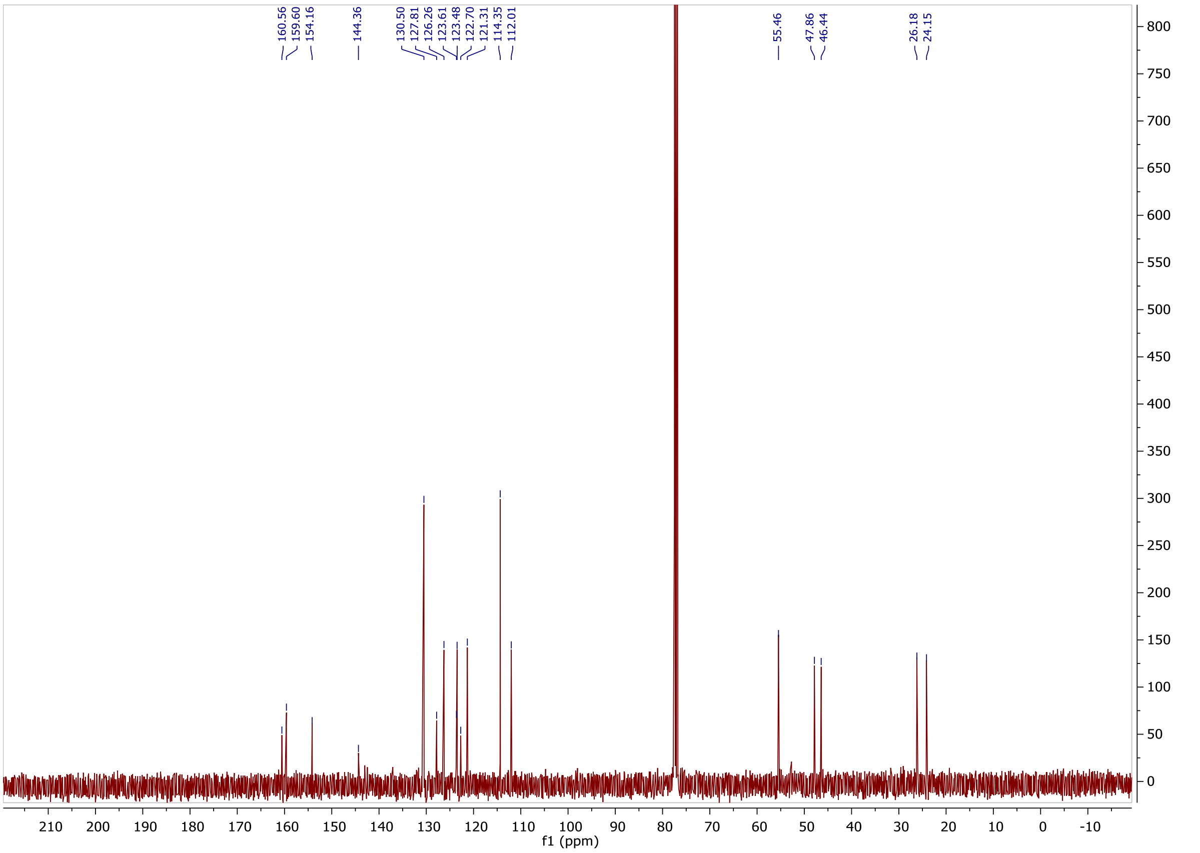

(3-(4-Methoxyphenyl)benzofuran-2-yl)(piperidin-1-yl)methanone (**3g**)

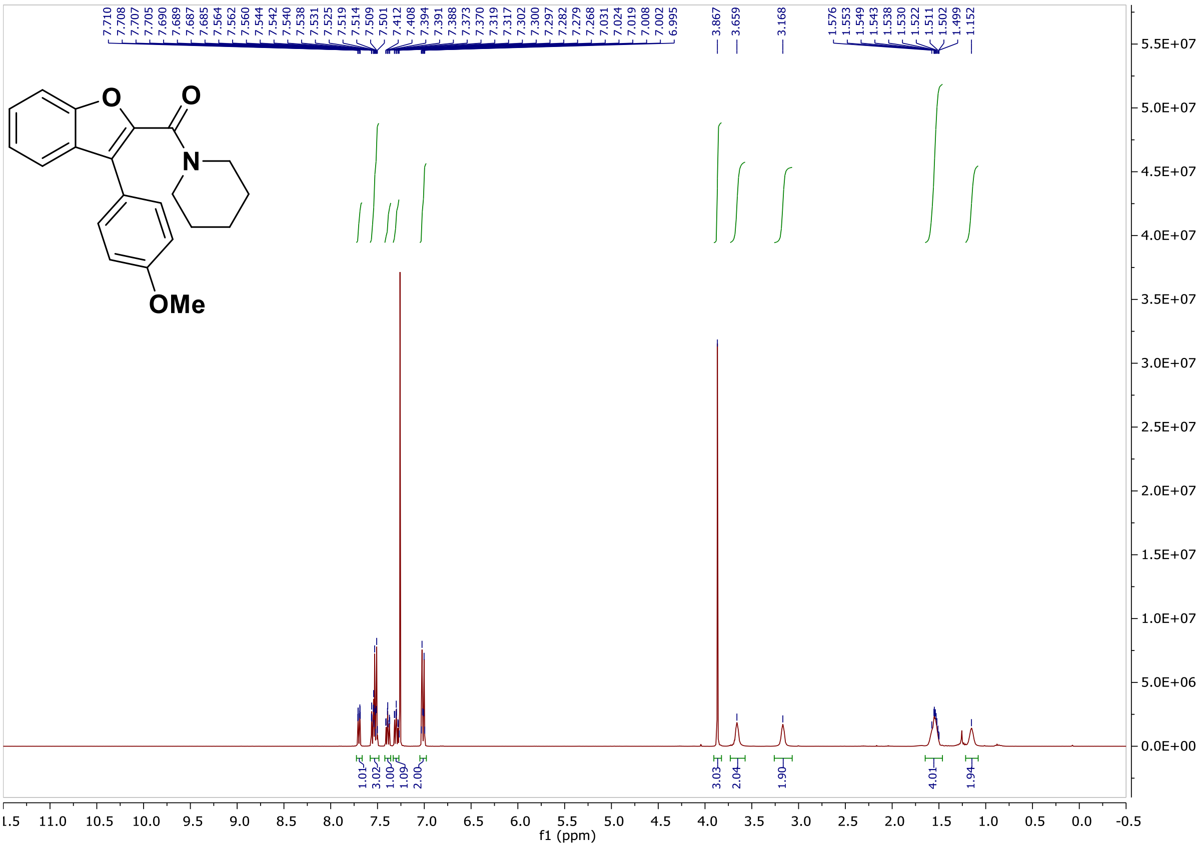

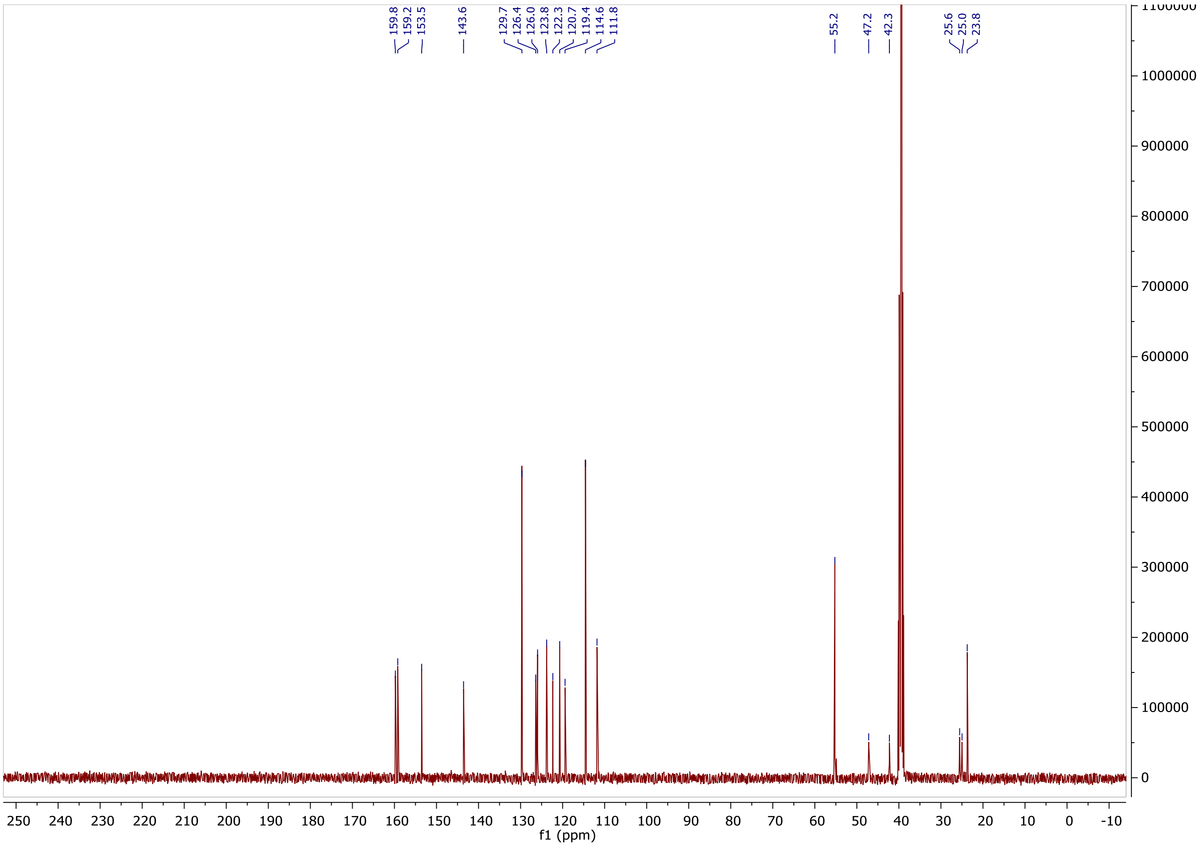

(3-(4-Methoxyphenyl)benzofuran-2-yl)(morpholino)methanone (**3h**)

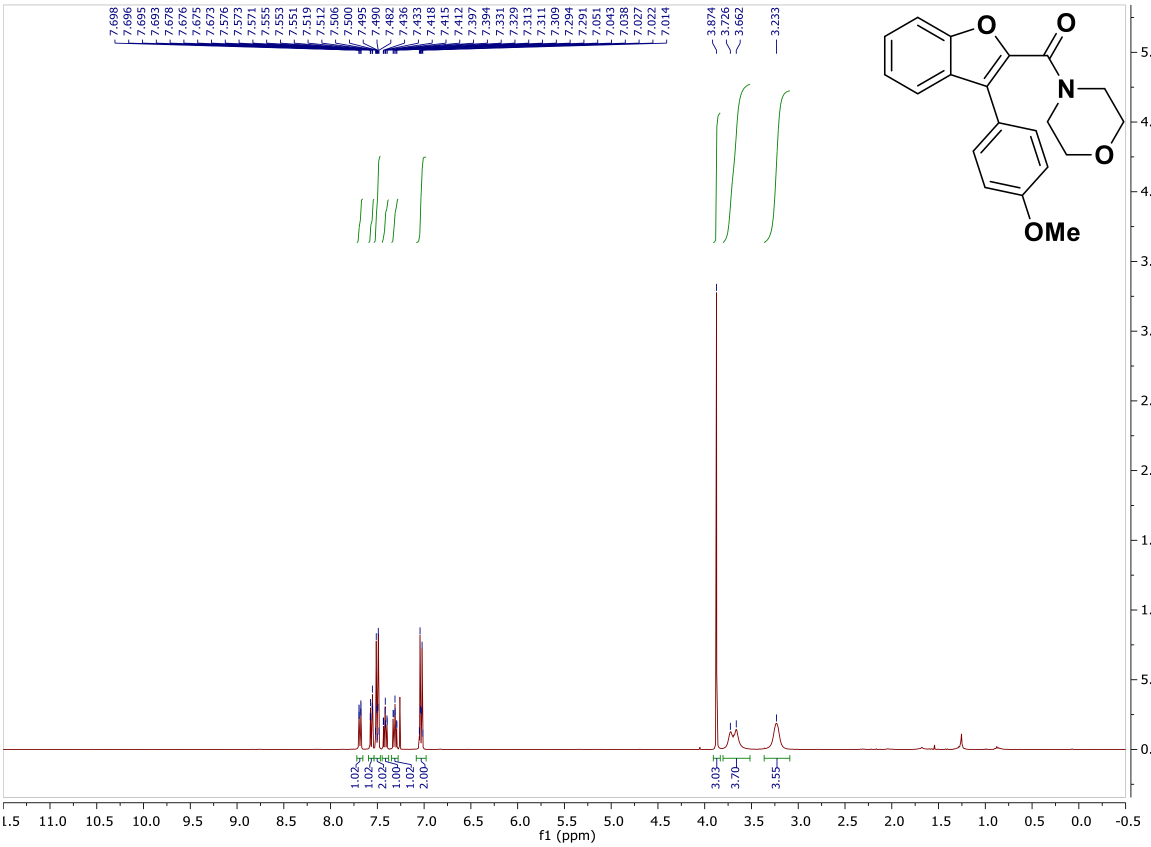

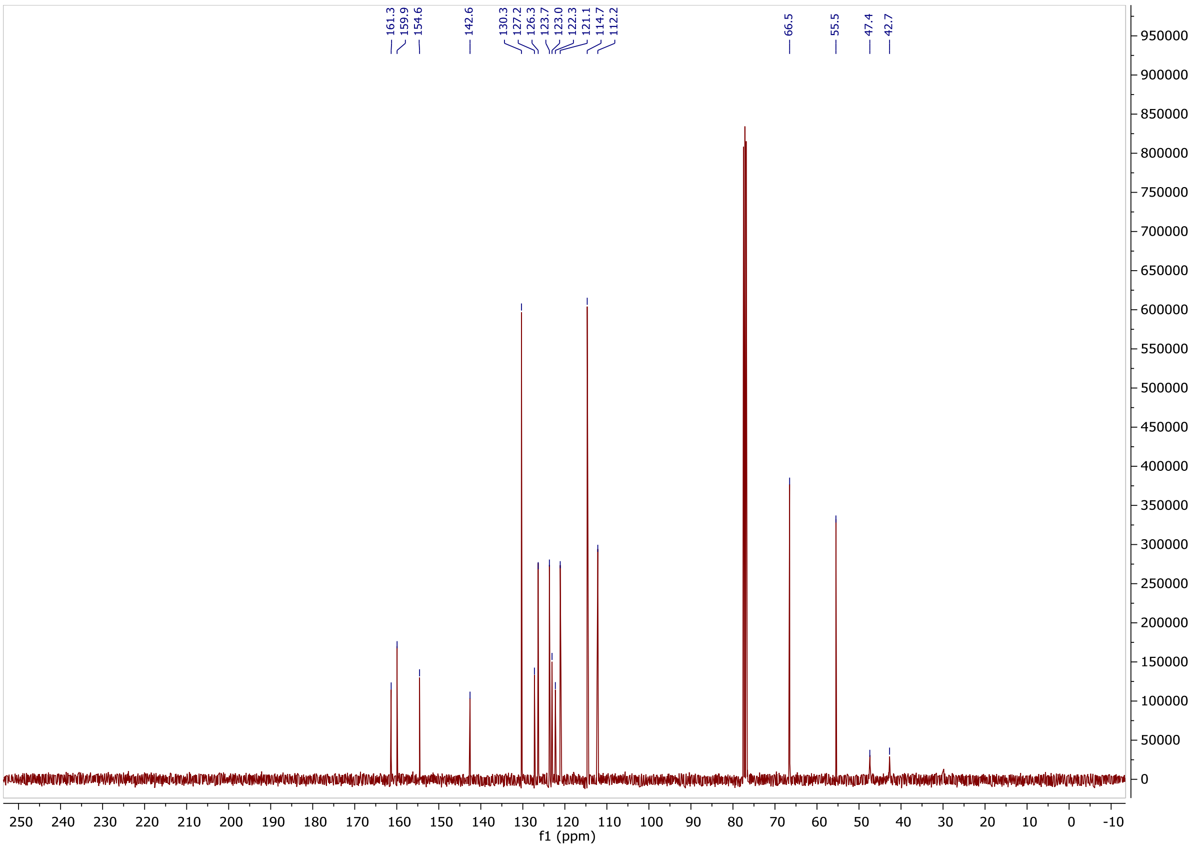

Methyl 4-(2-(benzylcarbamoyl)benzofuran-3-yl)benzoate (**3i**)

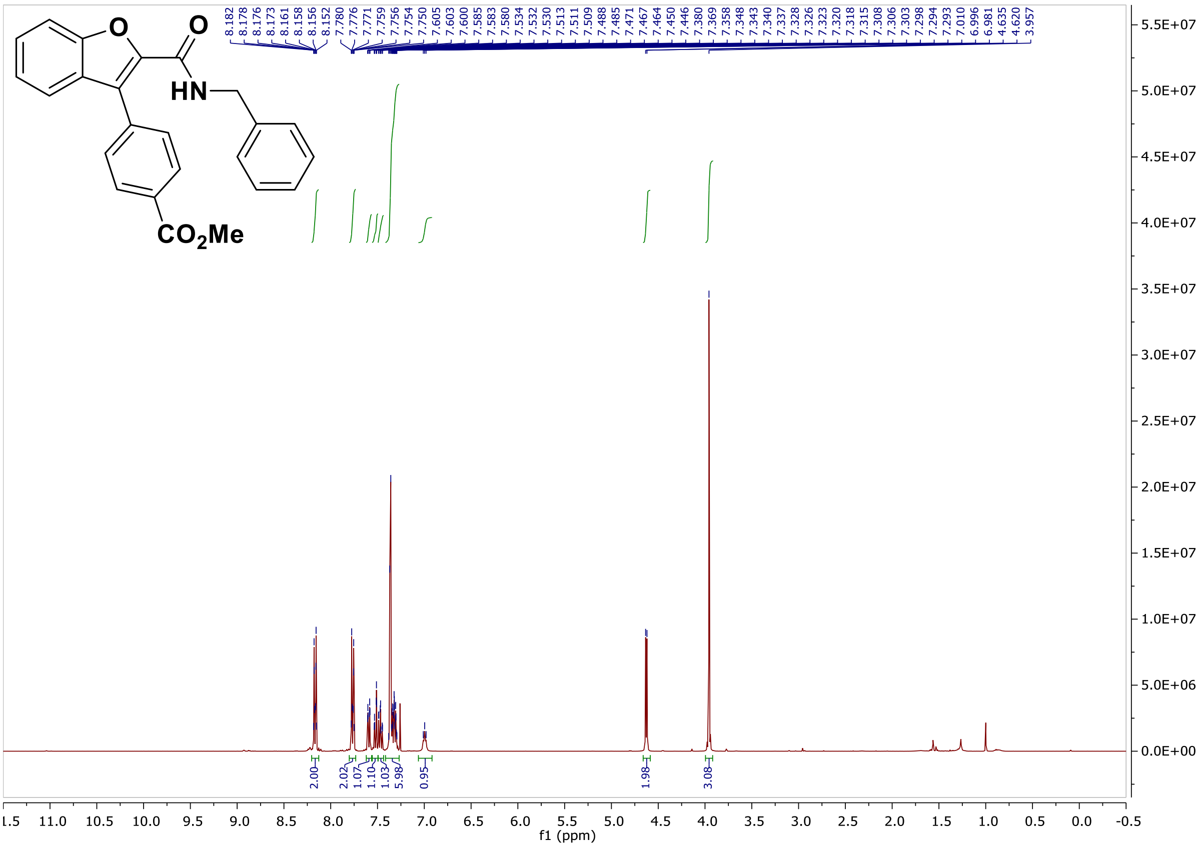

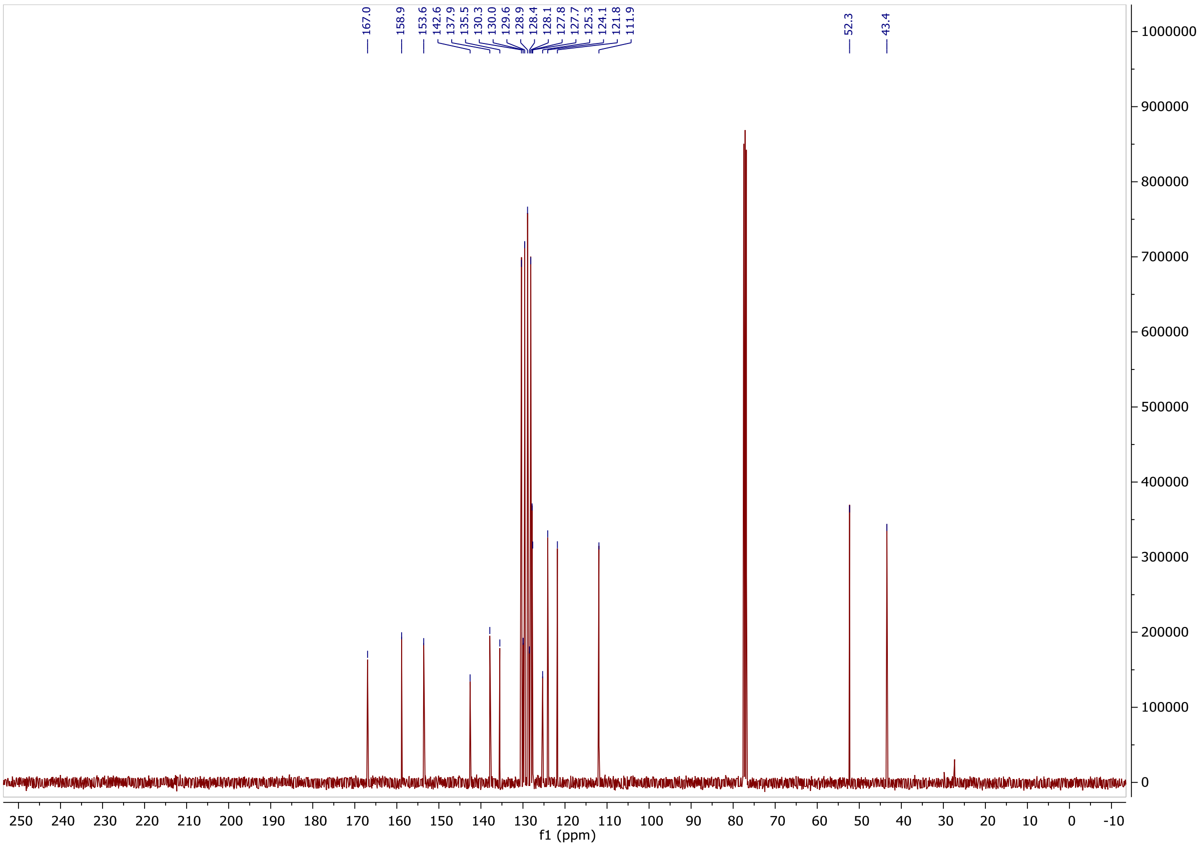

N-Benzyl-3-(thiophen-2-yl)benzofuran-2-carboxamide (**3j**)

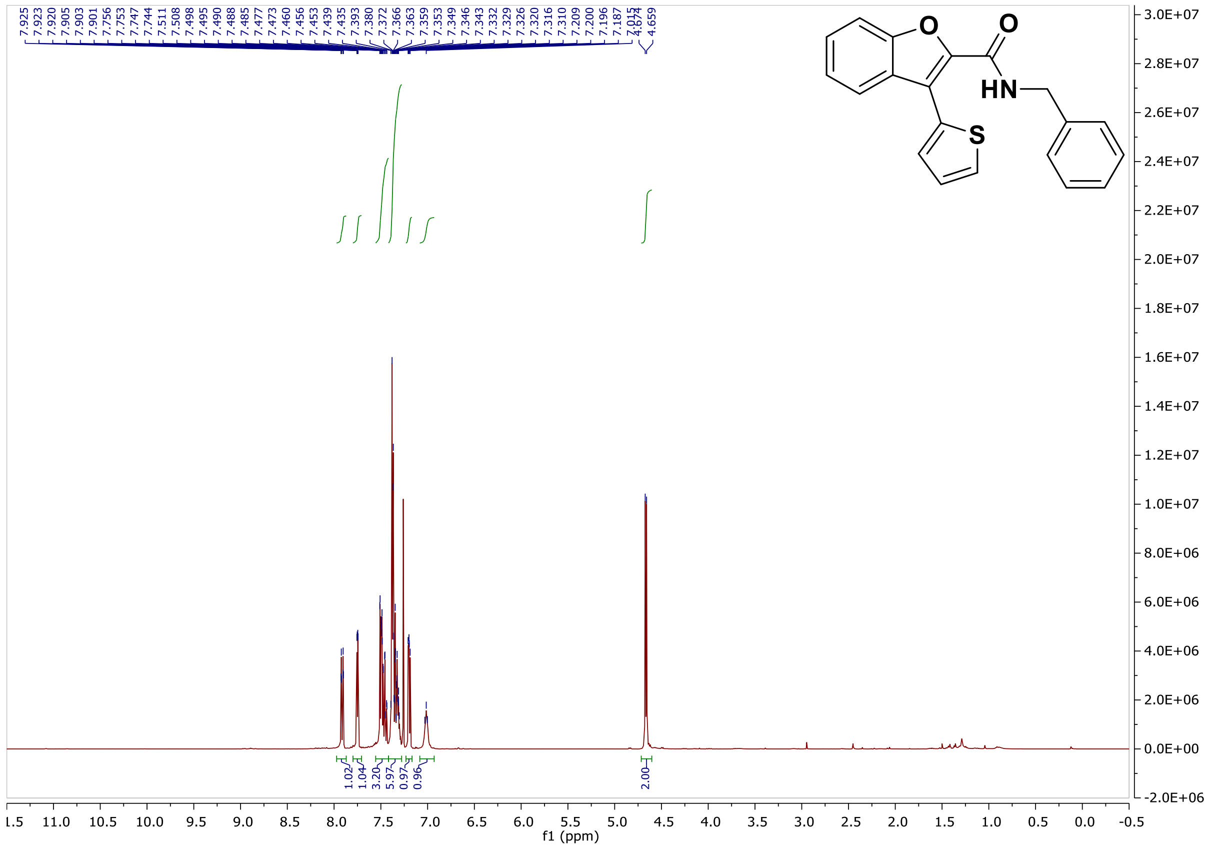

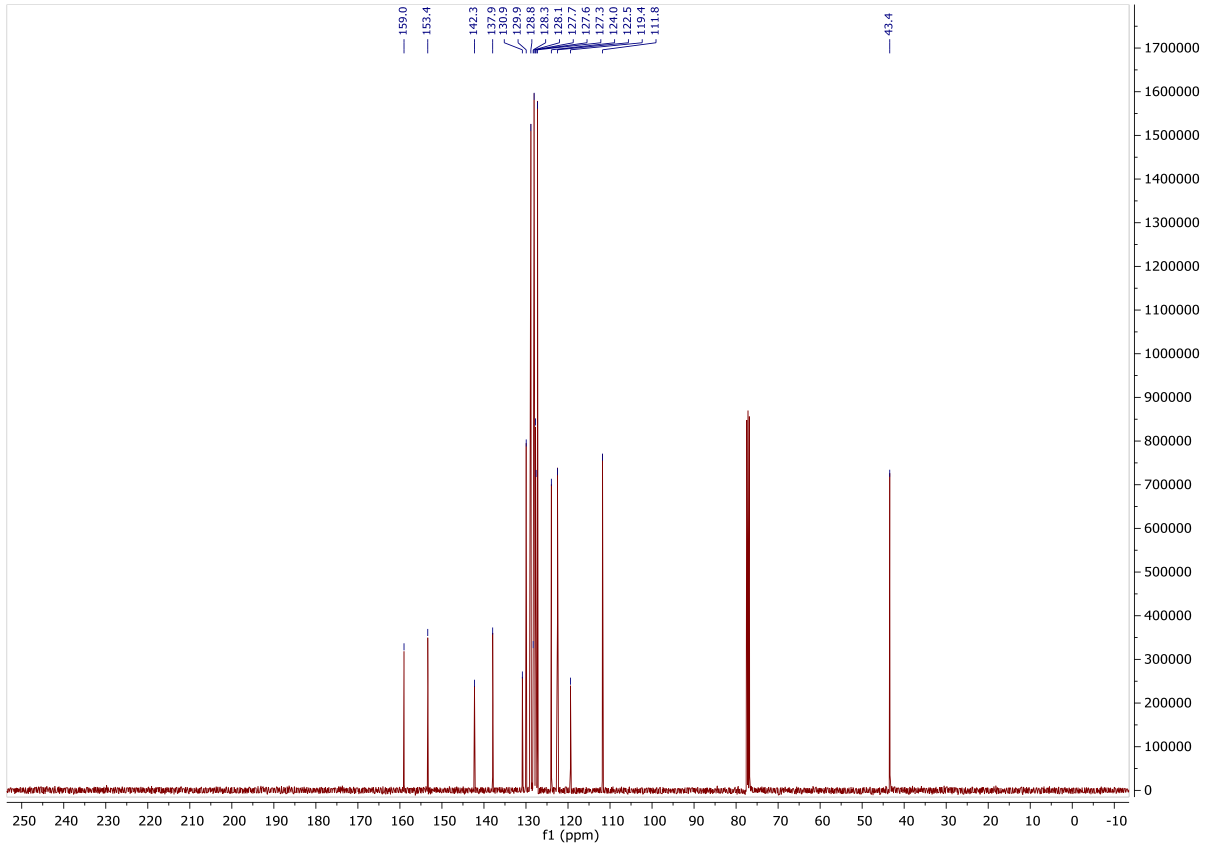

3-(4-Methoxyphenyl)benzofuran-2-carboxylic acid (**4**)

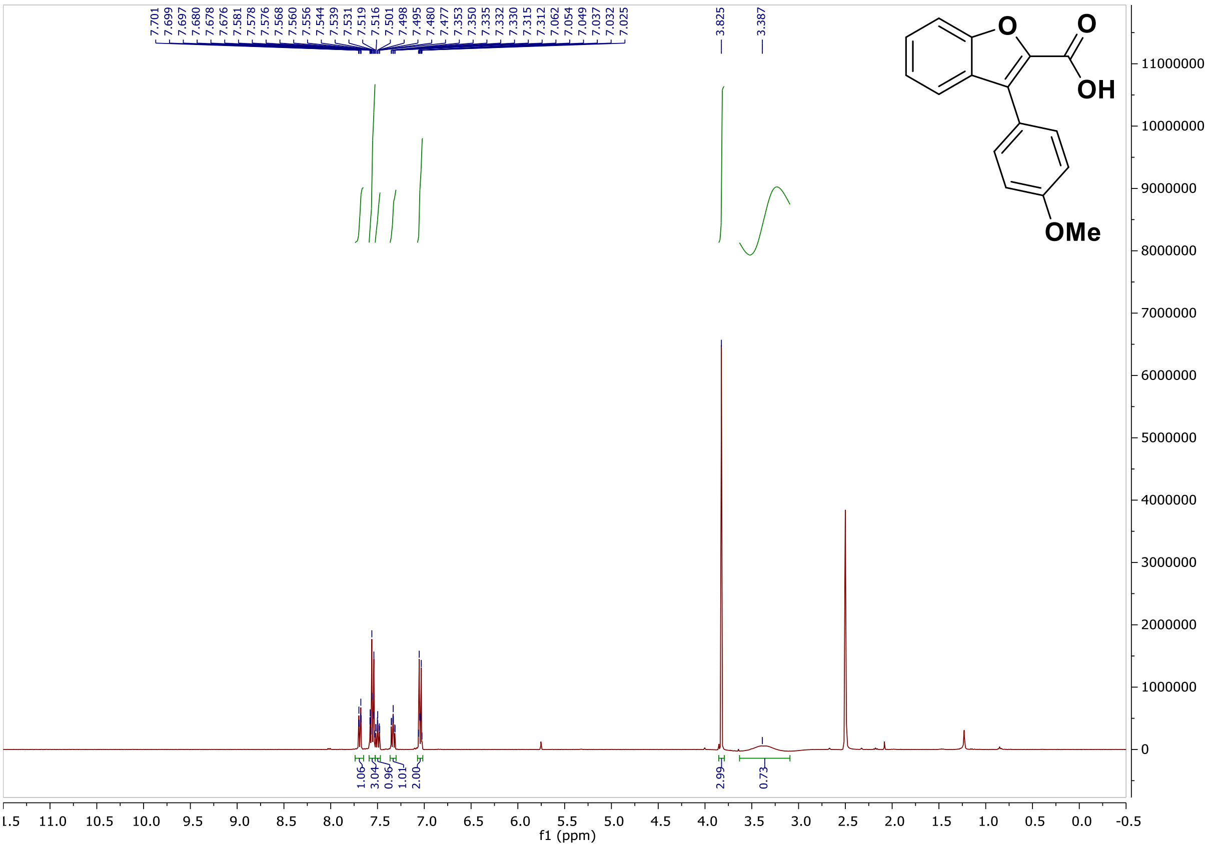

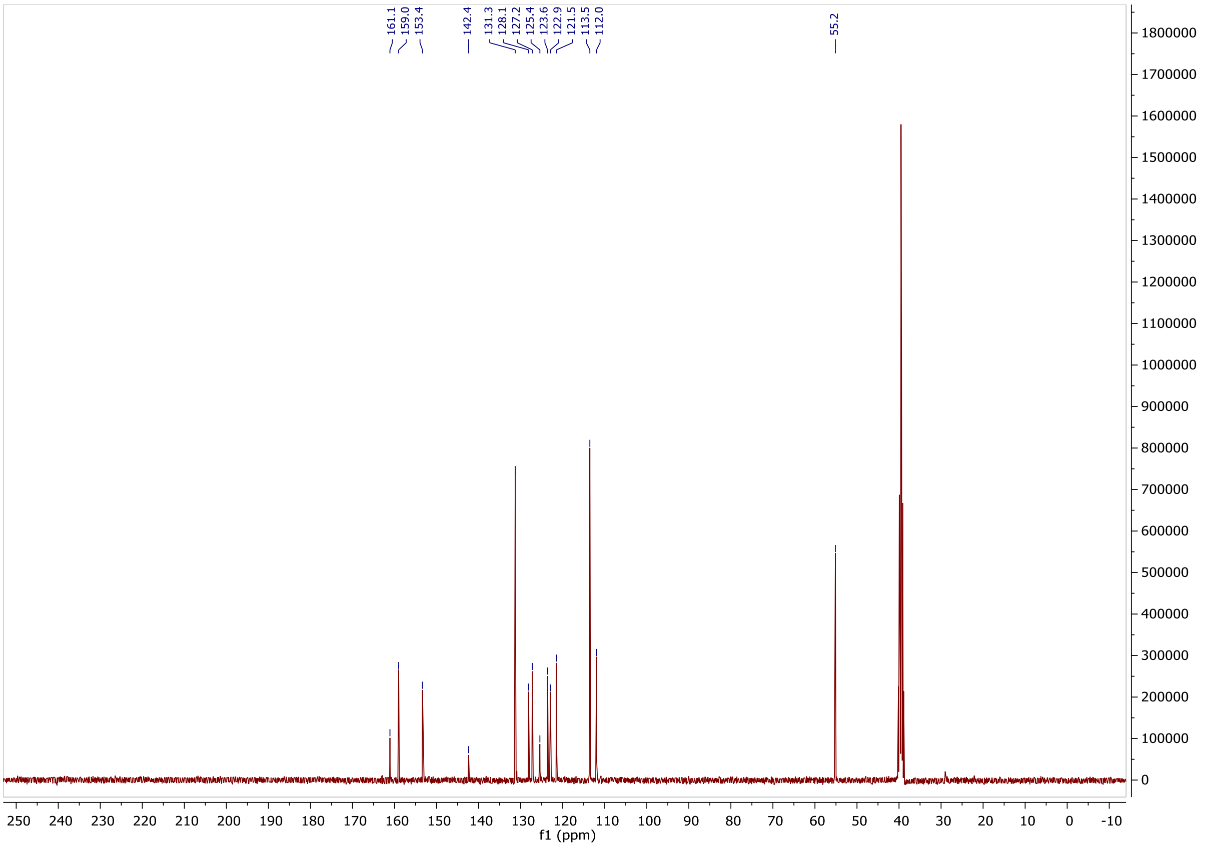

Supplement: Supplementary file 1 [file molecules-25-00361-s001.pdf]
